# Supplementary material for: Psychosocial Determinants of Lifestyle Change after a Cancer Diagnosis: A Systematic Review of the Literature
Source: Cancers (Basel). 2022 Apr 16;14(8):2026. doi: 10.3390/cancers14082026 (PMC9032592; doi:10.3390/cancers14082026)
Supplement: Supplementary file 1 [file cancers-14-02026-s001.zip › Table S10_MH.pdf]

Table S10. Overview of qualitative papers on psychosocial determinants of changes in multiple health behaviors (n=13).

First author, (year), Country	Study design	Sample characteristics	Lifestyle change	Findings on psychosocial determinants
Beckenstein et al. (2021)[125]<br><br>Canada	*Semi-structured individual interviews,* following a lifestyle weight loss intervention.	N=17 breast cancer survivors who participated in a 22-week weight loss lifestyle intervention<br><br>Mean age: 62 ± 8.0 years<br><br>Mean body mass index of 34.0 ± 7.1 kg/m2.<br><br>Participants were in remission for 10 years (±7 years) prior to starting the study.	During the intervention	*Barriers:*<br>-Personal life: events in the participants' personal lives, such as<br>work schedules, doctor's appointments, etc. were reported as higher priorities compared to meeting intervention requirements.<br>-Physical health issues such as fatigue, illness, or body pain reduced motivation<br>-Meal dissatisfaction: not enjoying the meals provided; initial difficulty to consume unfamiliar foods.<br>-Seasonality/ poor weather conditions (ie, ice and snow blizzards), poor driving conditions as obstacles for gym attendance during the winter months.<br>-Unchallenging exercise regimes: feeling demotivated towards the end of the study due to the exercises not being challenging enough (because of having prior exercise experience).<br>-Exercising alone: motivation to perform physical activity challenging without someone else performing physical activity with them.<br><br>*Facilitators:*<br>-*Through education* and guidance received *from the dietitian and the trainers*, participants reported gained knowledge and confidence in their abilities for lifestyle change.<br>-Social support: support from friends and family and intervention team members, and other participants.<br>-Routine and structure provided by the intervention (eating three meals per day and exercising twice per week)<br>-Motivation: the positive study environment, commitment to the study.<br>-Goal-setting: setting weekly goals using the S.M.A.R.T. goal framework (specific, measurable, attainable, relevant, time-based) with the dietitian<br>-Meal-provisioning: food-provisioning eliminated having to think about meal preparation.<br>-Increased self-awareness: Being more self-aware of the time that they were eating and reasons why they were eating and differentiate between physical and emotional hunger (no longer mindlessly consuming calories).<br>-Individualized, supervised exercise sessions: feeling motivated to attend knowing that someone was waiting for them<br>-appreciation of the gradual increase in exercise difficulty<br>-individualized alternative exercises provided by trainers if needed
Er et al. (2017)[106]<br><br>UK	*Semi-structured in-depth interviews*	N=14 African Caribbean prostate cancer survivors, diagnosed less than 5 years at the time of the interview<br><br>Median age 71.5 years<br>Range 52 to 80 years	During a diet and physical activity intervention	*Barriers:*<br>-Uncertain about the therapeutic benefits of lifestyle on prostate cancer recurrence<br>-Considering lifestyle intervention/ lifestyle change as unnecessary because their prostate-specific antigen (PSA) level was kept under control by the treatments they had received.<br>-Health conditions or sports injuries.<br>-Pre-cancer diet and lifestyle: *perceiving no need for lifestyle chang*e as diet and physical activity before a

				prostate cancer diagnosis was already perceived as healthy; already having made positive dietary changes, prompted by other health problems such as diabetes; Men perceived their diet and lifestyle to be healthy<br>-perceived lack of evidence about the link between diet, lifestyle and prostate cancer<br>-Not having received dietary or lifestyle advice from health professionals, or conflicting information/ doctor uncertain about the effectiveness of diet and lifestyle on prostate cancer.<br>-not believing that changing diet and lifestyle have any impact on cancer progression, particularly if their treatment was effective<br>-feeling restricted<br>-being unfamiliar with healthy products/ (dairy substitutes such as soya)<br>-being hesitant to give up habitual food products<br>-being optimistic about prostate cancer and 'take it as it comes', or considering cancer as treated and wanting to move on<br>-side effects from treatments including incontinence (barrier to physical activity)<br>-*financial worries* and loss of income because of a prostate cancer diagnosis could also have a knock-on effect on opportunities for physical activity.<br>-*Older age* as reason for not doing strenuous physical activity; viewing themselves as too old to be playing sports; perceive gentle exercise as safer and more appropriate for their age.<br><br>*Facilitators:*<br>-Perceiving lifestyle changes to be beneficial to health.<br>-Advice from health professionals and social support in coping with prostate cancer.<br>-Lifestyle changes complementing existing diet.<br>-*Prostate cancer diagnosis* and *ageing* heightened awareness of their health, particularly in regards to their body weight.<br>-Prostate cancer diagnosis acted as a catalyst for change.<br>-Receiving information from a trusted source of health information (health professionals/ experts)<br>-Believing that diet and lifestyle are important for cancer prevention<br>-Autonomy: self-initiating changes, being personally motivated<br>-enjoying lifestyle behavior<br>-actively seeking ways to alleviate the experienced side effects (depression, fatigue, pain, incontinence) they experienced and wanting to be fit again.<br>-feeling better through exercise, rather than relying on medication for depression or pain<br>-social networks and support: partners, family and friends acted as a 'change agent'; group exercise provided shared purpose and motivation.<br>-perceiving the ability to do the exercises just as before as a sign of recovery.<br>-Having more awareness of their health as they *aged* because they perceived that they were more susceptible to illness, so having a healthy diet and lifestyle became more relevant to them.<br>-Some men noticed that they put on weight more easily as they *aged*, mainly because they were less active than before. That prompted men to eat less, cut down food portion sizes, alcohol intake or exercise more.
Hackshaw-McGeagh et al. (2017)[107]	*Semi-structured interviews, face to face or via telephone,*	N=16 men with clinically localized prostate cancer, who had	During interventions	*Barriers:*<br>-'modern' digital technology

UK	*individually or together with partner*	recently undergone robotic radical prostatectomy or were undergoing radiotherapy and taking part in nutritional and physical activity interventions, and seven partners.<br><br>Mean age 67 (Range 53-79)		-Poor weather: despite enjoying an activity, poor weather conditions could prevent the activity taking place<br>-Physical limitations/ side effects of treatment, most notably incontinence following radical prostatectomy (fear of leakage).<br>-Not having a partner (anymore) to be physically active with.<br>-Perceiving lifestyle changes as boring or unenjoyable.<br>-Other unrelated health conditions such as knee replacements, arthritis, bowel problems, stroke and heart attacks<br>-Competing time interests/ commitments<br><br>*Facilitators:*<br>-Shock at the time of *diagnosis* resulting in participants taking stock of their current lifestyle behavior, identifying that change may be necessary.<br>-Motivated by reducing mortality and suffering<br>-Family support was considered vital to facilitate change. Family or social support from partner, children or friends (participation of partner in physical activity activity or assist with purchase and preparation of food), providing emotional, informational, tangible support (e.g. providing financial assistance or material goods)<br>-Personal motivation for weight loss (after weight gain) or expectancy to improve general health.<br>-Weight gain, especially if a health care professional had raised the issue.<br>-Recommendations for behaviour change being delivered by a health care professional.<br>-Knowing the reasons for lifestyle changes, understanding the underlying science, or how the specific change would benefit them.<br>-(anticipated) enjoyment of lifestyle changes
Hardcastle et al. (2017)[134]<br><br>Australia	*Semi-structured face to face interviews*	N=24 colorectal cancer survivors who had completed active treatment within the previous 2 years. Participants had existing morbidities that put them at increased risk of cardiovascular disease.<br><br>Mean age = 69.4 years (SD 4.2)<br>Range 63-77<br><br>54.2% Female (n=13)	Following cessation of active treatment for colorectal cancer	*Barriers:*<br>-Participants questioned whether it was worth changing their lifestyle given their life stage and referred to the desire to enjoy life and not having to constantly monitor what you eat<br>-Lay health beliefs: / beliefs about health behavior (foods classified as healthy; sufficiency of exercise)<br>-feeling no need to exercise because their health was under regular surveillance by their physician<br>-Skepticism of eating guidelines: skepticism concerning the links between eating and health (eating 'healthy' food is no protection against cancer)<br>-Lack of motivation: not wanting to put in the effort required to participate (due to underlying failed attempts to change health behaviors; perception of no obvious health benefits)<br>-Lack of confidence in the capability to perform the exercises and in losing weight<br>-Concerns/anxiety related to symptoms and the need to be not far from a toilet affected quality of life and social functioning<br><br>*Facilitators:*<br>-Motivation or self-discipline as a personality trait
Harper et al. (2013)[116]<br><br>USA	*Semi-structured telephone interviews (n=8) and focus groups (n=2, 1 male focus group n=4 & 1 female focus group n=5)*	N=17 older (≥65 years) African American colorectal cancer survivors<br><br>47% female)	Health behavior changes after treatment.	*Barriers:*<br>-difficulties in adopting a healthier diet (i.e., increasing fruits and vegetables) without exacerbating bowel problems (e.g., increased diarrhea) or other health problems (e.g., diabetes management)<br>-misperceptions about what constitutes a healthy diet (e.g., sweet lemonade, soda, no raw vegetables) and/ or what foods should be avoided.

		Mean age of 74.1 (SD=5.87) Range=66–83 years		-Behavioral intentions: no need to change<br>-Lack of self-efficacy: general loss of confidence to make any changes due to concerns about cancer recurrence and feeling more vulnerable to illness, limited mobility, and an inability "to do what I used to do."<br>-fatalistic beliefs (destiny or fate); believing cancer to be predestined, believing that cancer outcomes cannot be changed, also not by an individual's behaviors.<br>-lack of information or having conflicting information about appropriate guidelines for making diet and exercise changes/struggling to make sense of the various pieces of health information, which were often conflicting.<br>-belief that cancer outcomes cannot be influenced though diet and exercise<br>-influence of race/ethnicity in dietary habits of African Americans typical foods eaten, food preferences, and methods of food preparation (e.g., frying pork chops, using ham hocks for flavoring).<br><br>*Facilitators:*<br>-behavior changes with direct and noticeable impact (e.g., minimizing or preventing bowel problems, managing diabetes) were easier to make than changes with less immediate or specific outcomes (e.g., eating better and exercising for overall health).<br>-God and their religious/spiritual beliefs as one motivator of change.<br>-Importance of religion/spirituality in coping with cancer and the challenges of making health behavior changes.<br>-Behavioral intentions: thinking about making changes (e.g., changing what I eat is hard), and intention to change (e.g., I am trying to get more exercise) to actual change (e.g., I exercise regularly)<br>-Self-efficacy: performing specific behaviors increases self-efficacy (e.g., I feel better about myself when I exercise); faith and/or belief in God was an important facet of self-efficacy (e.g., I can do anything with God on my side).<br>-non-fatalistic beliefs: believing cancer outcomes can be changed by an individual's behaviors.<br>-belief that cancer outcomes can be changed through diet and exercise (perceived control)<br>-having a desire for *health information*; educating themselves about health behaviors that might reduce cancer recurrence and prevent and/or treat comorbid conditions; wanting to educate themselves to make good decisions/ adopting healthy behaviors to prevent illness;<br>having more information was described as promoting a greater sense of personal responsibility.
Henry et al. (2016)[126]<br><br>Canada	*Focus groups (n=9)*	N= 29 head and neck cancer patients diagnosed less than three years previously.<br><br>Mean age: 64.6 (SD =10.1)<br><br>21% Female (n=6)<br><br>Mean time since diagnosis:<br>18.7 months (SD =12.3; Range 5.0–44.5 months)	Unspecified	Participants provided a larger than anticipated definition of health behaviors, encompassing both traditional (smoking, drinking, diet, exercise, UVprotection) and Head and Neck Cancer-related (e.g., dental hygiene, skin care, speech exercises, using a PEG, gaining weight).<br><br>*Barriers:*<br>-the radiotherapy treatment itself, and treatment side effects (on vital functions such as eating, speaking, and swallowing, and on physical and mental health); dysphagia, reduced appetite, or reluctance to use the PEG tube for feeding, fatigue, pain, cognitive decline.<br>-*Impaired emotional well-being:* stress and emotional challenges as affecting their capacity to take care of themselves as well as their outlook on life; discouragement, loss of hope, uncertainty (no end in sight and no clear guidelines or information available on the extent to which they would recover), despair, demoralization, and loss of identity and dignity, suicidal thoughts.

	-Other emotional aspects: fear of using the PEG tube, food aversions associated with the pain of radiotherapy, the trauma of PEG insertion/removal, feeling embarrassed and discouraged socially by such treatment side effects as disfigurement, eating in public with the PEG tube, or having a speech impairment.<br>-*Healthcare providers' authoritarian approach* in counseling on Health behavior change inviting opposition and rebellion<br>-Lack of medical information or misinformation, the relationship to the medical team; recommendations from medical team focusing on anticancer treatments, with less emphasis on rehabilitation posttreatment<br>-Difficulty forming new habits, reverting to past habits; difficulties in changing unhealthy *habits*<br>-Social pressures<br>-Lack of information about specific health behaviors as well as clarification regarding the causes of the cancer.<br>-Coping with stress through unhealthy health behaviors (e.g., smoking)<br>-Enjoyment of unhealthy behaviors, and acceptance of the consequences<br>-Physical dependence<br>-having a *poor/uncertain disease prognosis* (felt it was too late to change and needed to "enjoy whatever time was left")<br>-having difficulties transitioning into resuming life-roles<br>-being unaware of how health behaviors were associated with increased morbidity (i.e., increased treatment side effects, lower quality of life, and poorer functional status)<br>-believing that their cancer was "an arbitrary event" and "could happen to anyone"<br>-Feeling a *need for control* over their lifestyle choices at a time when cancer and the treatments had "taken over their lives"<br><br>*Facilitators:*<br>-patient engagement: being proactive in rehabilitation, informed by the medical team, optimistic, flexible, and seeking support when needed.<br>-motivated in order to *return to normal life and reclaim function*, rather than to prevent a cancer recurrence.<br>- *Personality traits of strength and resilience:* being able to stay optimistic and positive in the face of treatment side effects and functional compromises, including the ability to adapt to changing circumstances, to be flexible in transitions, and to maintain determination and perseverance to "get through it" and "return to normal".<br>-Perceiving the diagnosis cancer as a *great wake-up call* to the things that are important in life<br>-Keeping positive through self-talk and remembering what was meaningful to them in life<br>-Having realistic expectations and accepting that "recovery takes time"<br>-*Being open and accepting* what you're going through, accept the present moment.<br>-*information and support from the medical team*: obtaining factual information on all health behaviors in an open, nonjudgmental, and authoritative way, with the healthcare team presenting the rationale to change while ultimately encouraging patients' freedom of choice.<br>-A caring and compassionate medical team, available to listen and offer help, fostered a trusting and nurturing relationship motivating patients to stay engaged in healthy behaviors<br>- resuming basic life functions (e.g., speaking clearly, eating or swallowing solid foods, opening their mouth wider than a certain amount, regaining strength and energy) and

				returning back to life (work, functioning, hobbies, and daily activities)<br>-*social support:* accompanied to radiotherapy and chemotherapy sessions by family members or friends; encouraged by their colleagues at work; helped by supportive partners with meal preparations and household chores<br>-focusing on living, not only on the disease (while undergoing treatment)<br>-facilitator unique to smoking: the cancer diagnosis itself<br>-being aware of the association between WHO health behaviors and cancer recurrence, especially smoking and alcohol
Koutoukidis et al. (2017)[108]<br><br>UK	*N=2 focus groups (n = 5 & n = 3) and individual telephone interviews (n = 8), semi- structured.*	N=16 endometrial cancer survivors; within 5 years post-cancer treatment<br><br>Median age 57.4 years; Range 33-84<br><br>Median BMI 25.8 kg m/2	After completion of treatment	*Barriers:*<br>-No need for lifestyle changes; already following a healthy lifestyle.<br>-Treatment effects, such as fatigue, neuropathy, dizziness, pain, lymphoedema and bowel symptoms.<br>-finding gym exercises boring.<br>-the emotional experience of cancer<br>-Time constraints for physical activity.<br>-Financial constraints were reported for attending a gym and cost was recognized as a barrier for choosing healthy foods.<br>-opportunities offered by the environment (e.g. bad weather, poor infrastructures)<br>-perceiving obesity- related social stigma<br>-Lack of nutrition and physical activity advice from their healthcare professionals posttreatment.<br>-Difficulties finding trustworthy lifestyle information themselves.<br><br>*Facilitators:*<br>-Surviving cancer empowered participants to make healthy lifestyle changes<br>-Perceiving that physical activity could be helpful in symptom management (fatigue, constipation and bladder dysfunction)<br>-Believing that healthy eating and physical activity were critical for overall health and physical functioning<br>-Knowledge about healthy eating and physical activity<br>-Self-monitoring: keeping food/ exercise diary records/ pedometer<br>-Using small, attainable goals<br>-Rewards<br>-Feeling better when physically active<br>-Enjoying group exercises<br>-Engaging in charity events (physical activity events, like walks and marathons)<br>-Opportunities offered by the environment (e.g. good weather, proper infrastructures, owning a dog)<br>-Social support from family and friends (Role modelling by friends; participating in physical exercise groups; family members providing them with healthy options and verbal encouragement).
Shiow-Luan et al. (2017)[148]<br><br>Taiwan	*Qualitative descriptive study: face to face in-depth interviews, semi-structured*	N=13 survivors of any type of cancer, after completion of treatment<br><br>Mean age = 54.3 years Range: 33 to 69 years<br><br>53,8% Female (N=7)	Changes in lifestyle before and after a cancer diagnosis	*Barriers:*<br>-receiving inadequate lifestyle guidance from healthcare professionals for managing their disease.<br><br>*Facilitators:*<br>-Unwillingness to bear the suffering from treatment<br>-Feelings of incomplete responsibility and gratitude toward their family members (feeling responsible to maintain their health; gratitude for receiving encouragement and physical care during the diagnosis and during their cancer treatment);

		Time since cancer diagnosis: 1.5 to 12 years<br><br>Primary diagnoses included hepatoma (n = 6), lymphoma (n = 3), breast cancer (n =2), colon cancer (n = 1), and duodenal cancer (n =1)		to avoid discouraging their family's encouragement and kindness.<br>-Realizing the benefits of lifestyle change in the physical, emotional, and life domains; Feeling the physical and mental Benefits of Lifestyle Changes (feeling more energetic after abstaining from tobacco and alcohol; relief of emotional stress during regular exercise; alleviation of depression)<br><br>-Striving to survive: perceiving that their lives were being threatened and fearing deterioration and disease recurrence, willingness to continue living.<br>-(Particularly participants with a genetic predisposition to cancer), feared deterioration and the recurrence of disease, and having to undergo more stringent medical treatment after the recurrence of disease.<br>-Searching for lifestyle information on their own; proactively searching for various types of information for sustaining their health after having experienced deterioration of their health, including requesting professional advice<br>-Believing that their disease was a consequence of their previous unhealthy lifestyle.<br>-Perceiving regular exercise to be important for disease management<br>-Realizing that smoking and drinking affected health negatively, leading to symptoms of discomfort after the cancer diagnosis.
Somayaji et al. (2019)[117]<br><br>USA	*Semi-structured interviews* after completion of an 8-week lifestyle intervention	N=22 lung cancer survivors<br><br>68.2% female (N=15)<br><br>Median age 60 (Range 40-75)	During intervention	*Barriers:*<br>-Monitoring steps with a pedometer was disappointing/ discouraging because they could not make the expected 10,000 steps per day.<br>-Perceiving dietary information as complicated and difficult to understand because of conflicting information.<br>-Lack of a significant other to talk to about their recovery.<br><br>*Facilitators:*<br>-The diagnosis itself was a motivator for behavior change (wake-up call)<br>-Changing lifestyle helped some patients gain a sense of control over their recovery<br>-Feeling the need to push themselves to make healthy changes even when times were challenging<br>-Self-monitoring behaviors (e.g. using a pedometer): Tracking exercise and diet activities were helpful to facilitate healthier choices, even if they did not meet their goals every day.<br>-Dealing with the consequences of the disease using educational materials provided through the intervention, information from family members and/or guidance, and support gained from their health care providers.<br>-benefit of being able to ask questions and receive encouragement from a trusted expert source; relied on the expertise and availability of the nurse to answer their questions and address their concerns.<br>-Participants expressed feeling personally accountable to the coach which helped the participants make healthier changes and to meet their goals.<br>-The intervention increased their awareness of their health behaviors and subsequent health-related outcomes.<br>-Being provided with simple straightforward information about making healthy diet choices<br>-Finding an enjoyable physical activity besides walking to meet their activity goals.<br>-Structured goal setting<br>-Well-structured diet and exercise plans<br>-Benefit of following the guidelines in reaching their goals.

				-Social support from clinicians, family, and friends, including formal, structured support from clinicians, material support (educational materials), informal support from family members; the coach helped interpret symptoms, which supported self-efficacy and goal setting.<br>-Being self-sufficient/ self-reliant, not needing the support of family or friends to manage their disease, managing their disease without the support of others.
Terranova et al. (2017)[135]<br><br>Australia	*Semi-structured interviews*<br><br>Interviews were conducted (mean ± SD) 7.5 ± 0.5 months after completion of a 12-month weight loss intervention following treatment for breast cancer	N=14 breast cancer survivors<br><br>Mean age 55.6 ± 8.5 years<br><br>Mean time since diagnosis: 17.1 ± 3.4 months<br><br>Mean BMI 30.2 ± 4.6 kg/m2	During intervention	*Barriers:*<br>- Unexpected major family events (e.g. serious illness, death)<br>-breast cancer-specific issues such as post-diagnosis weight gain, treatment-related side effects<br>-Diminished accountability, motivation and interest following the intervention challenged maintenance of behaviour changes (notably dietary self-monitoring).<br>-feeling hungry<br>-having difficulties breaking long-term habits (e.g., eating dessert/at night)<br>-feeling isolated with regard to weight loss<br>-experiencing poor support and understanding from family members.<br>-Spousal dietary habits, specifically relating to decreasing alcohol intake.<br>-eating foods for convenience, palatability or to combat treatment-related side effects such as fatigue.<br>-Treatment-related side effects (difficulty losing weight, sore feet, joint pain, constipation and hot flashes) left women feeling angry and miserable, and demotivated.<br>-Changes in short- and long-term mindset, motivation and focus<br>-Difficulties with self-regulating food intake, particularly related to emotional and compulsive eating patterns.<br>-Receiving conflicting lifestyle advice from health professionals<br>-Difficulties with continued short-term dietary self-monitoring because of: reaching their weight loss goal, repetitious diet, confidence to eat within energy requirements without monitoring, being time poor, and lacking motivation.<br>-difficulties increasing vegetable intake as the recommended serves were excessive, or the recommendation was poorly understood or remembered.<br>-Challenging to maintain lower daily energy intake and continue long-term dietary self-monitoring once contact with their coach ceased or weight loss goals were reached.<br><br>*Facilitators:*<br>-Structure and support of the intervention<br>-Guidance, encouragement and regular contact from their coach<br>-Feeling accountable to the intervention and coach<br>-Forming habitual behaviours (e.g., habitual physical activity)<br>-Self-monitoring (increased behavior awareness); Dietary self-monitoring also facilitated physical activity for some women who used their diary to record daily activity<br>-Portion control<br>-Perceived motivation to participate in the intervention: to lose weight and prevent recurrence<br>-the structure returning to work provided (decreasing opportunities for unplanned meals, snacking in-between meals)<br>-Being responsible for and influencing dietary behaviours of family members (in particular children)<br>-Support from family members and/or friends<br>-Improved dietary knowledge

				-Increased motivation or self-esteem and being in the right mindset during the intervention<br>-Physical activity positively impacted psychological factors such as mindset<br>-Recognising the importance of self-monitoring<br>-Being confident they could restart self-monitoring if needed
Vance et al. (2017)[127]<br><br>Canada	*Semi-structured qualitative interviews*	N=28 female early-stage breast cancer survivors within 12 months of completing chemotherapy treatment.<br><br>Mean age: 49.8 (SD 8.5) Range 33-69<br><br>Mean time from treatment 56.4 (SD 4.4) Range 0.5-13 months	During chemotherapy	*Barriers:*<br>-Physical side effects of treatment, including persistent, overwhelming fatigue, persistent nausea or extended periods of vomiting, constipation, heartburn<br>-Side effects of treatment persisted into the second and third weeks of the chemotherapy cycle, allowing for a very short window of time in which they could eat normally before the next cycle.<br>-The acute side effects of treatment lasted longer with each cycle, suggesting a cumulative effect across the treatment.<br>-Treatment-related factors including fatigue, taste changes (metallic/chemical taste or no taste), and gastrointestinal disturbance (nausea, constipation, and heartburn).<br>-Psychosocial influences including anxiety, stress<br>-Coping with the emotional impact/ emotional distress: increased intake of energy-dense comfort foods<br>-Taste changes associated with both food cravings and food aversions<br>-Responses to nausea ranged from decreased appetite and lower food intake, to reports of increased appetite and eating more frequently throughout the day in an effort to relieve symptoms.<br>-Living alone: women who lived alone expressed that fatigue had interfered with their ability or motivation to prepare meals, and they had tended to rely on prepackaged, processed foods for convenience.<br>-In women who had gained weight during treatment: taste changes, nausea, emotional distress (anxiety and stress), led to increased appetite, food cravings, consumption of comfort foods, and increased portion sizes, which led to increased energy uptake (overeating and/or increased their intake of energy-dense comfort foods) which led to weight gain<br>-Masking bad taste by eating in response to taste changes in women who gained weight during treatment<br>-In women who had lost weight during treatment: Severe and persistent side effects of treatment (Nausea, constipation, fatigue, and heartburn) led to decreased appetite, a day-to-day variability in food tolerance and more difficulty in selecting foods that were appealing and enjoyable, especially in those living alone (without cooking support), which led to a decreased energy intake (prolonged reduction in food intake or irregular eating patterns) leading to weight loss during treatment
Wu et al. (2015)[118]<br><br>USA	*Focus groups and individual interviews (n=3)*<br><br>Survivors and supporters attended separate focus group sessions (5 survivor focus groups, 5 supporter focus groups)	N=25 adolescent and young adult (AYA) cancer survivors who completed active cancer therapy and N=19 supporters<br><br>Mean age 27.6 (SD 6.6) Range 18–39<br><br>Mean age at cancer diagnosis: 17.3 (SD 12.2)	Unspecified	*Barriers:*<br>-Lack of resources: financial, professional services, particularly for those who had spent significant sums of money on their cancer treatment or who were not yet financially independent; relatively high costs of fresh food, paying for exercise facilities, programs, and professionals that would facilitate exercise.<br>-Lack of information from professionals/ legitimate resources to guide their exercise and eating<br>-Negative thoughts and feelings: feeling depressed, embarrassed, and frustrated about not being able to do physical activities in the same way as before cancer.<br>-Fatigue prevented them from exercising, particularly for survivors who had children.

		Type of cancer: leaukemia/lyphoma n=10; solid tumor n=12, brain tumor n= 3)<br><br>68% female (N=17)		-family, friends, and co-workers not having the same health goals<br>-challenge of having unhealthy snacks available at work meetings<br>-finding healthy dishes to eat at social gatherings<br>-feeling isolated at social events<br>-Wanting a break from worrying about health<br>-Hearing excessive concerns or unsolicited advice were burdensome and frustrating.<br><br>*Facilitators:*<br>-Cognitive motivators including body image.<br>-The belief that engagement in health behaviors would prevent future health problems, including cancer, or help them regain control over current health problems.<br>-Fear, e.g., fear of dying from a health problem motivated to make healthy lifestyle changes.<br>-Having a goal and routine (e.g., registering for a running race, encouraged to run regularly).<br>-Benefit of trying different types of activities to maintain motivation<br>-increased access to fresh fruits and vegetables via gardens<br>-gym memberships<br>-wellness programs<br>-skill-building, e.g. in food preparation and meal planning<br>-Companionship of friends and family<br>-being more motivated to cook healthy food when others, such as family and especially children, relied on them.
Yufe et al. (2019)[128]<br><br>Canada	*Longitudinal interview design; interviews were semi-structured via telephone*	N=4 Breast Cancer survivors were studied intensively through longitudinal interviews conducted at four time points with each participant: (1) pre-treatment, (2) mid-way treatment, (3) post-treatment, and (4) three-months following their participation 12-week group-based intervention.<br><br>Participants completed primary treatment within the last five years, self-reported weight increase of five or more pounds' post-treatment, regardless of normal or overweight BMI before diagnosis or overweight status since or before diagnosis	During intervention and up to 3 months follow-up	*Barriers:*<br>-Struggling with the perceived medical pressure to "lose weight or die."<br>-Experiencing a complete loss of control over eating habits and activity choices post-treatment<br>-Feeling a lack of efficacy; pressuring herself to exercise had a paradoxical, paralyzing effect<br>-Balancing motherhood with maintaining a healthy lifestyle: the tension between caring for oneself and caring for others made lifestyle accommodation challenging; sacrificing family time for personal health reasons (i.e., for exercise and food preparation) and associated feelings of guilt for taking time for oneself; feeling family and work schedules were "too hectic."<br>-Not being able to find a babysitter that would allow for engaging in physical activity<br>-Internal forms of resistance (directed toward oneself): personal inner conflicts prevent from overcoming obstacles to improving health.<br>-External form of resistance (directed externally): passively surrender when met with the challenge of altering lifestyle (related to a history of unsuccessful attempts to lose weight, protecting from further disappointment)<br>- External form of resistance (directed externally): expressing explicit opposition (expressing autonomy) in response to healthcare providers being prescriptive about making lifestyle choices, which was experienced as an imposition forced upon.<br><br>*Facilitators:*<br>-to prevent cancer recurrence.<br>-improve low mood, and quality of life through adoption of a healthy lifestyle.<br>-setting clear goals<br>-struggling with a loss of identity due to the diagnosis, but while participating, narrated her experience with positivity and hope for the future.<br>-Weight management prescription as a motivator

	-motivated by the scientific evidence linking behavior change to reduced rates of cancer recurrence<br>-Active engagement in the program increased experience of personal control in relation to cancer, which in turn helped her feel more like herself again.<br>-The shift in meaning of healthy lifestyle behaviors pre-to-post<br>cancer from feeling "good" and looking "great" to "being healthy"<br>-Finding a balance between motherhood and maintaining a healthy lifestyle: Engage with their children in physical activities, which provided a practical solution to the dilemma of having to sacrifice quality family time in order to adopt more healthful habits.<br>-Development of a more self-compassionate perspective toward adopting a healthy lifestyle: being more flexible and forgiving; if one of her exercise schedules did not "stick," then she would be open "to trying something else" instead of giving up on herself.<br>-Openness to re-framing attitudes in terms of how they conceptualized and thought about lifestyle modification after breast cancer.
