# Supplementary material for: Psychosocial Determinants of Lifestyle Change after a Cancer Diagnosis: A Systematic Review of the Literature
Source: Cancers (Basel). 2022 Apr 16;14(8):2026. doi: 10.3390/cancers14082026 (PMC9032592; doi:10.3390/cancers14082026)
Supplement: Supplementary file 1 [file cancers-14-02026-s001.zip › Table S1_MH.pdf]

Table S1. Overview of quantitative studies assessing psychosocial determinants of changes in exercise/physical activity among cancer survivors (n=45).

First author (year) Country	Study design	Sample Characteristics *(at baseline)*	Psychosocial variables *Type(s); Assessed at; Assessed with*	Lifestyle *Assessment instruments Baseline; Change (period)*	Findings	Comments
Alfano et al. (2009)[26] USA	Cross-sectional descriptive study	227 female long-term *breast cancer* survivors<br><br>Mean (SD) age 61.9 (9.9) years<br><br>Mean (SD) time since diagnosis 12.4 (1.8) years	*Inter-individual:* Social support (MOS Social Support Survey)<br><br>*Intra-individual:* Depressive symptoms (Center for Epidemiologic studies – Depression Scale); fear of recurrence (modified form of the Breast Cancer Anxiety and Screening Behavior Scale); fatigue (vitality subscale of the Medical Outcomes Study Short Form-36); stressful life events (11-item Life Events Scale); satisfaction with sexual functioning (Watts Sexual Functioning Questionnaire); body satisfaction (Self-Concept Scale)	Changes in PA was measured by asking the participants whether their exercise habits increased or decreased following diagnosis. Changes in PA were dichotomized in to "change in the direction of better health", and "no change or change not reflecting better health".<br><br>Baseline values for PA are not mentioned.<br><br>32% reported increasing PA and 68% reported decreasing or not changing PA.	*Inter-individual:* Changes in PA was found to have borderline significant association with social support (p=.06), with respondents reporting increasing their PA following diagnosis reported greater *social support* than survivors who reported decreasing or maintaining their pre-diagnosis level.<br><br>*Intra-individual:* Changes in PA was found to have a significant association with fatigue (p=.03), with respondents reporting increasing their exercise reported less fatigue.<br><br>All other variables were NS.	
Barbosa et al. (2019) [87] Portugal	Prospective cohort study	428 women newly diagnosed with *breast cancer* and proposed for surgery<br><br>50.7% had less than 55 years of age at baseline	*Socio-demographic:* Educational level, age, employment status, and marital status<br><br>*Intra-individual:* Anxiety and depression (Hospital Anxiety and Depression Scale)	PA both pre-diagnosis and post-diagnosis was measured using an interview at 3-year follow-up. PA was dichotomized according to their adherence to the recommendation for cancer prevention "being physically active" (practicing any sports or doing physical exercise).<br><br>Non-adherence: 82.7% pre-diagnosis 83.9% 3-year follow-up.<br><br>54.1% of those being physically active pre-diagnosis reported changing to not being physically active after diagnosis, while 9.9% of those not being physically active pre-diagnosis reported	*Socio-demographic:* Respondents with a higher *educational level* (OR= 11.53, 95% CI: 2.20 – 60.53) and older *age* (OR= 4.71, 95% CI: 1.17 - 18.99) were significantly more likely to change towards being physically inactive post-diagnosis. Among breast cancer patients who were physically inactive before diagnosis, older women had a significantly lower odds of being physically active at 3 years after diagnosis (OR= 0.32, 95% CI: 0.14–0.75).<br><br>Other variables were NS.<br><br>*Intra-individual:* NS	

				change towards being physically active post-diagnosis.		
Basen-Engquist et al. (2013)[27]<br><br>USA	Intervention study<br><br>6-month home based *exercise* intervention	100 women who had been diagnosed with Stage I, II, or IIIa *endometrial cancer* and were at least 6 months posttreatment.<br><br>Mean age 57.0 years<br><br>Mean time since diagnosis 26.0 months<br><br>Mean BMI 34.2 kg/m^2	*Intra-individual:*<br>Social-cognitive theory (SCT) variables: exercise self-efficacy, barriers self-efficacy, positive outcome expectations, and negative outcome expectations<br><br>Ecological Momentary Assessments were conducted by using hand-held computers for 10- to 12-day periods every 2 months (baseline, 2, 4 and 6 months). Participants rated morning self-efficacy and positive and negative outcome expectations by using the computer, recorded exercise information in real time and at night, and wore accelerometers. At the midpoint of each assessment period, participants completed self-composed SCT questionnaires.	Exercise was measured using a combination of methods: EMA questions answered at the time of exercise (real-time exercise minutes), EMA questions answered at the end of the day about exercise completed that day (nighttime exercise diary minutes), minutes of moderate or greater activity performed in bouts of at least 10 min as measured with the accelerometer, and the Community Health Activities Model Program for Seniors (CHAMPS) questionnaire.<br><br>Mean (SD) min exercise per day<br>Baseline: 14.5 (18.5)<br>2 months: 19.3 (20.5)<br>4 months: 18.4 (20.8)<br>6 months: 17.6 (20.7)	Authors tested whether the SCT theory variables were associated with the mean exercise duration per day at subsequent sessions (2, 4, or 6 months), controlling for baseline exercise minutes.<br><br>Of the questionnaire measures of SCT variables, only *exercise self-efficacy* predicted exercise at the next time point (p= .003).<br><br>The consistency of the relationship between *self-efficacy* and exercise minutes over short (same day) and longer (Tj to Tj-1) time periods provides support for a causal relationship.	Women were excluded from this study if they met the public health recommendations for physical activity (moderate or greater intensity on at least 5 days per week for 30 min or more, or vigorous intensity activity for 20 min or more on at least 3 days per week; American College of Sports Medicine, 1998) and had maintained that level of activity for 6 months or longer.
Bennett et al. (2007)[88]<br><br>New Zealand	Intervention study<br><br>6-month Motivational interviewing intervention	56 physically inactive adult cancer survivors were at least 6 months posttreatment (intervention: n= 28, control: n= 28).<br><br>76.8% *breast*, and 23.2% other<br><br>Mean time since completion of treatment 42 months<br><br>Age 37-85 at baseline<br>89.3% female<br><br>Intervention group<br>Mean (SD) age 55.5 (8.9)<br>Mean (SD) BMI 29.6 (6.4) kg/m^2<br><br>Control group:<br>Mean (SD) age 60.1 (11.0)	*Intra-individual:* Self-efficacy (6-item measure of self-efficacy for PA)	PA was assessed at baseline, at 3 months and at 6 months using the Community Health Activities Model Program for Seniors.<br><br>Intervention group mean (SD) caloric expenditure per week (kcal/week)<br>Baseline: 1,929 (1,233)<br>3 months: 2,571 (1,972)<br>6 months: 3,484 (2,445)<br><br>Control group mean (SD) caloric expenditure per week (kcal/week)<br>Baseline: 2,856 (1,233)<br>3 months: 2,651 (1,590)<br>6 months: 3,252 (2,308)	*Intra-individual:*<br>Participants with higher *self-efficacy* in the intervention group increased their PA more over the 6-month intervention period than participants with lower *self-efficacy* (b = 121.35, p < .05). In the control group, *self-efficacy* did not affect levels of PA.	Participants were included only when they were underactive (engaged in exercise less than three days a week for 20 minutes) and were willing to try to increase their PA.<br><br>Control condition consisted of intervention without motivational interviewing content

		Mean (SD) BMI 26.6 (7.2) kg/m^2			
Bergengren et al. (2020)[89]<br><br>Sweden	Cross-sectional nation-wide, population based study	1288 low-risk *prostate cancer* survivors<br><br>Mean age 63 years at diagnosis (range 59-66)	*Intra-individual:* Health related quality of life (Expanded Prostate Cancer Index Composite 26 (EPIC-26))	PA change was assessed using a questionnaire composed of study-specific questions, by asking whether their PA behavior changed after receiving the diagnosis. PA change was categorized in to "I exercise less", "Unchanged", and "I exercise more".<br><br>14.7% reported increasing PA<br>3.6% reported decreasing PA<br>71.2% reported not changing PA.	*Intra-individual:* Having poor health related *quality of life* on two or more domains was found to be associated with exercising less (OR = 1.6, 95% CI: 1.2 - 2.1).	
Bloom et al. (2008)[28]<br><br>USA	Intervention study<br><br>3-month socio-educational intervention	404 5 female *breast cancer* survivors aged 50 or younger at diagnosis who were 5 years from diagnosis (intervention: n= 201, control: n= 203)<br><br>42% under the age of 45 at diagnosis	*Socio-demographic:* Race/ethnicity, marital status, age, employment status, and educational level<br><br>*Inter-individual:* Social support (Berkman-Syme Social Network Index)	At baseline participants were asked to report the number of days per week they were physically active for at least 30 min, and to indicate how often they engaged in various forms of exercise and PA. Post-intervention participants reported whether they had made changes in PA in the past 6 months.<br><br>Intervention group: 71% reported exercising 2 or more days/week at baseline 57% reported increasing PA<br><br>Control group: 77% reported exercising 2 or more days/week at baseline 47% reported increasing PA	*Inter-individual:* Participants having more *social support* were more likely to have increased PA post-intervention (OR = 1.38, 95% CI: 1.10 – 1.74).<br><br>*Socio-demographic:* All variables were NS.	Control condition consisted of a delayed intervention group
Brunet et al. (2014)[64]<br><br>Canada	Prospective cohort study	199 women who had been diagnosed with stage I-III *breast cancer* and who had completed primary treatment within the previous 20 weeks.<br><br>Mean (SD) age 55.01 (10.96) years<br><br>Mean (SD) time since completion of	*Socio-demographic:* Age<br><br>*Intra-individual:* Depressive symptoms (Center for Epidemiological Studies Depression scale), fatigue (Profile of Mood States), and cancer worry (Assessment of Cancer Concerns scale)	PA was assessed at baseline, 3 months, 6 months 9 months and 12 months using the Leisure Time Exercise Questionnaire.<br><br>Five distinct trajectories of PA change were distinguished: consistently inactive (mean$_{Time1-Time2}$ = 0 min/week), decreasing levels (Time 1: 108.13	Trajectory groups that did not meet MVPA guidelines were combined and compared to trajectory groups meeting guidelines consistently over time.<br><br>*Socio-demographic:* NS<br><br>*Intra-individual:* Respondents who reported higher levels of *depressive symptoms* (OR= 0.49, 95% CI: 0.24 – 0.98), and	

		treatment 3.46 (2.33) months<br><br>Mean (SD) BMI 26.31 (5.65) kg/m^2		min/week, time 5: 0 min/week), inactive with increasing levels (time 1: 0 min/wk, time 5: 221.25 min/wk), somewhat active (mean levels varied between 98.8 and 124.41 over time), and consistently sufficiently active (mean levels varied between 227.89 and 302.49 min/wk over time)	*fatigue* (OR= 0.67, 95% CI: 0.43 – 0.99) at baseline were less likely to remain consistently sufficiently active. Respondents that reported higher levels of *cancer worry* were more likely to remain consistently sufficiently active (OR=1.53, 95% CI: 1.03 – 2.27).
Brunet et al. (2013)[65]<br><br>Canada	Prospective longitudinal study	150 women who had been diagnosed with stage I-III *breast cancer* and who had completed primary treatment within the previous 20 weeks<br><br>Mean (SD) age 54.41 (10.87) at baseline<br><br>Mean (SD) time since completion of treatment 3.94 (3.08) months<br><br>Mean (SD) BMI 25.83 (5.10) kg/m^2	*Intra-individual:* Motivational regulations (The Behavioral Regulation in Exercise Questionnaire-2)	PA was assessed using an accelerometer for 7 consecutive days at baseline, 3 months and 6 months.<br><br>Baseline values are not mentioned.<br><br>Mean (SD) min 3 months: 169.39 (51.82) min light PA and 17.47 (12.77) min MVPA<br><br>6 months: 172.60 (51.85) light PA 17.23 (13.96) MVPA	*Intra-individual:* Changes in self-determined *motivation* was positively related to changes in MVPA (p<.05). The other subscales of motivational regulations (amotivation, external regulation and introjected regulation) were NS.
Castonguay et al. (2017)[66]<br><br>Canada	Prospective longitudinal study	149 women who had been diagnosed with stages I-III *breast cancer* and were approximately 9 months posttreatment<br><br>Mean (SD) time since diagnosis 16.46 (9.46) months<br><br>Mean (SD) time since treatment 9.48 (8.41) months<br><br>Mean (SD) age 55.34 (10.5) years<br><br>Mean (SD) BMI 25.86 (5.33) kg/m^2	*Intra-individual*: Guilt and shame (Weight- and Body-Related Shame and Guilt scale), and motivational regulations (external, introjected, and intrinsic motivational regulations; Behavioral Regulation in Exercise Questionnaire-2)	MVPA was assessed with the screening tool of Prochaska, Sallis, and Long[10] at baseline and six months later. To obtain a measure of MVPA change, a residual was calculated in a regression analysis by predicting time 2 levels by time 1 levels and saving the coefficients.<br><br>Mean (SD) days with >30 min MVPA Baseline: 1.08 (1.51) 6 months: 0.81 (1.22)<br><br>Moderate to vigorous PA significantly decrease during the 6 months (t=-9,13, p<.001, d=0.57)	*Intra-individual:* The article mentions only body-related shame to be a significant predictor of changes in MVPA. However, the results depicted in the tables do not confirm this result.
Chambers et al. (2009)[72]<br><br>Australia	Prospective longitudinal study	978 *colorectal cancer* survivors (valid data for all time points)<br><br>44.2% female	*Intra-individual*: anxiety, depression, and somatization (Brief Symptom Inventory–18), and cancer threat appraisal (Constructed Meaning Scale).	PA (Total weekly PA) was assessed using questions based on the Active Australia Survey at 12, 24, and 36 months postdiagnosis.	*Intra-individual:* Respondents whose *somatization* increased between two time periods were less likely to increase their physical activity over the same period (P< .001).

		Age 20-80 at diagnosis		No baseline values mentioned<br><br>A measure of change in physical activity between each survey was generated for the 12-, 24-, and 36-month surveys reflecting the difference (in minutes) between the reported level of PA in the preceding survey and the current survey: Change values were categorized into "No increase" (includes decreasing PA levels), "Increased by 2 hours per week or less," and "Increased by more than 2 hours per week."	Respondents with higher *anxiety* at one time period were less likely to have increased their activity at the next assessment (P= .004). D*epression* or *threat appraisal* were NS.
Cole et al. (2021)[73]<br><br>Australia	Cross-sectional	358 adults receiving treatment for cancer<br><br>81.4% *breast*, 5.2% *prostate*, 3.8% *lymphoma* and related, 9.6% other.<br><br>Mean (SD) age 56.48 (10.76) years<br><br>Mean (SD) time since diagnosis 2.25 (2.62) years	*Intra-individual*: illness perceptions (Revised Illness Perception Questionnaire), and exercise beliefs (exercise self-efficacy, benefits of exercise, barriers to exercise and negative impact on cancer; 20-item questionnaire adapted from Getch et al. (1996)[13])	Exercise level was assessed using the Godin Leisure-Time Activity questionnaire. Change in PA was determined comparing post-diagnosis PA to pre-diagnosis PA. Participants were categorized to be decreasers, increasers or maintainers.<br><br>58.1% were decreasers, 30.4% increasers and 9.2% maintainers.	*Intra-individual:*<br>*Self-efficacy* significantly differed between exercise groups (F(2345)=5.40, p=.005, ηp^{2}=.03), with decreasers reporting lower self-efficacy for exercise than increasers (F(1345) = 8.69, p=.003, d=.31) and maintainters (F(1345) = 4.52, p=.034, d=.38). The main effect of the belief of exercise having a *negative impact on cancer* was not significant, but decreasers were significantly more concerned about the negative impact of exercise on cancer than increasers (F(1,345) = 4.39, p= 0.037). Perceived benefits of exercise and barriers to exercise were NS.<br><br>The *personal control* subscale of *illness perceptions* significantly differed between exercise groups (F(2345)= 4.11, p=.017, ηp^{2}=.02), with lower *personal control* among decreasers than increasers (F(1, 345) = 5.26, p=.022, d=.28) and no difference with maintainers. The *emotional representations* subscale of *illness perceptions* significantly differed between exercise groups (F(2345)= 3.16, p=.043, ηp^{2}=.02), with decreasers reporting lower *emotional representations* than increasers (F(1345) = 6.01,

					p =0.015, d = 0.27) and no difference with maintainers. *Illness coherence* and *consequences* subscales of *illness perceptions* were NS.	
Craike et al. (2018)[74]<br><br>Australia	Intervention study (RCT)<br><br>12-week exercise program	147 prostate cancer survivors having completed active treatment within the previous 3-12 months (intervention: n= 54, control: n= 93)<br><br>Mean (SD) age 65.6 (8.5) years	*Intra-individual*: Outcome expectations (Multidimensional Outcome Expectations for Exercise Scale), and self efficacy (scale designed by Bandura[15]).	PA was assessed using an adapted version of the Leisure-Time Exercise Questionnaire at baseline, postintervention (12 weeks), and at 6 months.<br><br>Baseline values for PA are not mentioned.	*Intra-individual:* Increases in *barrier self-efficacy* (b= 1.61, 95% CI= 0.57 - 2.65, η2 = 0.08, p = .003) and reductions in *motivation* (b= −21.19, 95% CI = −41.60 - −0.78, η2 = 0.04, p = .04) predicted improved participation in follow-up VPA, controlled for VPA at baseline	Control condition consisted of usual care regarding PA advice.
Driessen et al. (2021)[83]<br><br>The Netherlands	Cross-sectional	229 newly diagnosed cancer patients<br><br>59% *endometrial,* and 41% *ovarian* cancer<br><br>Mean (SD) age 66 (9.5) years<br><br>Median (IQR) BMI 27.7 (24.2 – 32.8) kg/m^2	*Intra-individual*: Health awareness, meaning of cancer, appearance concerns, body change concerns, life interferences, and worry<br><br>All cancer-related psychosocial factors were assessed using the Impact of Cancer Scale version 2.	PA change was assessed using a self-developed questionnaire, in which participants reported whether they made changes in PA since completion of cancer treatment as compared to pre-diagnosis. Participants were classified into groups: no change in PA, more PA and less PA.<br><br>63% reported not having changed PA, 17% reported less PA and 20% reported more PA.	*Intra-individual: Higher health awareness* (OR= 2.79, 95% CI: 1.38, 5.65), *body change concerns* (OR= 3.04, 95% CI: 1.71; 5.39), *life interferences* (OR =4.88, 95% CI: 2.29; 10.38) and *worry* (OR= 2.62, 95%CI: 1.42; 4.85) were associated with less PA as compared to no change in PA. Associations between meaning of cancer and appearance concerns and less PA were NS. Associations between the psychosocial factors and more PA were NS.	
Emery et al. (2009)[29]<br><br>USA	Prospective cohort study	227 women diagnosed with stage II or III regional *breast cancer*<br><br>Mean (SD) age 50.9 (10.8) years	*Intra-individual*: Health-related quality of life (The Medical Outcomes Study-Short Form), and depressive symptoms (Center for Epidemiological Studies – Depression Scale short form)<br><br>*Inter-individual:* Social support (Perceived Social Support from Family and Friends)	PA was assessed using the Seven-day Physical Activity Recall and standard energy expenditure (MET) was used to calculate an index of energy expenditure for the prior week at baseline, every 4 months during the first year and every 6 months during the subsequent 4 years.<br><br>Baseline mean (SD) 16.6 (27.1) METs Increase in weekly PA to 1 year follow-up 31.3 (49.7) METs Decrease in weekly PA at 5 year follow-up 14.3 (+/-22.5) METs	*Intra-individual:* The physical component score of *health-related quality of life* at baseline was NS, while as a time-varying predictor was found to be a significant predictor of change in PA (p=.021). The mental component score of *health-related quality of life* was found to be a significant predictor of the rate of change in PA both when included at baseline (p=.025) and when included as a time-varying predictor (p=.013). *Perceived support* from family at baseline was significantly associated with change in PA (p=.042). *Perceived support* from friends and *depressive symptoms* were NS.	

Eng et al. (2018)[67]<br><br>Canada	Cross-sectional	1003 Cancer survivors having completed active treatment<br><br>16% *breast,* 13% *gastrointestinal*, 10% *genitourinary*, 12% *gynecologic*, 11% *head and neck*, 19% *hematologic*, 6% *lung*, and 11% other<br><br>Median (IQR) age 55 years (19-97) at diagnosis<br><br>47% female	*Intra-individual:*<br>perceptions of PA (quality of life, fatigue, and overall survival; 7-point Likert scales)	PA was retrospectively reported at 1 year before their cancer diagnosis, during treatment and at follow-up using an adapted validated tool by Godin et al.[19] and participants were dichotomized based on whether they met MVPA guidelines at each time point. Participants were divided into two subgroups based on whether they met the guidelines before diagnosis and within each subgroup, patients were further divided into those who continued to meet the guidelines at follow-up and those who did not.<br><br>32% met MVPA guidelines at baseline.<br><br>Of patients who met guidelines at diagnosis, 25% were still meeting guidelines during treatment and 50% met guidelines at follow-up.<br><br>Of patients who did not meet guidelines at baseline, 3% reported meeting guidelines during treatment and 8% met guidelines at follow-up.	*Intra-individual:*<br>For patients not meeting guidelines before diagnosis, perceptions of PA improving quality of life (aOR = 11.09) and overall survival (aOR = 8.52) was significantly associated with increased PA after diagnosis. Associations of the three domains of *perceptions of PA* with maintenance of PA after diagnosis were NS.	
Fallon et al. (2018)[30]<br><br>USA	Prospective observational study	1009 *breast*, *colorectal* and *prostate* cancer survivors enrolled within 12 months of completing active treatment<br><br>Mean (SD) age 61.94 (11.00) years<br><br>Mean (SD) time since treatment 7.84 (3.28) months	*Intra-individual:*<br>Self-efficacy (single item adapted from Stanford Chronic Disease Self-efficacy Scale), and outcome expectations (three items on 5-point Likert scales)	PA was assessed using a 15-item scale from the American Cancer Society's Cancer Prevention Study-3 at baseline and at follow-up (average 13.3 months after baseline) and a metabolic equivalent hours per week score was created.<br><br>Mean (SD) MVPA<br>Baseline: 18.43 (21.02)<br>Follow-up: 18.76 (22.00)	*Intra-individual:*<br>Baseline *self-efficacy* (parent model: b= 0.51, p< .01, alternate model: b= 0.36, p< .001)) was significantly associated with MVPA at follow-up. *Outcome expectations* was NS.	
Frensham et al. (2018)[75]<br><br>Australia	Intervention study<br><br>Quasi-randomized controlled trial	91 cancer patients (intervention: n= 46, control: n= 45) not currently receiving active treatment	*Intra-individual:*<br>Motivation, barriers self-efficacy, and relapse self-efficacy were assessed using the Physical Activity	Participants wore a pedometer for 7 consecutive days to assess daily step count.<br><br>Intervention group: Mean (SD) steps/day	*Intra-individual:*<br>Changes in *relapse self-efficacy* (p<.0001) *and barrier self-efficacy* (p=.001) were significantly associated with changes in steps per day in the	Only patients were included that were insufficiently ative (engaging in less than 20 sessions of exercise over the past month)

	12-week walking intervention	Intervention group: Mean (SD) age 65.2 (9.3) years 54.3% female 45.7% *breast,* 19.6% *prostate*, 10.9% *colorectal*, 6.5% *head and neck*, 2.2% *gynecologic*, 2.2% *lung*, 13.0% other<br><br>Control group: Mean (SD) age 66.1 (9.4) years 48.9% female 37.8% *breast*, 24.4% *prostate*, 8.9% *colorectal*, 6.7% *head and neck*, 4.4% *gynecologic*, 17.8% other	Maintenance Assessment.	Baseline: 7055 (2633) Week 12: 9274 (3579) 3 month follow-up: 8437 (3487)<br><br>Control group: Mean (SD) steps/day Baseline: 6667 (2993) Week 12: 7499 (3320) 3 month follow-up: 7333 (4044)	intervention group. *Motivation* (p=.137) was not significantly associated with changes in steps per day. Only the association between changes in *relapse self-efficacy* and changes in steps per day was significant independent of the other predictor variables (p=.077).	Control condition consisted of an active control group (was provided an pedometer but did not have access to online program)
Golsteijn et al. (2018)[84]<br><br>The Netherlands	Intervention study (RCT)<br><br>3-months computer-tailored PA intervention	478 *prostate* and *colorectal* cancer survivors undergoing treatment or after completing primary treatment up to one year ago (intervention: n= 249, control: n= 229).<br><br>Intervention group: Mean (SD) age 66.55 (7.07) years 14.9% female Mean (SD) time since treatment 5.64 (3.84) months<br><br>Control group: Mean (SD) age 66.38 (8.21) years 10.9% female Mean (SD) time since treatment 5.17 (3.49) months	*Socio-demographic:* Educational level	MVPA was measured using the validated Short Questionnaire to Assess Health Enhancing Physical Activity at baseline, 3 months an 6 months and accelerometers at baseline and 6 months.<br><br>Mean (SD) min MVPA/week Intervention group: *Questionnaires:* Baseline: 798 (721) 3 months: 1060 (771) 6 months: 1145 (883) *Accelerometer:* Baseline: 271 (211) 6 months: 331 (234)<br><br>Control group: *Questionnaires:* Baseline: 873 (764) 3 months: 962 (833) 6 months: 943 (769) *Accelerometer:* Baseline: 293 (229) 6 months: 301 (219)	*Socio-demographic:* The effect of the intervention was moderated by *educational level*, as the intervention resulted in a significant increase in PA in participants with a medium *educational level* (b= 106.85, p=.001, ES=0.59), in a borderline significant increase for highly educated participants (b=56.33, p=.06, ES=0.42) and no increase for those with a low education (b=-0.11, p=.99, ES=0.03).	
Green et al. (2014)[76]<br><br>Australia	Cross-sectional	92 *Prostate* cancer (PC) survivors and 145 *breast* cancer (BC) survivors undergoing treatment or having completed treatments<br><br>BC survivors: Mean (SD) age 56.8 (10.5) years Median (SD) time since diagnosis	*Socio-demographic:* Educational level<br><br>*Intra-individual*: illness representations (timeline acute/chronic, timeline cyclical, consequences, personal control, treatment control, illness coherence and emotional representations) assessed by the Illness Perception	To assess PA changes participants were asked to report the extent to which their behavior had changes since diagnosis on a five point scale.<br><br>41% of BC survivors and 39% of PC survivors reported increasing PA. 33% of BC and 27% of PC reported decreasing PA.	*Intra-individual:* Lower *illness identity* (β= -0.27, p = .003), higher *personal control* (β=0.25, p=.001), higher *treatment control* (β=0.21, p=.014), and higher *stage of change* (β=0.36, p=<.001) were found to be significant predictors of PA change. S*elf-efficacy, timeline acute/chronic, timeline cyclical, consequences, illness coherence and*	

		53.5 (63.11) months<br>Mean (SD) BMI 26.3 (5.4) kg/m^2<br><br>PC survivors:<br>Mean (SD) age 66.6 (7.7) years<br>Median (SD) time since diagnosis 30.0 (45.0) months<br>Mean (SD) BMI 26.1 (3.1) kg/m^2	Questionnaire-Revised, self-efficacy (Exercise Self-Efficacy scale), and exercise stage of change (measured with a scale developed by Marcus et al.[24]).		*emotional representations* were NS.<br><br>*Socio-demographic:*<br>NS.	
Hackshaw-McGeagh et al. (2015)[85]<br><br>UK	Prospective longitudinal observational study	511 men diagnosed with *prostate* cancer<br><br>Mean age 62.3 (range 50 - 70) years<br>33.3% had a healthy BMI with a median (IQR) of 23.5 (22.5 – 24.1) kg/m^2<br><br>66.7% had an unhealthy BMI with a median (IQR) of 28.0 (26.4 – 29.8) kg/m^2	*Socio-demographic*: Age, marital status, and social class	PA was assessed using the Godin and Shephard Leisure-Time Physical Activity Questionnaire at recruitment and at 12 months post-recruitment (9 months post-diagnosis). Participants were dichotomized into 'sufficient to achieve health benefits' and 'insufficient to achieve health benefits'.<br><br>Prior to diagnosis, 334 (65.4%) were sufficiently active of whom 52 (15.9%) decreased their PA at follow-up. Of the 177 men being insufficiently active pre-diagnosis, 72 (40.7%) sufficiently increased their PA.	*Socio-demographic:*<br>Men who were insufficiently active pre-diagnosis and who were of 'working' *occupational class* were 2.03 (p=.04, 95% CI= 1.03 - 3.99) times more likely to increase PA at follow-up compared to managerial and professional men. No significant association was found with *age* and *marital status*.	
Hall et al. (2019)[31]<br><br>USA	Cross-sectional	258 early stage cancer survivors who had completed primary treatment<br><br>27% *breast*, 21% *hematologic*, 11% *gynecologic*, 9% *GI*, 8% *genitourinary*, 6% *head and neck*, 6% *melanoma*, 4% *thoracic* and 2% other<br><br>54% was 60 years or older<br><br>64% female	*Intra-individual*: Fear of cancer recurrence (Assessment of Survivor Concerns), and emotional distress (two items on 5-point Likert scale)	PA change was assessed by asking participants how PA had changed compared to before their diagnosis and responses were categorized in to decreased, no change or improved.<br><br>39% of survivors reported decreasing PA, 18% reported improving PA and 43% reported no change.	*Intra-individual:*<br>Higher *fear of cancer recurrence* (r=-0.18, p<.01) and *emotional distress* (r=-0.23, p<.001) were associated with reduced PA.	
Hardcastle et al. (2021)[77]<br><br>Australia	Intervention study (RCT)<br><br>Behavioral intervention	64 *Colorectal* or *gynecologic* cancer survivors (stages I and II) having completed treatment in the previous five years (34 intervention, 34 control)	*Intra-individual:* *M*aintenance self-efficacy (6 items), action planning (4 items), and intention to participate (2 items).	An accelerometer was used to assess PA (time spent in MVPA in minutes/week) at baseline, 12 weeks and 24 weeks post-intervention. Residualized change scores were computed	Intention (β = 0.213, p = 0.031, ES = 0.040) and action planning (β = 0.202, p = 0.039, ES = 0.058) significantly predicted MVPA. The effect of maintenance self-efficacy (β = 0.162, p = 0.212, ES = 0.025) was not statistically	Participants that were included had cardiovascular disease risk factors and were insufficiently active.

		Mean (SD) age 64.3 (7.7) years		by regressing 24-week follow-up score on 12-week follow-up and baseline scores and extracting the unstandardized residual.	significant. Examination of the total effects of intention on MVPA revealed statistically significant effects ($\beta = 0.237$, $p = 0.019$, ES = 0.044)
Hsu et al. (2011)[94]<br><br>Taiwan	Prospective, longitudinal, repeated measures design	196 women diagnosed with *breast* cancer who completed treatment<br><br>Median time between end of treatment and T1 interview was one month<br><br>Mean (SD) age 47 (9.9) years	*Socio-demographic:* Years of education, and age<br><br>*Intra-individual:* Perceived (physical and mental) health status (SF-12 Health Survey), exercise self-efficacy (self-efficacy scale designed by Bandura[15]), fatigue (fatigue intensity scale), perceived barriers to exercise (Exercise Benefits/Barriers Scale), and exercise outcome expectancy (Outcome Expectation for Exercise Scale)<br><br>*Inter-individual:* Social support (Social Provisions Scale for Exercise)	PA was assessed at baseline, after three months and after six months using a 21-item exercise log and the Borg Scale to indicate perceived exertion with which they had performed the exercise activity. Responses were modified by the leisure score index of the Godin Leisure Time Exercise Questionnaire and 7 Day Physical Activity Recall Interview Questionnaire.<br><br>There were significant mean differences in exercise between baseline and 3 month follow-up ($p<.001$) and between baseline and 6 month follow-up ($p=.004$)	*Mental health* ($\beta=0.88$, $p=.011$), *perceived barriers* ($\beta=0.56$, $p=.044$) and *age* ($\beta=-1.17$, $p=.018$) significantly predicted increased exercise frequency from baseline to 3 months follow-up *Years of education, social support, self-efficacy, fatigue,* and *outcome expectancy* did not significantly predict increased exercise frequency from baseline to 3 months follow-up. Significant interactions were observed between *age* and *social support* ($\beta=1.45$, $p=.016$) and between *mental health* and *perceived exercise barriers* ($\beta=-0.91$, $p=.020$).<br><br>*Social support* ($\beta=-1.11$, $p=.003$), *outcome expectancy* ($\beta=0.16$, $p=.048$), and *age* ($\beta=-1.43$, $p=.005$) were significant predictors of increased exercise from baseline to 6 months follow-up. *Years of education, fatigue, perceived barriers, mental health,* and *self-efficacy* were not significant predictors of increased exercise frequency from baseline to 6 months follow-up. Significant interactions were observed between *age* and *social support* ($\beta=1.95$, $p=.002$) and *fatigue* and exercise history ($\beta=-0.27$, $p=.008$).
Humpel et al. (2007)[78]<br><br>Australia	Cross-sectional	113 cancer survivors<br><br>41.6% *breast*, 14.9% *melanoma*, 10.9% *cervical*, 6.9% *colon/rectal*, 5.9% *ovarian*, and 19.8% other<br><br>81.7% female<br><br>49.6% 55 years or older	*Socio-demographic:* Marital status, employment status, age and educational level	PA was measured using the Active Australia Survey and PA change was assessed by asking participants whether they did more, less or had not changed the amount of PA since diagnosis.<br><br>31.3% reported an increase in PA<br>62.5% remained the same<br>6.3% decreased PA	*Socio-demographic: Marital status , employment status, age,* and *educational level* were not found to be significant predictors of PA change.

		71.2% more than 2 years after diagnosis				
Kindred et al. (2018)[33]<br><br>USA	Intervention study (RCT)<br><br>12-week home-based PA program	46 *Colorectal* cancer (stage 0-3) survivors who completed primary treatment less than 5 years ago<br><br>Mean (SD) age 57.3 (9.73) years<br><br>57% female	*Intra-individual:* Mood (The Profile Mood States questionnaire), self-efficacy for exercise (Exercise Self-efficacy questionnaire), and exercise processes of change (cognitive and behavioral; Exercise Processes of Change)<br><br>*Socio-demographic:* Age, gender, educational level, and employment	Sedentary time (SED) was assessed using an accelerometer at baseline, 3 months, 6 months and 12 months follow-up<br><br>Mean (SD) minutes/monitoring period<br>Baseline: 1641.65 (480.56)<br>12 months: 1791.78 (342.21)<br><br>4 classes of sedentary behavior over time: 22% having high and sustained SED, 24% low and sustained SED, 33% with increasing SED over time and 22% with high SED decreasing from 6 months to 12 months	*Intra-individual: Mood disturbance* was significantly lower at baseline among those with low and sustained SED over time (p<.05). *Cognitive processes* were significantly higher at baseline among participants with increasing SED over time (p<.05). *Self efficacy,* and *behavioral processes* were NS.<br><br>*Socio-demographic: Gender*, and *employment* significantly differed across classes (p<.05). *Age* and *education* were NS.	Control group consisted of a contact control condition<br><br>Participants were only included if they were sedentary (<60 min/week of moderate intensity PA or <20 min/week of vigorous intensity PA)
Kindred et al. (2019)[34]<br><br>USA	Intervention study (RCT)<br><br>12-week telephone counseling intervention	192 *Colorectal* cancer survivors who completed primary treatment less than 5 years ago (intervention: n= 106, control: n= 86)<br><br>Mean (SD) age 55.9 (9.8) years<br><br>Mean (SD) time since diagnosis 2.9 (2.2) years<br><br>Mean (SD) BMI 29.2 (5.7) kg/m^2	*Intra-individual:* Self-efficacy for exercise (Exercise Self-Efficacy questionnaire), exercise processes of change (behavioral and cognitive; Exercise Processes of Change), and decisional balance (scale by Marcus et al.[33])<br><br>*Inter-individual:* Social support (Social Support for Exercise)	PA was assessed using the Seven day physical activity recall at baseline, 3 months, 6 months and 12 months follow-up.<br><br>Control participants mean (SD) min/week:<br>Baseline: 51.78 (69.65)<br>6 months: 80.03 (82.96)<br>12 months: 87.01 (111.58)<br><br>Intervention participants mean (SD) min/week:<br>Baseline: 49.32 (70.72)<br>6 months: 107.62 (110.41)<br>12 months: 87.66 (87.61)	*Self-efficacy* (b=39.96, SE= 9.13, p<.05), *decisional balance pros* (b=23.83, SE= 8.77), *decisional balance cons* (b =-27.22, SE= 10.71), *cognitive* (b=26.59, SE=13.31, p<.05), and *behavioral processes* (b=61.50, SE=11.54, p<.05) were associated with greater PA at 6 months controlling for baseline. *Social support* from family or friends was not significantly associated with PA adoption.<br><br>*Self-efficacy* (b=22.01, SE=8.99, p<.05), *behavioral processes* (b=40.10, SE=13.64, p=<.05), *social support from family* (b=2.48 SE=1.05, p<.05) and *social support from friends* (b=2.19, SE=1.05, p<.05) were associated with greater mean min/week of PA at 12 months controlling for PA at 6 months. *Cognitive processes, decisional balance pros and decisional balance cons* were not significantly associated with PA maintenance.	Control group consisted of contact control<br><br>Participants were only included if they were relatively inactive (<30 min/week of vigorous intensity activity or <90 min/week of moderate intensity activity)

Kwarteng et al. (2020)[35]<br><br>USA	Intervention study (RCT)<br><br>6-month weight loss intervention	246 female overweight African-American *breast* cancer survivors who completed treatment at least 6 months before recruitment (Intervention: n= 125, Control: n= 121)<br><br>Mean (SD) age 57.5 (10.1) years<br><br>Mean (SD) BMI 36.1 (6.2) kg/m^2	*Intra-individual:* PA self-efficacy (11-item instrument by Latimer et al.[35]), perceived access to exercise, and perceived neighborhood safety<br><br>*Inter-individual:* Social support (The Social Support questionnaire)	PA was assessed using the Modified Activity Questionnaire<br><br>Intervention group mean (SD) min/week: Baseline: 162.6 (188.5) 6 month change: 114.2 (248.7)<br><br>Control group mean (SD) min/week: Baseline: 165.5 (226.9) 6 month change: 61.6 (350.2)	*Intra-individual: Self-efficacy* (β=2.07, p= 0.019), higher *perceived access to exercise* (β= 15.329, p= 0.005) were associated with increased minutes per week of PA. Perceived neighborhood safety was NS.<br><br>*Inter-individual: Friend support* for exercise habits-participation (β= 2.613, p= 0.049) were associated with increased minutes per week of PA. Support from family was NS.	Only overweight/obese survivors were included (BMI ≥ 25 kg/ m2)<br><br>Control condition was a Self-Guided Group
Kwarteng et al. (2021)[36]<br><br>USA	Intervention study (RCT)<br><br>6-month weight loss intervention	246 female overweight African-American *breast* cancer survivors at least 6 months post-treatment (Intervention: n=125, Control: n=121)<br><br>Mean (SD) age 57.5 (10.1) years	*Intra-individual:* Life stress (Crisis in Family systems)	PA was assessed using the Modified activity questionnaire at baseline, post-intervention (6 months) and maintenance (12 months)<br><br>Intervention group mean (SD) min/week: Baseline: 162.6 (188.5) 6 month change: 114.2 (248.7) 12 month change: 108.7 (275.7)<br><br>Control group mean (SD) min/week: Baseline: 165.5 (226.9) 6 months change: 61.6 (350.2) 12 month change: 71.0 (274.2)	*Intra-individual: Life stress* was not significantly associated with change in PA in the intervention or control group during the intervention phase. Similarly, during the maintenance phase, *life stress* was not significantly associated with change in PA in the intervention or control group.	Only overweight/obese survivors were included (BMI ≥ 25 kg/ m2)<br><br>Control condition was a Self-Guided Group
Lee et al. (2016)[68]<br><br>Canada	Single-group longitudinal study	42 women diagnosed with stage 0-III *breast* cancer who completed primary treatment at least one month prior to the intervention<br><br>Mean (SD) age 53.8 (9.2) years<br><br>Mean (SD) time since diagnosis 24.4 (29.6) months<br><br>Mean (SD) BMI 31.2 (6.3) kg/m^2	*Intra-individual:* PA self-efficacy (Physical activity Self-efficacy scale), and fatigue (Functional Assessment of Chronic Illness Therapy-Fatigue scale)	PA was assessed at baseline and 6 months postintervention using the interviewer-based Seven Day Physical Activity Recall.<br><br>Mean (SD) kcal/week Baseline: 894.2 (633.2) postintervention: 1169.8 (737) PA maintenance: 1002.8 (664.7)	*Intra-individual:* Baseline fatigue (β = 0.32, p= 0.029) was a significant predictor of PA maintenance. PA self-efficacy was not significantly associated with PA maintenance.	
Lee et al. (2020)[96]<br><br>South Korea	Cross-sectional	251 *colorectal* cancer patients who underwent primary surgery and were receiving or had completed other therapies and was	*Intra-individual*: Self-leadership (behavior awareness and volition, task motivation, and constructive cognition; The Abbreviated Self-Leadership	PA was measured based on 7-day exercise diaries in minutes per week of at least moderate exercise. PA maintenance was	*Intra-individual:* Maintenance of moderate exercise during 6 months had significant correlations with higher *self-leadership* in the following subscales: behavior awareness and	

		diagnosed within the 2 previous years<br><br>Mean (SD) age 62.7 (10.9) years<br><br>30.7% female	Questionnaire), and planning (action and coping planning).	measured in persistence of the exercise during 6 months.<br><br>35.1% maintained more than 150 min of moderate exercise per week for more than 6 months.	volition (aOR: 1.56, 95% CI: 1.16-2.11), task motivation (aOR: 1.51, 95% CI: 1.12–2.04), and constructive cognition (aOR: 1.41, 95% CI: 1.05–1.89).<br>Greater *action planning* was associated with maintenance of exercise for more than 6 months (aOR: 2.07, 95% CI: 1.20–3.59). *Coping planning* was not associated with PA maintenance.	
Loprinzi et al. (2012)[37]<br><br>USA	Prospective, three-armed randomized controlled trial.<br><br>12-month supervised exercise program	69 *breast* cancer survivors older than 65 who had completed primary treatment more than 2 years prior and who were inactive at recruitment<br><br>Mean (SD) age 70.6 (1.2) years<br><br>Mean (SD) time since diagnosis 80.6 (5.4) months	*Intra-individual:* Processes of change (behavioral and cognitive: 30-item measure), self-efficacy (18-item measure), and decisional balance (10-item measure).	PA was assessed using the Community Healthy Activities Model Program for Seniors questionnaire at the conclusion of the exercise program (baseline) and 6 months later.	*Intra-individual:*<br>Participants with higher *self-efficacy* at baseline had greater odds of being sufficiently active at follow-up (OR= 1.10, 95% CI: 1.01-1.18), controlled for PA at baseline.<br>Participants utilizing more *behavioral processes* at baseline had greater odds of being sufficiently active at follow-up (OR: 1.13, 95% CI: 1.02-1.26), controlled for PA at baseline.<br><br>Cons for exercise NS<br>Cognitive niet onderzocht	Participants included were older than 65 and were inactive at recruitment (less than 30 minutes of moderate intensity exercise 3 days a week)
Mama et al. (2015)[38]<br><br>USA and Puerto Rico	Intervention study (RCT)<br><br>16-week culturally adapted exercise intervention	89 sedentary women diagnosed with *breast* cancer stages I-IV who were at least 3 months post-treatment (Intervention: 30, standard program: 29, control group: 30)<br><br>Mean (SD) age 58.5 (9.2) years	*Intra-individual:* Exercise self-efficacy (questionnaire developed by research team), and barriers self-efficacy (the cancer survivors' barriers self-efficacy scale)<br><br>*Inter-individual:* social modeling (8 items previously developed), and social support (Social Support for Exercise Survey)	PA was assessed using the International Physical Activity Questionnaire short form at baseline and 16-week follow-up. Sedentary behavior was measured using a modified version of the Past0day Adults' Sedentary Time Questionnaire<br><br>Across groups, there was a significant effect of time for moderate-intensity (p=.007), vigorous-intensity (p= 0.013), and total (p=0.003) physical activity from baseline to follow-up.	Increased *social support* from family was associated with increases in vigorous-intensity PA (p<.05). Increases in *social modeling* were associated with increases in moderate and total PA and with decreases in sedentary time from baseline to follow-up. Changes in *exercise self-efficacy* were significantly associated with changes in walking (b=62.848, SE=31.390, p=.046) after adjusting for age, site and treatment group. Treatment group moderated the effects of *barrier self-efficacy* on walking (b=41.978, SE=21.129, p=.052) and sitting time (b=727.443, SE=371.008, p=.051) and the effects of *social support* from family on sitting time (b=-90.736, SE=38.82, p=.013)	Control condition consisted of a non-culturally adapted standard exercise intervention or a wait-list control group<br><br>Participants were included if they were not meeting PA guidelines.
Morey et al. (2015)[39]<br><br>USA	Intervention study (RCT)<br><br>12-month Home-based lifestyle	641 survivors of *breast, prostate* and *colorectal* cancer 5 years or more after diagnosis	*Intra-individual:* Self-efficacy<br><br>*Socio-demographic*: Age, gender	PA was assessed at baseline and then every 3 months for 24 months (9 measurement occasions) using 3 items of the	*Intra-individual:*<br>*Self-efficacy* for strength training (p<.0001) and for aerobic exercise (p=.0001) differed between the trajectory groups in the	Control condition consisted of a wait-listed control arm who received the intervention during months 12 to 24

	counseling intervention	(intervention: n= 319, control: n= 322)<br><br>Mean (SD) age 73.1 (5.1) years<br><br>Mean (SD) time since diagnosis 8.6 (2.7) years<br><br>60% was overweight (BMI 25.0-29.9 kg/m2) and 40% was obese (BMI > 30 kg/m2)	Self-efficacy was assessed by a single item for confidence in ability to perform strength training for more than 15 minutes on 3 days per week	Community Health Activities Model Program for Seniors questionnaire<br><br>Three group trajectories were identified for weekly min of PA from the intervention group: 7.1% was and remained inactive, 32.7% increased PA immediately and steadily declined throughout observation period, 60.2% increased PA at 3 months and maintained throughout the intervention period and observation period.<br><br>Three groups were also identified from the waitlist group: groups 1 and 2 remained inactive during the wait-list period, and during intervention period group 1 (8.9%) did not engage in PA, group 2 (24.2) increased PA and remained active. Group 3 was more physically active at baseline and increased and sustained PA throughout intervention.	intervention group, with lower *self-efficacy* more prevalent in the PA trajectory group reporting the lowest amount of PA over time. *Self-efficacy* for strength training (p<.0001) and for aerobic exercise (p<.0001) also differed between the trajectory groups of the waitlist group, with lower self-efficacy more prevalent among the lowest PA group.<br><br>*Socio-demographic:* *Age* differed significantly between trajectory groups of (p=.04) of the waitlist group. *Gender* was NS.	Participants were included if they were not meeting PA guidelines (150 min/week of moderate to vigorous PA)
Ott et al. (2004)[40]<br><br>USA	Exploratory one-group intervention design<br><br>Home-based strength/weight training exercise intervention	23 Postmenopausal *breast* cancer survivors (stage I or II) who completed primary treatment 6 months prior and were 40-65 years of age<br><br>Mean (SD) age 54.8 (7.2) years<br><br>Mean (SD) time since completion of treatment 4.1 (3.0) years<br><br>Mean (SD) BMI 27.0 (4.6) kg/m2	*Intra-individual:* Self efficacy (instrument developed by Marcus et al.[43]), change processes (Modified version of instrument of Marcus et al.[24]), and decisional balance (modified version of 16-item instrument developed by Marcus et al.[33])	A self-developed adherence journal was used to document exercise performance, such as repetitions, sets and amount of weights used.<br><br>Mean (SD) pounds free weight lifted:<br>Arm exercises:<br>Baseline: 3.74 (3.57)<br>6 months: 9.83 (3.58)<br><br>Leg exercises:<br>Baseline: 4.65 (2.55)<br>6 months: 12.48 (4.47)	*Intra-individual:* There was a significant positive relationship between frequency *of use of behavioral processes of change* and increase in pounds lifted for the leg exercises (r=.418, p=.047), but not for the arm exercises. There was no significant relationship between *self-efficacy*, *pros*, and *cons* with increase in pounds lifted.	Participants were excluded if they performed strength/weight training exercises at inclusion
Ottenbacher et al. (2013)[41]<br><br>USA	Intervention study (RCT)<br><br>10-month diet and exercise trial	452 early-stage *breast* and *prostate* cancer survivors who were diagnosed in the past 9 months<br><br>BC survivors:	*Intra-individual:* Cancer-specific concerns (Functional Assessment of Cancer Therapy-Breast or -Prostate)	PA was assessed using the 7-day Physical Activity Recall at baseline and at 1 year follow-up	*Intra-individual:* In prostate cancer survivors, no significant associations were observed between change in *cancer-specific concerns* and change in PA. There was a significant association	Participants were included if they practiced less than 2 health behaviors: exercised at least 150 min/week, adhered to a low-fat diet or consumed five or more

		Mean (SD) age 53.5 (11.4) years<br><br>PC survivors: Mean (SD) age 62.0 (8.3) years			between the baseline *cancer-specific concern* of 'activities limited by urination' and minutes of PA at 1 year, after controlling for baseline activity (p=.02).<br>In breast cancer survivors, there was no significant association between baseline *cancer-specific concerns* and PA at 1 year (controlling for baseline activity). No association was observed between change in PA and change in *cancer-specific concerns* from baseline to 1 year.	daily servings of fruit and vegetables.
Rogers et al. (2017)[42]<br><br>USA	Intervention study (RCT)<br><br>3-month PA behavior change intervention	222 post-treatment *breast* cancer survivors<br><br>Mean (SD) age 54.4 (8.5) years<br><br>Mean (SD) time since diagnosis 54.0 (54.4) months	*Intra-individual:* Task self-efficacy (a walking task self-efficacy scale by Rogers et al.[46]), outcome expectancies (17 items listing 14 potential exercise benefits), and perceived barriers (perceived barrier inference scale)	PA was measured by 7-day worn accelerometer monitoring at baseline, 3 months and 6 months.	*Intra-individual:*<br>There was a borderline significant effect of reductions in *barriers* being associated with greater physical activity at 6 months (β = −0.24, p = .05). The tests of the indirect effects of the intervention on physical activity at 6 months through social cognitive variables were all non-significant. The indirect effect of BEAT Cancer on physical activity at 6 months through reductions in *barriers* was the strongest indirect effect (β =0.05, p =.11).	Control condition consisted of usual care
Rogers et al. (2011)[43]	Intervention study (RCT)<br><br>3-month intervention	41 women diagnosed with stage I-III breast cancer (intervention: 21, control: 20)<br><br>Mean (SD) age 53 (9) years	*Intra-individual:*<br>Barriers self-efficacy (9-item scale specifically designed for assessing barriers self-efficacy in breast cancer survivors), task self-efficacy (4-item scale by Rogers et al.[46]), barrier inference (participants rating on a 5-point scale how often barriers interfered with exercise), outcome expectations, fear of exercise (single item), PA enjoyment<br><br>*Inter-individual: Exercise role models (three items), social support (reported frequency with which friends and family offered to exercise with them or encouraged to exercise)*	PA was measured by 7-day worn accelerometer monitoring at baseline, 3 months and 6 months<br><br>Mean weekly min of PA:<br>Usual care:<br>Baseline: 113.4<br>3 months: 116.5<br>6 months: 92<br><br>Intervention:<br>Baseline:96.2<br>3 months: 165.8<br>6 months: 174.9	*Intra-individual:*<br>*Barrier inference (p=.004) and barriers self-efficacy (p=.02) were significant mediators of intervention effect on PA.*<br>The other variables were NS.<br><br>*Inter-individual:*<br>NS.	Control condition consisted of usual care<br><br>Participants engaging in more than 150 min of moderate plus vigorous PA or more than 60 min of vigorous PA per week were excluded

Shi et al. (2020)[44]	Prospective cohort study	3000 newly diagnosed female *breast* cancer patients<br><br>Mean age at diagnosis 59 (range 26 – 94) years<br><br>Mean time since diagnosis 1.8 (range 0 – 8) months	*Intra-individual:* Depressive symptoms (Center for Epidemiological Studies Depression Scale), and dispositional optimism (Life Orientation Test)<br><br>*Inter-individual*: Social support (Medical Outcome Study Social Support Survey Instrument)<br><br>*Socio-demographic*: Education, income, and age	PA was assessed using the Arizona Activity Frequency Questionnaire. Baseline PA was collected during an in-person interview, and at 6 and 24 months follow-up PA was assessed via questionnaires.<br><br>Three latent trajectories were identified for MVPA: high decreasers (7%), medium decreasers (35%) and low maintainers (58%)<br><br>Four latent trajectories were identified for sedentary behavior: high maintainers (18%), high decreasers (27%), low increasers (24%) and low maintainers (31%)	Participants with higher *education* [OR ranged between 1.63 (95% CI: 1.16–2.30) to 4.37 (95% CI: 1.84– 10.40)], were more *optimistic* [OR ranged between 1.60 (95% CI: 1.28– 2.00) to 1.86 (95%CI: 1.26–2.76)], and reported higher *social support* (OR= 1.33, 95% CI: 1.06–1.67) were more likely to be high decreasers and medium decreasers compared to low maintainers (all P < 0.05). However, these associations were not significant after adjusting for baseline MVPA.<br><br>For sedentary behavior, participants with higher *education* (OR = 1.84, 95% CI: 1.15–2.94), and higher *perceived social suppor*t [OR ranged between 1.42 (95% CI: 1.08–1.87) to 1.86 (95% CI: 1.34–2.57)] were more likely to be high maintainers or high decreasers of SB, compared to the low maintainers (all P<0.05). *Higher education* remained significant with being high maintainers vs. low maintainers after adjusting for baseline sedentary behavior
Steinhilper et al.(2013)[80]<br><br>Germany	Longitudinal prospective study	229 female *breast* cancer survivors<br><br>Mean (SD) age 53.2 (9.5) years<br><br>Mean (SD) time since surgery at follow-up 14 (3.6) months	*Socio-demographic:* Educational level, marital status, job position, employment status, and age	PA was assessed with an interview conducted immediately after surgery (baseline) and 14 months follow-up with the questions being extracted from different instruments. Total exercise (MVPA) was dichotomized in meeting or not meeting the recommendations (180 min of weekly PA).<br><br>At follow-up, 46.1% increased PA, 25.4% decreased PA and 28.5% reported no change.<br><br>Mean (SD) hours/week PA:<br>Baseline: 2.2 (2.7)<br>Follow-up: 2.9 (2.5)	*Socio-demographic:* Participants in *higher occupational positions* showed significantly less improvement over time in moderate PA. *Age, employment status, educational level* and *marital status* did not significantly predict changes in PA.

				12% increase in women meeting recommendations		
Ungar et al. (2016)[81]<br><br>Germany	Intervention study<br><br>4-week Health Action Process Approach-based intervention	67 cancer survivors currently receiving out-patient therapy or having finished therapy not longer than 6 months ago (intervention: n= 35, control: n= 32)<br><br>Intervention group: Mean (SD) age 56.69 (13.43) years 54.3% female<br><br>Control group: Mean (SD) age 54.09 (11.72) years 50% female	*Inter-individual:* Role model<br><br>Participants in the intervention group got matched with a same-sex role model as exercise partner. 23 could be matched as the others lived too far away. Therefore 22 were having a role model and 13 were without role model.	PA was assessed using the self-reported Short Questionnaire to Assess Health-enhancing PA at baseline (T1), 4 weeks after the intervention (T2) and 10 weeks after T2 (T3) and an accelerometer at T1 and T2.<br><br>A significant main effect of time ($F(2,130) = 9.35$, $p < .001$, $\eta_p^2 = .126$) indicated that both groups increased their exercise level. The accelerometer data showed a similar (nonsignificant) pattern from T1 to T2 for the exercise group (M= 64 min/week to M= 81 min/week), but no change (M= 68 min/week at both time points) in the stress management group ($F(1,39) = 0.31$, $p = .310$, $\eta_p^2 = .041$).	*Inter-individual:* Comparing exercise patients with a *role model* to exercise patients without a *role model* and control patients showed a significant main effect for condition ($F(2,64) = 3.76$; $p = .028$; $\eta_p^2 = .105$). Those in the intervention group that had contact with a *role model*, exercise levels significantly increased from a mean of 51.82 at T1 to 260.45 at T2 minutes/week of PA, compared to those without any contact, who fell almost back to baseline at T3.	Control condition consisted of a stress management intervention (active control)<br><br>Participants were included if they did not meet the current PA guidelines (150 min/week of PA)
Ungar et al. (2016)[82]<br><br>Germany	Intervention study (RCT)<br><br>4-week exercise intervention	67 cancer survivors currently receiving out-patient therapy or having finished therapy not longer than 6 months ago (intervention: n= 35, control: n= 32)<br><br>32.8% breast, 11.9% colorectal, 7.5% prostate, and 43.5% other<br><br>52% female<br><br>Mean (SD) age 55.45 (12.62) years	*Intra-individual:* PA enjoyment (2 items based on Rogers et al.[47]), and maintenance self-efficacy (according to guidelines of the Health Action Process Approach, Schwarzer et al.[52]),	Pa was measures using a modified Short Questionnaire to Assess Health-enhancing physical activity at baseline, post-intervention, and at 10-week follow-up<br><br>Mean (SD) minutes intended PA<br>*Intervention:*<br>Baseline: 52.14 (101.95)<br>Post-intervention: 150.43 (161.13)<br>10 week follow-up: 189.43 (243.14)<br><br>*Control:*<br>Baseline: 41.84 (92.47)<br>Post-intervention: 69.06 (121.10)<br>10 week follow-up: 120.00 (193.38)	*Intra-individual:*<br>Self efficacy at baseline ($\beta = .35$, $p < .01$) and change in self-efficacy ($\beta = .29$, $p < .05$) were positive significant predictors of PA change at post-intervention. Change in PA enjoyment significantly predicted PA at post-intervention over and above self-efficacy ($\beta = 0.32$, $p < .05$).<br><br>Self-efficacy at baseline ($\beta = .46$, $p < .01$), but not change in self-efficacy, predicted change in PA at 10-week follow-up. PA enjoyment was not a significant predictor over and above self-efficacy.	Control condition consisted of a stress management intervention<br><br>Participants were included if they did not meet the current PA guidelines (150 min/week of PA)
Valle et al. (2015)[45]<br><br>USA	Intervention study (RCT)<br><br>12-week Facebook-based intervention	86 young adult cancer survivors between the ages 21-39 more than 1 year beyond diagnosis date and having completed treatment	*Intra-individual:* Self-efficacy (Self-efficacy and Exercise Habits Survey)<br><br>*Inter-individual:* Social support (adapted from	PA was assessed using a modified version of the Godin Leisure-Time Exercise Questionnaire at baseline and after 12 weeks	*Intra-individual:*<br>NS.<br><br>*Inter-individual:*<br>Changes in *social support* from friends on social networking website was positively related to change	Control group consisted of Facebook-based self-help<br><br>Participants were included if they exercised less than 150

		Intervention:<br>Mean (SD) age 30.8 (5.7) years<br>91.1% female<br>Mean (SD) BMI 28.4 (8.2) kg/m2<br><br>Control:<br>Mean (SD) age 32.7 (4.2) years<br>90.2% female<br>Mean (SD) BMI 29.1 (8.9) kg/m2	the Social Support and Exercise Survey)	Intervention mean (SD):<br>Baseline: 109.3 (125.0) MVPA<br><br>Control mean (SD)<br>Baseline: 118.4 (126.3) MVPA<br><br>No significant effect of intervention on change in MVPA (b=11.89, SE=30.81).	in MVPA (b= 13.61, p= 0.0006). Across both groups, changes in MVPA were predicted by changes in total *social support* (b= 6.21, p= 0.0003) and *social support* from friends (b= 10.35, p= 0.006). Both changes in mild PA (b= - 27.69, p= 0.001) and changes in total PA (b=-22.41, p = 0.046) were negatively associated with changes in family *social support.*	min of MVPA per week
Vallerand et al. (2019)[69]<br><br>Canada	Intervention study (RCT)<br><br>12-week theory-based telephone counseling exercise intervention	51 *hematologic* cancer survivors (intervention: n= 26, control: n= 25)	*Intra-individual:* Motivational processes (instrumental attitudes, affective attitudes, perceived capability and perceived opportunity), behavioral regulations (exercise action and coping plans, and social support), and reflexive processes (anticipated regret, habit, exercise identity, exercise obligation, and regulation of alternatives)	MVPA was assessed by a modified version of the Leisure Score Index from the Godin Leisure-Time Exercise Questionnaire at baseline and post-intervention (week 13).<br><br>Mean change MVPA<br>Intervention: 218 minutes<br>Control: 93 minutes	*Intra-individual:* Potentially meaningful mediators of intervention effect on PA behavior were *coping planning*, (b = 24.98, β = 0.18, 95% CI, −0.03 - 0.56), *exercise identity* (b = 17.43, β = 0.12, 95% CI, −0.05 - 0.41), *habit* (b = 14.64, β = 0.10, 95% CI, −0.01 - 0.42), *obligation* (b = 12.25, β = 0.09, 95% CI, −0.11 - 0.34), *perceived opportunity* (b = 17.95, β = 0.13, 95% CI, 0.01 - 0.36), *affective attitude* (b = 12.85, β = 0.09, 95% CI, −0.01 - 0.30), and *perceived capability* (b = 10.52, β = 0.07, 95% CI, −0.02 - 0.28). However, the only statistically significant mediator was *perceived opportunity*.	Control condition consisted of self-directed exercise for 12 weeks<br><br>Participants reported more than 240 minutes of weekly aerobic exercise were excluded
Webb et al. (2019)[86]<br><br>UK	Intervention study (RCT)<br><br>12-week print-based intervention supported by internet tools	207 adult cancer survivors (intervention: n = 104, control: n=103)<br><br>38.2% *breast*, 13.0% *colorectal*, 6.8% *prostate*, and 42.0% other<br><br>73.9% female<br><br>61.8% between the ages 45-64<br><br>77.3% within 5 years of diagnosis	*Intra-individual:* Self-efficacy (single-item measure based on a measure developed by Marcus et al.[43])	PA was assessed using the Godin Leisure-Time Exercise Questionnaire at baseline, at 12 weeks and maintenance was assessed at 24 weeks. Participants were categorized into sufficiently active, moderately active and active.<br><br>Mean (SD) PA<br>Intervention:<br>Baseline: 25.99 (19.37)<br>12 weeks: 35.57 (23.71)<br>24 weeks: 40.84 (34.85)<br><br>Control:<br>Baseline: 28.70 (24.05)<br>12 weeks: 31.31 (22.65)<br>24 weeks: 39.49 (28.97)	*Intra-individual:* *Self-efficacy* was not a significant predictor of PA improvement in the intervention arm (β = - 1.33, P = 0.36, R^2 = 0.008)	Control condition consisted of a wait list control group

Wilkinson et al. (2012)[46]<br><br>USA	Intervention study (RCT)<br><br>1-year tailored intervention	452 *breast* and *prostate* cancer survivors within 9 months of diagnosis (intervention: n = 224, control: n = 228)<br><br>53.7% female<br><br>Mean (SD) age at diagnosis 57.9 (10.6) years<br><br>37.7% was overweight (BMI 25.0 -29.9 kg/m2) 24.8% was obese (BMI >30.0 kg/m2)	*Intra-individual:*<br>Cancer coping style (15-item version of Mini-MAC to classify participants as fighting-spirits, fatalist or other)	PA was assessed using the 7-day Physical Activity Recall at baseline, 1 year follow-up (completion of intervention) and 2 year follow-up (observational period)<br><br>Significant main effect of time, PA changed over time in both intervention and control group (p<.05)	*Intra-individual:*<br>The interaction between time and *coping style* was not significant for either group.	Participants were excluded if they practiced at least two of the following behaviors: consumption of more than 5 daily servings of fruit and vegetables, adherence to a diet less than 30% of energy from fat, participation in more than 150 min of exercise per week.<br><br>Control condition consisted of standardized intervention
