# Supplementary material for: Psychosocial Determinants of Lifestyle Change after a Cancer Diagnosis: A Systematic Review of the Literature
Source: Cancers (Basel). 2022 Apr 16;14(8):2026. doi: 10.3390/cancers14082026 (PMC9032592; doi:10.3390/cancers14082026)
Supplement: Supplementary file 1 [file cancers-14-02026-s001.zip › Table S2_MH.pdf]

Table S2. Overview of quantitative studies assessing psychosocial determinants of changes in diet among cancer survivors (n=21).

First author (year) Country	Study design	Sample Characteristics *(at baseline)*	Psychosocial variables *Type(s); Assessed at; Assessed with*	Lifestyle *Assessment instruments Baseline; Change (period)*	Findings	Comments
Alfano et al. (2009)[26]<br><br>USA	Cross-sectional descriptive study	227 female long-term *breast cancer* survivors<br><br>Mean (SD) age 61.9 (9.9) years<br><br>Mean (SD) time since diagnosis 12.4 (1.8) years	*Inter-individual:* Social support (MOS Social Support Survey)<br><br>*Intra-individual:* Depressive symptoms (Center for Epidemiologic studies – Depression Scale); fear of recurrence (modified form of the Breast Cancer Anxiety and Screening Behavior Scale); fatigue (vitality subscale of the Medical Outcomes Study Short Form-36); stressful life events (11-item Life Events Scale); satisfaction with sexual functioning (Watts Sexual Functioning Questionnaire); body satisfaction (Self-Concept Scale)	Changes in dietary intake were measured by asking the participants whether their fat intake, fiber intake, and fruit and vegetable intake increased or decreased following diagnosis.<br><br>No baseline assessment of dietary intake reported.<br><br>*Fat intake*<br>Decreased: n=100 (44%)<br>Increased or no change: n=127 (56%)<br><br>*Fiber intake*<br>Increased: n=96 (42%)<br>Decreased or no change: n=131 (58%)<br><br>*Fruit and vegetable intake*<br>Increased: n=98 (43%)<br>Decreased or no change: n=129 (57%)	*Inter-individual:* NS<br><br>*Intra-individual:* There was a trend toward less *fatigue* (e.g. greater vitality scores) among women who reported increasing their fruit and vegetable intake, compared with women who reported decreasing or maintaining their pre-diagnosis dietary patterns (p=0.08).<br><br>All other variables were NS	
Barbosa et al. (2019)[87]<br><br>Portugal	Prospective cohort study	428 women newly diagnosed with *breast cancer* and proposed for surgery<br><br>50.7% had less than 55 years of age at baseline	*Socio-demographic:* Educational level, age, and marital status<br><br>*Intra-individual:* Anxiety and depression (Hospital Anxiety and Depression Scale) at baseline<br><br>Assessments before diagnosis & 3 years after diagnosis	Both pre-diagnosis and post-diagnosis fruits and/or vegetables intake was measured using an interview at 3-year follow-up.<br><br>Participants were classified according to adherence to the recommendation for cancer prevention on fruit and vegetable intake<br>intake (at least five portions of fruits and/or vegetables per day)<br><br>Non-adherence:	*Socio-demographic:* All NS<br><br>*Intra-individual:* NS	

				Pre-diagnosis 69.2%<br>3-year follow-up: 65.6%	
Bergengren et al. (2020)[89]<br><br>Sweden	Cross-sectional nation-wide, population based study	1288 low-risk *prostate cancer* survivors<br><br>Mean age 63 years at diagnosis (Range 59-66)	*Intra-individual:* Health related quality of life (Expanded Prostate Cancer Index Composite 26 (EPIC-26))	Dietary changes were assessed using a questionnaire composed of study-specific questions, by asking whether their food intake changed after receiving the diagnosis. Changes were categorized into: "I eat less healthy", "Unchanged" or "I eat healthier"<br><br>Healthier diet: 184 (14%)<br>Unhealthier diet: 2 (0.2%)<br>Unchanged: 958 (74.4%)	*Intra-individual:* NS
Clotas et al. (2021)[90]<br><br>Spain	Results of the first cross-sectional study of a larger mixed cohort study (retrospective-prospective)	2235 women diagnosed with breast cancer	*Socio-demographic:*<br><br>Age:<br>< 50 years, 50–65 years, and > 65 years<br><br>Social class: based on the occupation of the main provider in the household classified as: nonmanual class (the most privileged class), and manual class (the less privileged class.<br><br>Cohabitation: living alone vs. cohabiting<br><br>40.1% > 65 years<br>75.5% non-manual class<br>22.68% lived alone	Dietary changes were assessed using a food frequency questionnaire. Changes were calculated by comparing eating habits before and after cancer diagnosis.<br><br>Statistically significant change in the consumption of all food groups after diagnosis (p < 0.05). 5.5% had followed a healthy diet before diagnosis, increasing to 9.6% after diagnosis. Positive change for most food groups, especially for consumption of nuts (13.2%), fruit (9.7%), and vegetables (8.8%). However, the percentage of women with unhealthy consumption of eggs (8.5%), dairy products (8.2%), and cereals (7.9%) increased after diagnosis.	*Socio-demographic:*<br><br>Age:<br>Significant association between women's age for the four food groups (p ≤ 0.05), with *younger* women (aged <50 years) showing a greater increase in healthy food types.<br><br>Social class:<br>Significant association between social class for the four food groups (p ≤ 0.05), with those in *nonmanual classes (high classes)* showing a greater increase in healthy food types.<br><br>Cohabitation:<br>Improved pattern of nut consumption in 25.1% of women living alone, compared to 19.6% of those living with one or more people. *Living alone* was also associated with a greater improvement in the consumption of vegetables.
Driessen et al. (2021)[83]	Cross-sectional	229 newly diagnosed cancer patients	*Intra-individual*: Health awareness, meaning of cancer,	By use of self-developed questionnaires,	*Intra-individual:* All NS

The Netherlands		59% *endometrial,* and 41% *ovarian* cancer<br><br>Mean (SD) age 66 (9.5) years<br><br>Median (IQR) BMI 27.7 (24.2 – 32.8) kg/m^2	appearance concerns, body change concerns, life interferences, and worry<br><br>All cancer-related psychosocial factors were assessed using the Impact of Cancer Scale version 2.	participants reported if they made changes (yes/no) in diet since they were diagnosed with cancer until 18 months after initial treatment. Changes were classified as healthy (more consumption of fruit, vegetables, fibers/whole grain and less consumption of fat, meat, sugar, alcohol and salt) or unhealthy (vice versa) according to the World Cancer Research Fund recommendations.<br><br>20% reported to eat healthier.<br>80% reported not having changed their diet (N = 169) or made unhealthier changes (N = 3).	
Green et al. (2014)[76]<br><br>Australia	Cross-sectional	92 *Prostate* cancer (PC) survivors and 145 *breast* cancer (BC) survivors undergoing treatment or having completed treatments<br><br>BC survivors:<br>Mean (SD) age 56.8 (10.5) years<br>Median (SD) time since diagnosis 53.5 (63.11) months<br>Mean (SD) BMI 26.3 (5.4) kg/m^2<br><br>PC survivors:<br>Mean (SD) age 66.6 (7.7) years<br>Median (SD) time since diagnosis 30.0 (45.0) months<br>Mean (SD) BMI 26.1 (3.1) kg/m^2	*Socio-demographic:* Educational level<br><br>*Intra-individual*: illness representations (timeline acute/chronic, timeline cyclical, consequences, personal control, treatment control, illness coherence and emotional representations) assessed by the Illness Perception Questionnaire-Revised, self-efficacy (Exercise Self-Efficacy scale& Nutrition Self-Efficacy Scale); and exercise stage of change (measured with a scale developed by Marcus et al., 1992) and adapted version for diet.	Participants were asked to record the extent to which their diet and exercise behaviour had changed since diagnosis on a five point scale, with three representing have not changed.<br><br>Regarding diet changes since diagnosis, 59% of BC and 59% of PC participants reported eating more healthily, 6% of BC and no PC participants indicated eating less healthily, and 34% and 41%, respectively, remained unchanged.	*Socio-demographic:* Educational level NS<br><br>*Intra-individual:* Healthier eating since diagnosis showed significant independent associations with *higher personal control*; *higher negative emotional representations*; and *higher stage of change* (all p<0.05).
Hagen et al. (2018)[91]<br><br>Norway	Cross-sectional	180 postmenopausal breast cancer survivors	*Socio-demographic:* Age, marital status, years of education.	Patients were asked whether they had made any changes in their diet two years	*Socio-demographic:* Patients who changed their diet were significantly *younger*,

		Median age 58 years (Range 37–78)<br><br>BMI<br><25 kg/m2: 51%<br>>=25: 49%<br><br>Marital status<br>73%married/partnered		after they were diagnosed with breast cancer. If the answer was "yes", they were asked about the major changes they had made in relation to food items excluded or included in their diet. Dietary data was collected using a 36 food-item frequency questionnaire.<br><br>Forty patients (22%) had changed their diet two years after the breast cancer diagnosis, which was largely similar to the controls (315 female blood donors).<br>17 (42%) had increased intake of fruit and vegetables; 13 (32%) water; and 5 (12%) fish. Food items most commonly avoided were sugar (n=21, 52%), and meat (n=10, 25%). Eight patients (20%) had reduced their alcohol consumption.	compared to those who maintained their diet.<br><br>Marital status and educational level both NS.	
Hall et al. (2019)[31]<br><br>USA	Cross-sectional	258 early stage cancer survivors who had completed primary treatment<br><br>27% breast, 21% hematologic, 11% gynecologic, 9% gastrointestinal, 8% genitourinary, 6% head and neck, 6% melanoma, 4% thoracic and 2% other<br><br>54% was 60 years or older<br><br>64% female	*Intra-individual*: Fear of cancer recurrence (Assessment of Survivor Concerns)	Respondents were asked how each health behavior had changed compared with before their diagnosis of cancer. Responses were coded as follows: decreased or worsened, no change, or increased or improved. In addition, each health behavior was assessed by adapting screening questions from the 2016 NCCN health behavior guidelines.<br><br>36% improved diet after cancer diagnosis<br>8% worsened diet<br>56% no change	*Intra-individual*: Fear of cancer recurrence was not associated with changes in diet.	
Humpel et al. (2007)[78]<br><br>Australia	Cross-sectional	113 cancer survivors<br><br>41.6% breast, 14.9% melanoma, 10.9% cervical, 6.9%	*Socio-demographic:* Marital status, employment status, age and educational level	Participants were asked whether the cancer diagnosis influenced changes in their diet (the amount of fat, fiber, fruits,	*Socio-demographic:* No significant differences were found for marital status and education level.	657 study participants, of which 113 cancer survivors. Only results for cancer survivors reported.

		colon/rectal, 5.9% ovarian, and 19.8% other<br><br>86.7% female<br><br>49.6% 55 years or older<br><br>71.2% more than 2 years after diagnosis		and vegetables, takeaway) and when these changes had occurred (within 1 month, 6 months, much later, or never).<br><br>The majority made positive dietary changes after diagnosis. Most made changes within 6 months of their diagnosis. Within 1 month of diagnosis, 59.0% decreased fat, 69.1% decreased takeaway, 63.2% increased fiber, and 72.7% increased fruit/vegetables. Within 6 months of their diagnosis, 88.7% decreased fat intake, 85.5% decreased takeaway food, 89.7% increased fiber intake, 90.9% increased fruit/vegetable intake.	Age:<br>Older cancer survivors were more likely to decrease their intake off fat ($p<0.01$), amount of takeaway food ($p < 0.05$), increase fiber ($p < 0.01$) and fruit/vegetable intake ($p<0.05$).<br><br>Employment status:<br>More employed individuals increased the intake of fiber compared to those not employed ($p<0.05$).
Kassianos et al. (2017)[92]<br><br>UK	Cross-sectional	95 prostate cancer patients<br><br>Mean age 68.6 (SE 0.7)<br>Range 55 - 93 years<br><br>Mean years since diagnosis 4.5 (SE 0.4)<br><br>84.2% retired/ not working<br><br>80% married/living with another adult	*Socio-demographic:*<br>Educational level<br><br>*Intra-individual:*<br>Perceived behavioural control (18- item Form C of the Cancer Locus of Control Scale)<br><br>Health related quality of life (30-item EORTC QLQ C30 and the two functioning scales (sexual activity, sexual functioning) from the prostate cancer- specific EORTC QLQ PR25.	Participants were asked whether they changed their diet after diagnosis and after therapy started. In order to assess whether patients made healthy or unhealthy changes, a retrospective question was used. Participants were asked to rate the consumption of seven food items (fruits, vegetables, red meat, dairy products, alcohol, sweets, fish) on a 7-point Likert scale ranging from "very much less" to "very much more" with the middle option being "the same" to assess no change.<br><br>Few (0%–6.4%) participants initiated unhealthy changes either post-diagnosis or post- therapy compared to healthy changes (43.2%–59.6%) whereas	*Sociodemographic:*<br>Patients who reported changes on their diet post-diagnosis were more *highly educated* than those who did not ($p < .01$. This was not found for patients who reported changes post-therapy.<br><br>*Intra-individual:*<br>Patients who changed their diet after diagnosis reported significantly lower levels of *cognitive functioning* than those who did not ($p = .02$). They also reported significantly lower levels of *external locus of control* (doctors) than those who did not ($p = .01$). Patients who changed their diet after therapy reported significantly lower levels of *cognitive functioning* than those who did not ($p < .001$) and lower levels of *emotional functioning* ($p = .02$). They also scored

				almost one in two participants reported no changes in any of the food items postdiagnosis or post- therapy (39.4%–51.5%). None of the participants increased red meat consumption post-diagnosis (0%) and only 1% post-therapy. Decrease in red meat consumption was the most frequently reported healthy dietary change (56.8%– 59.6%).	significantly lower on *general QoL* (p = .04) and had significantly lower *external locus of control* (doctors) (p = .04).	
Kwarteng et al. (2020)[35]<br><br>USA	Intervention study (RCT)<br><br>6-month weight loss intervention	246 female overweight African-American breast cancer survivors who completed treatment at least 6 months before recruitment (Intervention: n= 125, Control: n= 121)<br><br>Mean (SD) age 57.5 (10.1) years<br><br>Mean (SD) BMI 36.1 (6.2) kg/m^2	*Inter-individual:* Social support (The Social Support questionnaire)<br><br>*Intra-individual:* Nutrition self-efficacy (assessed using an 11-item instrument by Latimer et al., 2011), perceived access to healthy eating, and perceived neighborhood safety	Healthy Eating Index (HEI) was calculated by Nutrition Quest using the interviewer administered Block 2005 Food Frequency Questionnaire<br><br>HEI at baseline (Range 38.9–93.9) Mean 65.1 (SD 11.1)<br><br>6-month HEI change: Mean 5.0 (SD 10.1)	*Inter-individual:* Lower friend support for eating habits-discouragement (p = 0.014) was associated with improvements in diet.<br><br>*Intra-individual:* Higher self-efficacy (p < 0.001) was associated with improvements in diet.<br><br>Formal mediation tests were conducted to examine whether self-efficacy and social support mediated the association between group and change in diet. No evidence to suggest a mediating effect was found.	Only overweight/obese survivors were included (BMI ≥ 25 kg/ m2)<br><br>Control condition was a Self-Guided Group
Kwarteng et al. (2021)[36]<br><br>USA	Intervention study (RCT)<br><br>6-month weight loss intervention	246 female overweight African-American breast cancer survivors at least 6 months post-treatment<br><br>Intervention: n=125 Control: n=121<br><br>Mean (SD) age 57.5 (10.1) years Range 30.6–82.2	*Intra-individual:* Contemporary life stress (Crisis in Family systems)<br><br>Over half of the women (57%) experienced changes in their relationship, such as getting divorced or breaking up with a partner. Nearly half (46%) had something in their neighborhood happen that made them feel unsafe. Over a third (39%) experienced financial changes such as missing a	Dietary changes were measured by calculating Healthy Eating Index (HEI) by NutritionQuest using the interviewer administered Block 2005 Food Frequency Questionnaire.<br><br>Intervention group (n=125): Baseline: 65.7 ± 11.4 6-month change: 6.4 ± 10.0 12-month change: 5.0 ± 9.5<br><br>Control group (n=121):	*Intra-individual:* *Stress* was not associated with dietary changes in the intervention group or control group. Stress was not associated with maintenance of dietary changes.	Only overweight/obese survivors were included (BMI ≥ 25 kg/ m2)<br><br>Control condition was a Self-Guided Group

			rent or mortgage payment or had their electricity cut off. Over a third (34%) had a family member die or become ill.	Baseline: 64.4 ± 10.8<br>6-month change: 3.3 ± 10.1<br>12-month change: 3.8 ± 10.8		
Maunsell et al. (2002)[70]<br><br>Canada	Intervention study (RCT)	250 women with newly diagnosed, nonmetastatic breast cancer	*Socio-demographic:* Age, education, income<br><br>*Intra-individual:* Psychological distress at diagnosis (Psychiatric Symptom Index - PSI), stressful life events in the past 5 years (modified version of the Life Experiences Survey)	Participants were asked "During the past 12 months, have you made any changes to your diet?" to determine whether women had initiated dietary changes since diagnosis. If yes, the interviewer asked about specific changes in consumption of meat, fish, fruit and vegetables, legumes, dessert, alcohol, vitamins, dairy products, and cereals and bread.<br>To assess the direction of changes, women were asked whether consumption had increased, decreased, or was introduced or eliminated in the period since diagnosis.<br><br>41% reported making dietary change. Meat intake was the most common change (77%), followed by fruit and vegetables (72%) and dessert (66%). A change in alcohol consumption was reported by 19%.	*Socio-demographic:* Women initiating dietary change were more likely to be *younger*. 50% of women aged less than 50 years initiated dietary change, compared with 42% and 16% among those aged 50 to 69 and >=70 years, respectively.<br><br>Education and income NS<br><br>*Intra-individual:* higher initial *psychological distress*, a *greater number of stressful events in the 5 years preceding diagnosis* were associated with initiating dietary change.<br>In analyses adjusted for possible confounders, women who reported initiating dietary changes had a significantly higher mean decrease in *psychological distress* (average 9.0 points) in the year after diagnosis than those who did not make dietary changes (average 4.7 points; P =.03).	Data used in these analyses came from 250 women who completed all three study interviews: the first immediately after obtaining consent, and 3 and 12 months later. These women represent 89% of the consecutive series of 282 women initially eligible for a randomized trial assessing effects on quality of life in the year after breast cancer diagnosis of a monthly telephone psychological distress screening program.
Mosher et al. (2008)[47]<br><br>USA	Intervention study (RCT)	543 breast and prostate cancer patients, randomly assigned to receive a 10 month program of tailored mailed print materials that aimed to	*Intra-individual:* For each of the goal behaviors, participants rated their *self-efficacy* in response to the	Telephone surveys conducted at baseline and 1 year assessed dietary practices and self-efficacy.	*Intra-individual:* Changes in *self-efficacy* for fat restriction and eating more	Baseline values of the mediator and dependent variables were included as covariates in all analyses; thus, the

		increase fruit and vegetable consumption, reduce fat intake, and/or increase exercise (intervention group, n=253) or a 10-month program of publically available materials on diet and exercise (attention control group, n=266).<br><br>Mean age 57.0 years (SD10.8)<br><br>Mean time since diagnosis 3.83 months (SD 2.74)	question, 'How sure are you that you could (exercise at least 30 minutes a day at least 5 days a week; eat at least 5 servings of fruit and vegetables per day; or eat a low-fat diet)?' Participants responded to each question on a 5-point scale from 1 (very unsure) to 5 (very sure). Descriptions of exercise, fruit and vegetable servings, and a low-fat diet were provided prior to each question.	Dietary outcomes: Participants completed a modified version of the Diet History Questionnaire (DHQ)<br><br>Mean Diet Quality Index-revised score<br>-Intervention:<br>Baseline: 66.5(SD11.3)<br>1 year: 72.8(SD10.6)<br>-Attention control:<br>Baseline: 66.9 (SD9.5)<br>1 year: 68.7 (SD10.9)<br>Mean total percent of calories from fat<br>-Intervention:<br>Baseline: 38.0 (SD5.7)<br>1 year: 33.7(SD5.7)<br>-Attention control:<br>Baseline: 37.8 (SD5.6)<br>1 year: 35.7 (SD4.6)<br><br>Mean no. of daily servings of fruit and vegetables<br>-Intervention:<br>Baseline: 5.1 (SD2.7)<br>1 year: 6.2 (SD2.8)<br>-Attention control<br>Baseline: 5.0 (SD2.3)<br>1 year: 5.6 (SD2.6)	fruits and vegetables were significant mediators of the intervention's effects on dietary outcomes at 1-year follow-up.<br><br>Experimental participants endorsed greater changes in self-efficacy for fat restriction and eating more fruit and vegetables than did control participants, which, in turn, were associated with better diet quality.	effects observed represent change over the course of the intervention.
Mosher et al. (2013)[48]<br><br>USA	Intervention study (RCT)	Breast and prostate cancer survivors (N=489), randomly assigned to receive a 10-month program of tailored mailed print materials that aimed to increase fruit and vegetable consumption, reduce fat intake, and/or increase exercise (intervention group, n=236) or a 10-month program of publically available materials on diet and exercise (attention control group, n=253).<br>Mean age 57.2 years (SD=10.7)<br><br>Mean time since diagnosis 3.9 months (SD=2.8) at the time of study enrollment.	*Intra-individual:*<br>Same as Mosher et al. (2008) for *self-efficacy.*<br><br>+ *Change in barriers*<br>An author-constructed barriers questionnaire assessed 16 common barriers to physical activity, 12 common barriers to fruit and vegetable consumption, and nine common barriers to adhering to a low-fat diet. For each item, participants were asked to indicate 'yes, it was a reason for them not practicing the goal behavior' or 'no, it was not a reason for them not	See Mosher et al. (2008)<br><br>Mean Diet Quality Index-revised score<br>-Intervention:<br>Baseline: 66.6 (SD11.1)<br>2 years: 71.5(SD10.5)<br>-Attention control:<br>Baseline: 67.0 (SD9.4)<br>2 years: 68.9 (SD10.6)<br><br>Mean total percent of calories from fat<br>-Intervention:<br>Baseline: 38(SD 5.7)<br>2 years: 36.5 (SD 6.6)<br>-Attention control:<br>Baseline: 37.8 (SD5.6)<br>2 years: 38.0 (SD5.4)	*Intra-individual:*<br>Change in *self-efficacy for fat restriction* partially explained the intervention's effect on *fat intake.*<br>Change in *self-efficacy for fruit and vegetable consumption* partially explained the intervention's effect on daily *fruit and vegetable intake*.<br>Change in *self-efficacy for fat restriction* partially accounted for the intervention's impact on *overall diet quality* among men only.<br><br>*Change in barriers to fat restriction* from baseline to year 1 predicted the percentage of kcal from fat at year 2 when controlling for baseline levels of the	Same intervention as Mosher et al (2008); long-term outcomes 2-year follow-up. Results replicate the mediating effects of self-efficacy on fruit and vegetable intake and fat restriction found at 1-year follow-up (Mosher et al 2008) and extend these findings by suggesting that change in self-efficacy may play an important role in the durability of dietary gains.<br><br>Baseline values of the mediator and dependent variables were included as covariates in all analyses; thus, the effects observed represent change

			practicing the goal behavior'.	Mean no. of daily servings of fruit and vegetable intake<br>-Intervention:<br>Baseline: 5.1 (SD2.7)<br>2 years: 6.0 (SD 2.7)<br>-Attention control:<br>Baseline: 5.0 (SD2.3)<br>2 years: 5.7 (SD2.8)	dependent variable. In the total sample, *change in barriers to fat restriction* was positively associated with the percentage of kcal from fat. Also, changes in *barriers to fat restriction and fruit and vegetable intake* were negatively associated with diet quality.	during the study period.
Shaffer et al. (2016)[49]<br><br>USA	Longitudinal observational study	108 colorectal cancer patients and 162 caregivers (92 dyads)<br><br>Mean age patients 62.07 (SD 12.07)<br><br>Mean days since diagnosis at T1: 55.29 (SD 40.16)<br><br>31.5% female	*Intra-individual:* Cancer-related stress (six-item Appraisal of Cancer Experience Scale)	Participants' consumption of fruits and vegetables was assessed by their responses to ''How many days per week do you eat at least five servings of fruits and vegetables?'' The count of number of days per week meeting the recommended guidelines (i.e., greater than five servings of fruit and vegetables) was measured at each time point: at two (T1), six (T2), and 12 months post-diagnosis (T3)<br><br>Both patients and caregivers consumed five or more servings of fruits and vegetables approximately 3 days per week on average during the first year after the diagnosis. Fewer than 10 % of patients and caregivers reported consuming five or more servings of fruits and vegetables daily.	*Intra-individual: Patients'cancer-related stress* was significantly positively associated with their own change pattern in fruit and vegetable consumption, which decreased from T1 to T2 and increased from T2 to T3. In other words, patients who reported greater cancer–related stress at T1 showed a greater decrease in their FVC during the first 6 months following the diagnosis when the majority of cancer treatment was underway, yet a greater increase in FVC by the end of the first year when the treatments typically ceased.<br><br>For patients, those with caregivers who reported greater stress from cancer showed a greater decrease in their fruit and vegetable consumption during the first 6 months following the diagnosis, yet a greater increase in fruit and vegetable consumption by the end of the first year.<br><br>Findings suggest that perceived stress from cancer hinders fruit and vegetable consumption around the diagnosis, but motivates positive dietary changes by the end of the first year.	

Shi et al. (2018)[50]<br><br>USA	Intervention study (RCT)	Hispanic/Latina breast cancer survivors (n=70) were randomized to receive either a 12-week theory- and culturally-tailored dietary change program (intervention group, n=34), or standard-of-care printed recommendations (control group, n=36)<br><br>Mean age 56.6 years (SD=9.7 years)<br><br>Mean time since diagnosis 3.4 years (SD=2.7 years)<br><br>Mean body mass index 30.9 kg/m2 (SD=6.0 kg/m2)	*Intra-individual:*<br>A survey instrument was designed to measure theory-based potential mediators based upon Social Cognitive Theory and the stages of change construct of the Trans-Theoretical Model. Participants were asked to rate their agreement with statements addressing specific behavioral mediators, including *stages of change*, *healthy food beliefs*, *self-efficacy of fruit and vegetable intake*, *behavioral capabilities*, and *perceived barriers*, including difficulty finding fruit and vegetables in the neighborhood, difficulty eating fruit and vegetables as snack, taste and snack preferences for fruit and vegetables, and family opinions on fruit and vegetables. *Additional psychological variables were assessed, including survivor concerns, anxiety and depression, and locus of control.*	Fruit/vegetable intake, % calories from fat, and hypothesized psychosocial mediators were assessed by study interviewers at baseline, 6 and 12-months using three 24-hour recall assessments (2 weekdays, 1 weekend day)<br><br>Mean intake of 3.1 servings of fruit and vegetables (SD=1.9) per day. No statistically significant differences between the intervention and control groups.<br><br>The intervention group compared with control group reported an increase in mean servings of fruit and vegetables (6-month: +2.0 vs -0.1, P<0.01; 12-month: +2.0 vs. -0.4, P<0.01), and targeted fruit and vegetable intake (+2.3 vs. -0.1; P<0.01) from baseline.	*Intra-individual:*<br>At 6 months, mediation analysis showed no indirect effect through the hypothesized psychosocial mediators. There was a trend suggesting that improved self-efficacy mediated 40% of the total intervention effect on target fruit and vegetable intake at 6 months.<br><br>Among the hypothesized mediators of the intervention effect, *improved taste/snack-preference for fruit and vegetables* mediated nearly a quarter of the total intervention effect at 12 months. At 12 months, the intervention was associated with an increase in 0.5 fruit and vegetable serving/day through improved taste/snack-preference for fruit and vegetables at 6 and 12 months.<br>In sensitivity analyses, the mediation effect of taste/snack -preference for fruit and vegetables was primarily attributed to preference for snacking on fruit and vegetables. Further, increased taste/snack-preferences for fruit and vegetables was a significant mediator of targeted fruit intake, but not targeted vegetable intake, suggesting that the intervention effect may have had more of an effect on increasing taste and snacking preferences for fruits instead of vegetables.	Inclusion criteria included average intake of <5 servings of F/V per day.
Shi et al. (2020)[44]<br><br>USA	Population-based prospective cohort study	2865 newly diagnosed female *breast* cancer patients	*Socio-demographic*: Education, income, and age	Dietary history was collected using a 139-item modified version of the Block 2005 food frequency questionnaire	*Fruit and vegetable intake:*<br>In multivariable analyses, compared with the low increasers of fruit and	

		Mean age at diagnosis 61 years (range 26–94 years)<br><br>Mean time since diagnosis 1.8 months, range 0.3–7.2 months.<br><br>Pathways Study participants who completed ≥ 2 food frequency questionnaires at the time of breast cancer diagnosis (baseline), and at 6 and 24 months after baseline.<br><br>In-person baseline interview to collect demographic, lifestyle behaviors, psychosocial, and anthropometric information. Follow-up data were collected via mailed questionnaires with interviewer assistance at 6 and 24 months.	*Inter-individual*: Social support (Medical Outcome Study Social Support Survey Instrument)<br><br>*Intra-individual:* Depressive symptoms (Center for Epidemiological Studies Depression Scale - CES-D), and dispositional optimism (Life Orientation Test – LOT)	Trajectory groups of daily fruit and vegetable intake (n=3), % calories from dietary fat (n=4), and alcohol intake (n=3) over 24 months were identified.<br><br>All 3 fruit and vegetable trajectory groups reported slightly increased fruit and vegetable intake post-diagnosis (mean increase = 0.2–0.5 serving/day), while 2 groups (48% of participants) persistently consumed < 4 servings/day. Dietary fat intake did not change post-diagnosis, with 45% of survivors maintaining a high-fat diet (> 40% of calories from fat). While most survivors consumed < 1 drink/day of alcohol at all times, 21% of survivors had 1.4-3.0 drinks/day at baseline and temporarily decreased to 0.1–0.5 drinks/day at 6 months.	vegetable intake, the high and medium increasers were more likely *higher educated,* have a *higher household income,* to report *higher dispositional optimism*, and perceived *greater social support* at baseline.<br><br>*Dietary fat intake:* All NS<br><br>*Alcohol intake:* Temporary decreasers of alcohol intake after diagnosis were more likely to have a *higher education* and a *higher household income*.	
Steinhilper et al. (2013)[80]<br><br>Germany	Longitudinal prospective study	229 female *breast* cancer survivors<br><br>Mean age 53.2 (SD 9.5) years<br><br>Mean time since surgery at follow-up 14 (SD 3.6) months	*Socio-demographic:* Age, educational level, job position, employment status, marital status	Food intake was assessed by means of personal interviews immediately after surgery (T0) and 14 months later (T1). Food intake was assessed by respondents' consumption of 19 different food items or food groups (e.g. ''How many servings of the following fruits do you usually eat per day?''). According to the guidelines for a balanced and wholesome diet issued by the German Nutrition Society (DGE), nutrition was classified into six different food groups: bread, dairy products, fruits,	*Socio-demographic:* All NS	A large proportion of women already met the recommended levels at baseline and mostly maintained over the observation period. With the exception of bread, dairy products and snacks, the number of women meeting the recommendations generally increased from baseline to follow-up. However, statistical significant increases were only seen in meeting fruit recommendations (p<0.01).

				vegetables, meat, and snacks.<br>One-year changes were obtained by subtracting values at baseline from those from the second interview.<br><br>Mean baseline dietary intake (servings per day):<br>Bread (s/d) 1.5 (SD 0.6)<br>Diary (s/d) 3.0 (SD 1.5)<br>Fruit (s/d) 3.0 (SD1.7)<br>Meat (s/d) 0.7 (SD1.4)<br>Mean occasions per week:<br>Snacks 4.3 (3.9)<br>Fruit 10.5 (5.8)<br>Vegetable 8.3 (3.2)<br><br>Change over time:<br>Bread 0.0 (0.6) NS<br>Dairy products -0.3 (1.4)*<br>Fruit (s/d) 0.4 (1.8)*<br>Fruit 0.9 (5.8)*<br>Vegetable 0.7 (3.5)*<br>Meat -0.1 (0.5)*<br>Snacks 0.2 (4.2) NS<br>*p<0.05		
Wang et al. (2015)[51]<br><br>USA	Intervention study (RCT)	Breast cancer survivors (N= 2817)<br><br>Intervention group: n=1398<br>Comparison group: n=1419<br><br>Age<br>>=50 years 64 %<br><br>BMI<br>42 % normal weight 31 % overweight<br>27 % obese	*Intra-individual:*<br>Depressive symptoms (six-item short form of the Center for Epidemiologic Studies Depression Scale - CES-D)<br><br>Baseline: 20% depressive symptoms (CES-D scores ≥5)<br><br>The prevalence of depressive symptoms declined in the intervention group in the first year by −3.7 % (p=0.03) but reverted to baseline levels in year 4 (p= 0.56). No such change was observed in the comparison group.	24 h dietary recalls and clinic measurement visits at baseline, 1 year, and 4 years.<br><br>Plasma carotenoid concentration (known biomarkers of fruit and vegetable intake) (μmol/L)<br>Mean (SE)<br>Baseline:<br>Intervention 2.3 (0.04)<br>Comparison 2.3 (0.04)<br>Year 4:<br>Intervention 3.2 (0.07)<br>Comparison 2.2 (0.04)*<br><br>Fiber, mean g/day (SE)<br>Baseline:<br>Intervention 21.1(0.2)<br>Comparison 21.2 (0.2)<br>Year 4:	*Intra-individual:*<br>Among those with better *vegetable/fruit intake* and higher baseline *fiber consumption* at baseline, baseline depressive symptoms were not associated with either 1- or 4 year change.<br>Among those who consumed less *fat* at baseline, depressive symptoms were not associated with decreases achieved in the first year; however, it was associated with the increase observed between years 1 and 4 (+37.9 vs. +27.3%, p=0.04).<br><br>A different pattern was observed in those who were further from the study's targeted dietary pattern at baseline. Baseline	Secondary analyses from the Women's Healthy Eating and Living (WHEL) Study

				Intervention 25.2 (0.3)<br>Comparison 19.3 (0.2)*<br><br>Percent energy from fat, mean (SE)<br>Baseline:<br>Intervention 28.4(0.2<br>Comparison 28.7(0.2)<br>Year 4:<br>Intervention 27.2 (0.2)<br>Comparison 31.4 (0.2)<br><br>*All p<0.0001 intervention vs comparison 4 year vs. baseline	depressive symptoms did make a difference in the amount of change achieved in the first year, but not between years 1 and 4. For those with *low baseline vegetable intake*, the first-year increase in plasma carotenoid concentration was lower for those with baseline depressive symptoms than that for those without (37.2 vs. 69.7%, p=0.02). In the first year, baseline depressive symptoms were associated with smaller increases in *fiber consumption* (+49vs. +61.2%, p<0.01) and smaller decreases in *energy consumed from fat* (−22.4 vs. −26.7, p=0.02).	
Wilkinson et al. (2012)[46]<br><br>USA	Intervention study (RCT)<br><br>1-year tailored intervention vs. standardized intervention	452 *breast* and *prostate* cancer survivors within 9 months of diagnosis<br><br>Intervention: n = 224<br>Control: n = 228<br><br>53.7% female<br><br>Mean (SD) age at diagnosis 57.9 (10.6) years; Range 22-85<br><br>BMI<br>25.0-29.9: 37.7%<br>>30.0: 24.8%	*Intra-individual:* Cancer coping style (15-item version of Mini-MAC was used to classify participants as fighting-spirits, fatalist or other)<br><br>Follow-up at completion of the interventions (1 year) and after an additional year long observation period (2 years)	Dietary changes assessed with a modified version of the National Cancer Institute Diet History Questionnaire.<br><br>Baseline:<br>>= 5 daily servings of fruit and vegetables 51.3% (n=322)<br><br>Mean daily fruit and vegetable consumption 5.6 (SD 2.9)<br><br>Low fat diet (<30% of kcal) 17.2% (n=108)	*Intra-individual:* Several differences by coping style were observed among standardized intervention participants. At year 1, fatalists reported more servings of *fruit and vegetables/day* than the fighting-spirits (p<0.01), though this difference did not result in a higher proportion achieving goal behavior.<br>By year 2, fatalists not only reported more fruit and vegetables than the fighting-spirits (p<0.01), a higher proportion also met the fruit and vegetable goal (p<0.05).<br><br>No differences were observed between *fat intake* or achieving the fat consumption goal and coping style among control participants throughout the study.<br><br>The proportion of fatalists in the standardized intervention group	Alpha-carotene assays, indicative of fruit and vegetable intake, were performed on plasma obtained from a 25% subsample at all three time-points and showed a significant association with fruit and vegetable intake.

	meeting the *fruit and vegetable goals* continued to increase through year 2, whereas the proportion of fighting-spirits meeting these goals peaked at year 1 and then declined.	
