# Supplementary material for: Psychosocial Determinants of Lifestyle Change after a Cancer Diagnosis: A Systematic Review of the Literature
Source: Cancers (Basel). 2022 Apr 16;14(8):2026. doi: 10.3390/cancers14082026 (PMC9032592; doi:10.3390/cancers14082026)
Supplement: Supplementary file 1 [file cancers-14-02026-s001.zip › Table S3_MH.pdf]

Table S3. Overview of quantitative studies assessing psychosocial determinants of changes in smoking cessation among cancer survivors (n=12).

First author (year) Country	Study design	Sample Characteristics *(at baseline)*	Psychosocial variables *Type(s); Assessed at; Assessed with*	Lifestyle *Assessment instruments Baseline; Change (period)*	Findings	Comments
Bidstrup et al. (2013)[93] Denmark	Cohort study	449 women with *breast* cancer<br>Mean (SD) age 56.7 (4.1) years<br>Mean BMI 25.3 kg/m^2 at baseline	*Socio-demographic:* Marital status (categorized into never married, divorced or widowed, and married or with registered partner)., and educational status (categorized into basic school/high school, vocational training and higher education).	At both baseline and follow-up, participants were asked to indicate how much they smoked per day in terms of cigarettes (1 g tobacco/cigarette), cigars (4.5 g tobacco/cigar), cheroots (3 g tobacco/ cheroot), and pipes (3 g tobacco/pipe), and these were summed as grams of tobacco per day<br>68% did not smoke at baseline and this significantly increased to 74% at follow-up (p< .001)	No significant changes in tobacco consumption by *level of education*, and *marital status.*	
Cooley et al. (2007)[52] USA	Prospective longitudinal study	230 women with *lung* cancer<br>Mean (SD) age 65 (11.65) years<br>Mean (SD) time since diagnosis 23.04 (15.5) months<br>13% (30/230) of women with lung cancer were never-smokers and 87% (200/230) were ever-smokers.	*Socio-demographic:* Age, marital status, educational level, and smokers in household<br>*Intra-individual:* Depression (CES-D, >15)	Baseline, 3 and 6 months Continued smoking<br>Smoking status (current, former, and never) was determined through self-report and biochemical verification with urinary cotinine.<br>Of those with complete data (n=165), 7% were smoking at baseline, 7% were smoking at 3 months follow-up, and 8% were smoking at 6 months.	*Age* (OR .94, p =.01), *household member smoking* (OR 3.55, p = .033) and *depression* (OR 3.9, p =.014) were associated with continued smoking	
Emmons et al. (2009)[53] USA	randomized control trial with two conditions, peer phone counseling (PC) and self-help (SH)	566 childhood cancer survivors having completed long term follow-up<br>Intervention group: Mean age 31 (SD 6.5) 50% female<br>Control group: Mean age 31 (SD 6.9) 52% female	*Socio-demographic:* Age, sex, race, ethnicity, socioeconomic status, education, employment history, marital status, and medical history.<br>*Intra-individual:* Self-efficacy was defined using single-item measures of confidence in one's ability to quit smoking in at 1 and 6 months, and confidence in not	Smoking status based on 7-day point-prevalence smoking status at the end of the intervention and at long-term follow-up.<br>Quit attempts were evaluated among continuous smokers and relapsers (categorized into 0, 1 to 2, 2 to 5, and 6+ attempts).	19% of all participants reporting having quit smoking at the LT follow-up. Quit rates at LT follow-up were significantly higher in the PC condition compared to SH (20.6% *v* 17.6%; p =.0003). Significant relationships between smoking status at LT follow-up and *educational level* (p = .017), *situational confidence* (p < .001), short-term *self-efficacy* (p <.01),	

			smoking in a variety of situations.23<br><br>Readiness to quit smoking was assessed using the stages of change algorithm.24<br><br>Depressed mood was assessed using a single item reflecting feelings of being downhearted and blue in the previous 2 weeks		and *readiness to change* (p = .02). Self-efficacy, and readiness to change was associated with quit attempts
Foshee et al. (2017)[54]<br><br>USA	prospective, randomized, controlled trial	52 patients with *laryngology* successfully completed the long-term follow-up phone surveys<br><br>73% 50 years or older<br><br>55.8% female	*Intra-individual:* Motivation and readiness to quit smoking were assessed using a Likert scale ranging from 1 (not planning to quit) to 4 (actively trying to quit)	The follow-up phone surveys aimed to collect information on smoking status	Those patients who were actively trying to quit at study enrollment appeared more likely to successfully quit smoking compared with those participants who would only consider quitting (35.7% vs. 20%), but this difference was not significant (p = 0.70).
Gritz et al. (1998)[55]<br><br>USA	Intervention study RCT Physician-delivered, smoking cessation intervention	83 Current smokers and recent quitters (individuals who stopped smoking within the year prior to diagnosis), newly diagnosed with first primary squamous cell carcinomas (SCC) of the *head and neck.*<br><br>Mean age 57.1 years<br><br>34.8% female	*Baseline*<br>Stage of change, self-efficacy, mental distress (Profile of Mood States)<br>*Follow-up (1, 6, 12 months)*<br>The measures collected at baseline were repeated at each assessment. Additional follow-up measures included an assessment of cessation techniques, coping behaviors to resist smoking, social support, self-efficacy for staying off cigarettes, withdrawal and craving, and the relapse episode.	Relapse was reported retrospectively at the follow-up most proximal to the event (1, 6, or 12 months).	21 of the 83 patients relapsed.<br>The higher the *stage of change* (readiness to quit), the less likely the patient is to relapse (OR= 0.17, 95% CI: 0.06, 0.50).<br>*Coping behaviors* used to resist smoking did not differ significantly between groups.<br>At the one-month follow-up, high levels of positive *supportive behaviors* and low levels of *unsupportive behaviors* were reported overall, with few between-group differences.<br>At the one-month follow-up, continuous abstainers expressed significantly higher levels of confidence than patients who relapsed in the ability to stay off cigarettes for the next 1 (p = .001), 6 (p = .013), and 12 (p = .01) months.
Kashigar at al. (2013)[71]<br><br>Canada	Longitudinal observational study	295 newly diagnosed *head and neck* cancer patients<br><br>Mean time since diagnosis 2.9 months at baseline<br><br>Follow-up questionnaire a mean 24.5 months (range, 6-	*Inter-individual:* Social support as defined by Pomerleau et al. (2004), and social smoking environment	The follow-up questionnaire assessed the date last smoked and current smoking habits.<br><br>49% were smokers at diagnosis and 50% quit after diagnosis.	Among current smokers, patients were more likely to quit by the time of follow-up if they had a spouse who did not smoke (OR = 4.25, 95% CI [1.70-10.6]), and fewer peers who smoked (OR =2.32, 95% CI [1.00-5.37]). Social support was not a significant predictor of quitting smoking.

		43 months) after the baseline questionnaire.				
Schnoll et al. (2011)[56]<br><br>USA	double-blind, placebo-controlled smoking cessation	246 cancer patients<br><br>35% tobacco-related (head and neck or lung), 21% breast, 15% prostate, 9% lymphoma, 5% colorectal, 4% kidney, pancreas or liver, 3% genitourinary, 3% esophageal, and 5% other<br><br>48% female<br><br>Mean age 54.8 years	*Socio-demographic:* Age, and gender	7-day point-prevalence abstinence at Week 12 and 27, confirmed with breath CO (abstinence = B 10 ppm).<br><br>The quit rate at week 12 was 26% and the quit rate at week 27 was 18%	12 week cessation was associated with *age* (OR 1.06, p = 0.01).<br>27 week cessation was associated with *age* (OR = 1.04, p = .03) and *gender* (M) (OR = 0.47, p = 0.04).	Participants included in this study were self-identified smokers and interested in quitting and had to be smoking at least 2 cigarettes/day on average
Schnoll et al. (2010)[57]<br><br>USA	double-blind placebo-controlled randomized trial<br><br>placebo or bupropion, stratifying by pre-treatment depression symptoms.	246 cancer patients<br><br>35% tobacco-related (head and neck or lung), 21% breast, 15% prostate, 9% lymphoma, 5% colorectal, 4% kidney, pancreas or liver, 3% genitourinary, 3% esophageal, and 5% other<br><br>48% female<br><br>Mean age 54.8 years	*Intra-individual:* Depression (CES-D) Fifty-five patients (22% of the sample) had CES-D scores of >16.	7-day point-prevalence abstinence at Week 12 and 27, confirmed with breath CO (abstinence = B 10 ppm). Daily smoking was also assessed using a validated time-line follow-back method<br><br>Intervention group:<br>Week 12: 27.2% abstinence rate<br>Week 27: 18.4% abstinence rate<br><br>Control group:<br>Week 12: 24.2% abstinence rate<br>Week 27: 17.4% abstinence rate	Patients with *depression symptoms* reported significantly lower abstinence rates vs. patients without *depression symptoms* (OR = .14, 95% CI: 0.02–0.80, p = .03).<br>At Week 12, 10.9% of depression symptom patients were abstinent vs. 29.8% of the non-depression symptom patients (p = .002).<br>At Week 27, 10.9% of depression symptom patients were abstinent, vs. 19.9% of the non-depression symptom patients (p = .11).	Participants were included if they smoked more than 2 cigarettes/day on average.
Schnoll et al. (2005)[58]<br><br>UK	Randomized Pilot Study	109 patient with *head and neck* or *lung* cancer<br><br>Intervention group: Mean (SD) time since diagnosis 24.5 (33.5) months<br>51.9% female<br>Mean (SD) age 58.7 (9.0) years<br><br>Control group: Mean (SD) time since diagnosis 11.33 (19,95) months<br>40.4% female<br>Mean (SD) age 57.7 (10.1) years	*Intra-individual:* Self-efficiacy (scale is based on Marshall's general health-related self-efficacy survey)<br><br>Perceptions of risk. *(*Seven Likert-type items)<br><br>The pros and cons of quitting (Eight items)<br><br>Fatalism (The Powe Fatalism Inventory)<br>Emotional Distress (POMS)<br><br>Stage of change (quit motivation)	Smoking status was assessed by asking patients if they smoked a cigarette, even a puff, in the last 30 days.<br><br>46.2% reported quitting smoking at 1-month follow-up<br>41.1% reported quitted smoking at 3-month follow-up	Cessation at 1 month was associated with *quit motivation* (OR = 3.18, 95% CI = 1.51, 6.71).<br>Cessation at 3 months was associated with *quit motivation* (OR = 3.26, 95% CI =1.46, 7.30) and *cons of quitting* (OR = 0.29, 95% CI = 0.08, 1.01)	A biochemical verification of smoking status was not conducted
Simmons et al. (2020)[59]	RCT	412 patients newly diagnosed with cancer who had	*Socio-demographic:* Age, gender, race,	Follow-up at 2, 6 and 12 months	Of the 12 prospective moderators only *marital/*	Control condition consisted of usual care

USA	Smoking relapse prevention intervention	recently quit smoking<br><br>Mean (SD) age 55.0 (10.8) years<br><br>52.0% female	marital status, and education.<br><br>*Intra-individual:* Fears of cancer recurrence, risk perception of smoking after cancer, depressive symptoms (CES-D), cessation self-efficacy (7-point Likert scale assessing confidence in not smoking within the next 6 months),pain (BPI), and fatigue (BFI)	Self-reported 7-day point prevalence abstinence (ie, no smoking within the previous 7 days).<br><br>Sub-sample: breath carbon monoxide (CO) samples collected via a portable CO monitor.<br><br>2 months: 73% abstinence rate<br>6 months: 67% abstinence rate	*partnered status* demonstrated a marginally significant interaction with condition ($P = .07$) on abstinence rates over the 2 assessments	
Streck et al. (2021)[60]<br><br>USA	RCT<br><br>Telephone counseling plus cessation medication treatment (Internsive Treatment)	303 newly diagnosed cancer patients<br><br>Median (IQR) age 59 (52 – 65) years<br><br>56% female	*Socio-demographic:* Sex, age, race, and education.<br><br>*Intra-individual:* Stress coping (single item), perceived stress (Perceived Stress Scale), and psychological distress (Distress Thermometer) Anxiety symptoms (GAD-7).	Self-reported past 7-day point prevalence tobacco abstinence. Those who reported abstinence were mailed a saliva kit with instructions to return a sample to assess for cotinine to biochemically verify abstinence.	*Stress coping* ($p < .001$), *perceived stress* ($p <.001$), *distress* ($p< .001$) and *anxiety* ($p < .001$) at 3 months were associated with biochemically confirmed smoking abstinence at 6 months.	Participants were included if they smoked a cigarette in the past 30 days and were willing to consider attempting to quit smoking.<br><br>Control condition consisted of short-term counseling plus medication advise (standard treatment)
Yang et al. (2021)[95]<br><br>Taiwan	prospective observational design	133 newly diagnosed people with lung cancer who recently quitted smoking or were current smokers.<br><br>Mean (SD) age 63.40 (11.70) years<br><br>10.5% female	*Socio-demographic:* Age, sex, education level, marital status, income Status, and second-hand smoke exposure at home<br><br>*Intra-individual:* Self-efficacy for not smoking (Quitting Self-Efficacy Questionnaire), Anxiety and depression (HADS),	After diagnosis and then every month after diagnosis for a total of 6 months, participants were asked whether they smoked during the previous month (yes/no).<br>Patients' levels of exhaled carbon monoxide (CO) were measured at T0 to confirm the self-reported data on smoking status.	Patients who were younger age (OR = 0.95, $p = 0.026$), exposed to second-hand smoke (OR = 3.35, $p = 0.012$), and lower self-efficacy for not smoking (OR = 0.96, $p = 0.011$) were more likely to belong to the class of "indecisive for abstinence."	
