# Supplementary material for: Psychosocial Determinants of Lifestyle Change after a Cancer Diagnosis: A Systematic Review of the Literature
Source: Cancers (Basel). 2022 Apr 16;14(8):2026. doi: 10.3390/cancers14082026 (PMC9032592; doi:10.3390/cancers14082026)
Supplement: Supplementary file 1 [file cancers-14-02026-s001.zip › Table S4_MH.pdf]

Table S4. Overview of quantitative studies assessing psychosocial determinants of changes in alcohol consumption among cancer survivors (n=4).

First author (year) Country	Study design	Sample Characteristics *(at baseline)*	Psychosocial variables *Type(s); Assessed at; Assessed with*	Lifestyle *Assessment instruments Baseline; Change (period)*	Findings	Comments
Bidstrup et al. (2013)[93] Denmark	Cohort study	449 women with *breast* cancer<br><br>Mean (SD) age 56.7 (4.1) years<br><br>Mean BMI 25.3 kg/m^2 at baseline	*Socio-demographic:* Marital status (categorized into never married, divorced or widowed, and married or with registered partner)., and educational status (categorized into basic school/high school, vocational training and higher education).	At baseline, participants were asked to indicate how often they drank each of the following types of alcohol: light beer, ordinary beer, strong beer, wine, fortified wine, and liquor and summed as grams alcohol per day.<br><br>22% drank more than 2 drinks/day (24 g alcohol). The mean alcohol consumption increased slightly by 0.6 g/day.	The changes in alcohol consumption were not significantly associated with education or marital status.	
Hackshaw-McGeagh et al. (2015)[85] UK	Prospective longitudinal observational study	511 men diagnosed with *prostate* cancer<br><br>Mean age 62.3 (range 50 - 70) years<br>33.3% had a healthy BMI with a median (IQR) of 23.5 (22.5 – 24.1) kg/m^2<br><br>66.7% had an unhealthy BMI with a median (IQR) of 28.0 (26.4 – 29.8) kg/m^2	*Socio-demographic*: Áge, marital status, and social class	The amount and frequency of beer, spirits of wine were combined and converted into standard UK alcohol units and categorized into lower (21 or less units/week) or elevated (more than 21 units/week) risk of adverse health effects.<br><br>39.1% consumed over the recommended limit of alcohol at baseline<br><br>Of the "unhealthy" group, 24.5% reduced their alcohol consumption and were classified in the "healthy" group post-diagnosis.<br>Of the "healthy" group 9.6% increased alcohol consumption.	There was no evidence of associations between age, social class, and marital status and "healthy" levels of alcohol consumption post-diagnosis.	
Hall et al. (2019)[31] USA	Cross-sectional observational study	258 early stage cancer survivors who had completed primary treatment<br><br>27% *breast*, 21% *hematologic*, 11% *gynecologic*, 9% *GI*, 8% *genitourinary*, 6% *head and neck*, 6% *melanoma*, 4%	*Intra-individual:* Fear of cancer recurrence (Assessment of Survivor Concerns), emotional distress<br><br>To assess emotional distress participants were asked to rate the extend to which they have felt nervous or worried and sad or	Changes in alcohol consumption were assessed by asking participants how their alcohol consumption had changed compared to before their diagnosis and responses were categorized in to decreased, no change or improved.	Higher fear or recurrence was associated with increased alcohol consumption (r= -0.21, $p < .01$). Higher emotional distress was associated with increased alcohol consumption (r= -0.22, $p < .001$).	

		thoracic and 2% other<br><br>54% 60 years or older<br><br>64% female	depressed over the past month.	21% reported increased alcohol intake, 4% reported decreased alcohol intake and 75% reported no change in alcohol intake after diagnosis.		
Shi et al. (2020)[146]	Prospective cohort study	3000 newly diagnosed female *breast* cancer patients<br><br>Mean age at diagnosis 59 (range 26 – 94) years<br><br>Mean time since diagnosis 1.8 (range 0 – 8) months	*Intra-individual:* Depressive symptoms (Center for Epidemiological Studies Depression Scale), and dispositional optimism (Life Orientation Test)<br><br>*Inter-individual*: Social support (Medical Outcome Study Social Support Survey Instrument)<br><br>*Socio-demographic*: Education, and age	Alcohol consumption was assessed using a 139-item modified version of the Block 2005 food frequency questionnaire and converted into daily ethanol intake (gram).<br><br>3 groups of alcohol intake over time were observed. 79% were low maintainers (1.9 g/day of ethanol over 24 months after diagnosis). 16% were medium temporary decreaser (18.7 g/day at baseline and reduced intake by 4.6-6.1 g/day at 6 months only. 5% were high temporary decreaser (40.9 g/day at baseline with a temporary decrease by 4.6-6.1 g/day only at 6 months)	Temporary decreasers were more likely to have college education relative to those with high school or less (medium temporary decreaser vs. low maintainer: OR= 1.93, 95% CI: 1.18, 3.16). Age, social support, optimism, and depressive symptoms were not significant predictors of alcohol consumption trajectory.	
