# Supplementary material for: Psychosocial Determinants of Lifestyle Change after a Cancer Diagnosis: A Systematic Review of the Literature
Source: Cancers (Basel). 2022 Apr 16;14(8):2026. doi: 10.3390/cancers14082026 (PMC9032592; doi:10.3390/cancers14082026)
Supplement: Supplementary file 1 [file cancers-14-02026-s001.zip › Table S5_MH.pdf]

Table S5. Overview of quantitative studies assessing psychosocial determinants of changes in lifestyle score among cancer survivors (n=4).

First author (year) Country	Study design	Sample Characteristics *(at baseline)*	Psychosocial variables *Type(s); Assessed at; Assessed with*	Lifestyle *Assessment instruments Baseline; Change (period)*	Findings	Comments
Costa et al. (2011)[79] Australia	Observational study	154 *thyroid* cancer patients<br><br>Mean (SD) age 50.96 (14.43) years<br><br>Mean (SD) time since diagnosis 4.83 (4.79) years<br><br>70.1% female	*Socio-demographic:* Age, gender, marital status, education, employment, and income.<br><br>*Intra-indiviudal:* Cancer-related stress (single item), Benefit finding (43-item SRGS-R)	A self-composed survey was used to assess health behavior change. Participants rated their behavior change due to their cancer on a 7-point scale on six health behaviors (diet, exercise, sleep, stress management, alcohol, and smoking). A two-factor solution was found. One factor had four items reflecting lifestyle behaviors (sleep, diet, exercise and stress management), whereas the other included substance use items (alcohol and smoking). Item scores were summed with higher scores reflecting greater positive health behavior change.	In multivariate analyses, benefit finding was associated with a significant increase in lifestyle behavior (β =0.36, $p< .01$). Age, gender, marital status, and cancer stress were not found to be associated with changes in lifestyle.<br><br>Female gender was significantly associated with less positive changes in substance use (β= -0.21, $p < .05$). Age, marital status, cancer stress and benefit finding were not found to be associated with changes in substance use.	
Harper et al. (2007) [158] USA		216 Cancer survivors who completed treatment at least 6 months but no more than 10 years prior to participation.<br><br>33% breast, 17% lung, 10% thyroid, 9% lymphoma, 6% colorectal , 6% ovarian, 3% prostate, 2% melanoma and 15% other<br><br>81% female<br><br>Mean (SD) age 49.9 (12.1) years<br><br>Mean (SD) time since diagnosis 3.1 (2.3) years	*Intra-individual:* Optimism (Life Orientation Test-Revised), cancer-related distress (Impact of Event Scale), traumatic stressor response (two questions about whether the cancer experience constituted a traumatic stressor).<br><br>*Inter-individual:* Social support (Duke-UNC Functional Social Support Questionnaire), and social constraints (single item based on Lepore's (1997) measure.).<br><br>*Socio-demographic:* Age, marital status, race, and education.	Lifestyle change was assessed by asking participants whether the health behavior increased, decreased or had not changed since diagnosis. A composite index of positive change since diagnosis and treatment was created for the health behaviors eating a healthy diet and engaging in PA, in which the number of behaviors for which a respondent indicated the frequency had increased since diagnosis was summed.	Dispositional optimism (β = 0.04; $p< .01$), social support (β = 0.02, $p < .05$), and cancer-related intrusions (β= 0.03, $p< .05$) were a significant predictor of positive physical behavior change. Age, race, education, and marital status, social constraints, and cancer-related avoidance, traumatic stressor response were unrelated to positive health behavior changes.	

Keaver et al. (2020)[32] USA	Cross-sectional	315 female *breast cancer* survivors Mean (SD) time since diagnosis 5.3 (5.7) years Mean (SD) age at diagnosis 47.3 (9.6) years Mean (SD) BMI 26.3 (5.9) kg/m^2	*Socio-demographic:* Educational level, race/ethnicity, and age	PA and diet were assessed by questions adapted from an online Nutrition and Activity Quiz by the American Cancer Society. Change was assessed by asking whether they spend more, less or the same amount of time in PA after cancer diagnosis or treatment, and whether they eat more, less or the same amount of fruit, vegetables, whole grains, low-fat dairy, limited red and processed meats, sweets, fried foods, salty foods, and solid fats. Making positive changes was defined as 'yes' if participants reported making positive changes on any of the nutrition and PA behaviors and 'no' otherwise.	*Socio-demographic:* Participants with a higher level of education were more likely to make positive changes in PA or diet. High school vs. grades 0-12: OR 3.7 (95% CI: 1.0 – 13.5). College graduate or higher vs. grades 0-12: OR 2.9 (95% CI: 1.0 – 9.0). Age and race/ethnicity did not significantly predict making positive changes in PA.
Trudel-Fitzgerald et al. (2018)[63] USA	Longitudinal observational study	145 women with *colorectal* cancer<br><br>Mean age was 66.4 years old (SD=8.6; range 42–83 years)	Intra-individual: Anxiety (Crown-Crisp Index), depression (Mental Health Index)	Lifestyle score included five behavior-related factors: physical activity, diet, BMI, alcohol and tobacco consumption measured pre-diagnosis, within 4 years after diagnosis and every 4 years thereafter	Every 1-SD increase in anxiety symptoms was related to 63% greater odds of reporting an unhealthy lifestyle at the study termination (odds ratios: OR=1.63, CI=1.06–2.52, p=0.03).<br><br>Although the association did not reach statistical significance, women with higher vs. lower depression symptoms had 51% reduced odds (OR=0.49, CI=0.20–1.23, p=0.13) of having an unhealthy lifestyle at the end of follow-up. Every SD increase in depression symptoms was also related to a non-significant 19% reduced odds (OR=0.81, CI=0.61–1.07, p=0.14) of reporting an unhealthy lifestyle later on.
