# Supplementary material for: Psychosocial Determinants of Lifestyle Change after a Cancer Diagnosis: A Systematic Review of the Literature
Source: Cancers (Basel). 2022 Apr 16;14(8):2026. doi: 10.3390/cancers14082026 (PMC9032592; doi:10.3390/cancers14082026)
Supplement: Supplementary file 1 [file cancers-14-02026-s001.zip › Table S6_MH.pdf]

Table S6. Overview of quantitative studies assessing psychosocial determinants of changes in sun protection behavior among cancer survivors (n=2).

First author (year) Country	Study design	Sample Characteristics (at baseline)	Psychosocial variables Type(s); Assessed at; Assessed with	Lifestyle Assessment instruments Baseline; Change (period)	Findings	Comments
Hall et al. (2019) [31] USA	Cross-sectional observational study	258 early stage cancer survivors who had completed primary treatment<br><br>27% *breast*, 21% *hematologic*, 11% *gynecologic*, 9% *GI*, 8% *genitourinary*, 6% *head and neck*, 6% *melanoma*, 4% *thoracic* and 2% other<br><br>54% 60 years or older<br><br>64% female	*Intra-individual:* Fear of cancer recurrence (Assessment of Survivor Concerns), emotional distress<br><br>To assess emotional distress participants were asked to rate the extend to which they have felt nervous or worried and sad or depressed over the past month.	Changes in sunscreen use was assessed by asking participants how their sunscreen use had changed compared to before their diagnosis and responses were categorized in to decreased, no change or improved.<br><br>29% increased sunscreen use, 1% decreased sunscreen use, and 69% reported no change in sunscreen use following diagnosis.	There was no significant association between fear of cancer recurrence, and emotional distress with sunscreen use.	
Humpel et al. (2007) [78] Australia	Cross-sectional	113 cancer survivors<br><br>41.6% *breast*, 14.9% *melanoma*, 10.9% *cervical*, 6.9% *colon/rectal*, 5.9% *ovarian*, and 19.8% other<br><br>86.7% female<br><br>49.6% older than 55 years	*Socio-demographic:* Marital status, employment status, age and educational level	Participants were asked whether the cancer diagnosis influenced changes in sun protection behavior and when these changes occurred.<br><br>76.7% reported more sun-safe behaviors after diagnosis.	There were no significant differences in sun protection changes depending on education level, marital status, and employment status. Being older than 55 was significantly associated with increased sun-safe behavior ($p < .05$).	
