# Supplementary material for: Psychosocial Determinants of Lifestyle Change after a Cancer Diagnosis: A Systematic Review of the Literature
Source: Cancers (Basel). 2022 Apr 16;14(8):2026. doi: 10.3390/cancers14082026 (PMC9032592; doi:10.3390/cancers14082026)
Supplement: Supplementary file 1 [file cancers-14-02026-s001.zip › Table S7_MH.pdf]

Table S7. Overview included qualitative studies on psychosocial determinants of exercise/ physical activity only (n=26).

First author, (year), Country	Study design	Sample characteristics	Lifestyle change	Findings on psychosocial determinants
Adams et al. (2021)[109]<br><br>USA	*Focus groups* were conducted prior to implementation of an exercise program.<br><br>Focus groups were composed exclusively of interventionists (2 groups) or rural female cancer survivors (3 groups). Only results for cancer survivors are presented.	N=19 rural female cancer survivors<br><br>Mean age = 61.7 ± 10.9 years<br><br>Mean 33.2 +/- 21.5 months after cancer diagnosis.	Period not specified	*Barriers:*<br>Both physical and psychological cancer specific adverse effects (loss of balance, fatigue, decreased strength, and "brain fog", and trouble coping with exercise limitations after treatment), lack of support, lack of knowledge, perceived negative aspects of exercise, cost, lack of resources, motivation, inconvenience, lack of program flexibility, time, weather, safety.<br><br>*Facilitators:*<br>knowledge, ease of access, resources, (e.g., advertising exercise program through media outlets, end of treatment exercise discussions with medical team), cost, options (e.g., range of exercise offerings based on fitness level, multiple time options), organized (e.g., small groups, instructors), and fun.
Adamsen et al. (2017)[141]<br><br>Denmark	*Longitudinal semi-structured, open-ended interviews* embedded in a pilot randomized controlled trial, conducted at baseline and directly after a 12-week intervention during adjuvant chemotherapy.<br><br>Wait-list control group design.	N=33 patients with breast (n=25) or colon (n=8) cancer<br><br>Median age 49 years<br><br>N=84,8 % female (n=28)<br><br>All participants received adjuvant chemotherapy during the study period.<br><br>All had physical activity levels below national guidelines	During 12-week intervention: either a hospital-based, supervised, high-intensity group exercise intervention or a home-based, low-intensity individual pedometer intervention.	*Barriers:*<br>Side effects of chemotherapy (fatigue, infections and achy joints and muscles)<br><br>*Facilitators:*<br>Consultation with the oncologist, when exercise during chemotherapy was advised and benefits were explained; feeling safe to exercise with support from professionals; perceived bodily, emotional (e.g., improvement of mood) and social benefits from exercise (feeling great after exercise despite side effects); support of healthcare professionals, peers, family, and friends (the coaches and camaraderie helped in maintaining participation despite side effects; building self-confidence/ believing in one's own abilities; being motivated to set and reach new goals/ having the willpower to reach new goals; perceived pride regarding acquired self-discipline; improvement of physical and emotional well-being, leading to reflect on prioritizing physical activity in the future; enjoyment of exercise; emotional and social benefits of being with others facing similar circumstances.
Avancini et al. (2020)[146]<br><br>Italy	*Semi-structured focus groups (n=5)*	N=18 patients with different cancer types, mainly breast (n=)6 and pancreas (n=6)<br><br>Mean age 55.2 (SD 10.3)<br><br>Mean BMI 23.7 (SD 3.0)<br><br>Mean time from diagnosis of 25.7 months	During oncological treatment	*Barriers:*<br>Treatment-related side effects, advanced disease, and medical procedures (e.g., ileostomy); Lack of motivation/ laziness; not being a physically active person; chemotherapy malaise, expressed especially with fatigue; Disease condition, medical treatments and risk of injury (e.g., central venous catheter impairing the perception to be able to de physically active); Lack of information regarding physical activity in the oncological setting; Weather, expenses, lack of time.<br><br>*Cues to action/ facilitators:*<br>Counseling and social support; initial advice of oncologists or other relevant figures; support from family and friends; goal setting; enjoyment/satisfaction of PA; having a pet; a targeted flexible physical activity program based on patients' characteristics; an available physical activity specialist for more self-confidence in performing physical activity; organizational aspects (suitable structures to facilitate an active lifestyle: outdoor facilities, distance to fitness center; receiving credible and suitable, detailed

				information, including the benefits and practical considerations to exercising.<br>*Perceived benefits:*<br>-Physical & physiological benefits: physical activity is beneficial for overall well-being and can counteract treatment related side-effects.<br>-Socio-psychological benefits:<br>Better mood, helps to fight depression and to have a different attitude in approaching cancer; feeling recharged and ready to face everything; organizes the day; increases perception of control, in the cancer context; helps to face cancer, also in the family context (being physically active together).
Backman et al. (2016)[136]<br><br>Sweden	*In-depth individual (n=13) and focus group (n=1) interviews*	N=16 women diagnosed with breast cancer receiving adjuvant chemotherapy treatment and participating in physical activity during chemotherapy treatment<br><br>Interview (n=13): Mean age 54 (36-70)<br><br>Focus groups (n=3): Mean age 64 (57-71)<br><br>The women were recruited from a physical activity randomized controlled intervention study(OptiTrain).	During adjuvant chemotherapy treatment	*Barriers:*<br>Symptoms and side effects such as fatigue, infections, dizziness, stomach problems, muscle ache and a generalized feeling of weakness; bodily decline and mentally and cognitive weakness that was hard to cope with; Other demands on time (working during treatment, travel time to the gym or demands from family life).<br><br>*Facilitators:*<br>Feeling the benefits from PA; Physical activity as a way of contributing to self-appreciation and making oneself feel better; PA just made them feel good; Physical activity providing purpose (a new start to life, having a goal for the day or for the long term, providing a reason to leave the home and to have something specific to do); feeling less emotionally affected; positive experience; perceiving PA as an important factor in maintaining health during the treatment period and thereafter; feeling of better fitness, shape and strength than before due to PA; Focus on health and wellbeing rather than on illness; experiencing functional improvements (having a stronger body, better balance and flexibility) and symptom improvements (less fatigue, dizziness and breathlessness, and feeling more alert cognitively and more in harmony with their whole body); PA contributed to not wanting to slide into self-pity, but rather to look forward; social support (continuity with the PA team and the positive reinforcement they received, also from fellow patients, family and at work); having someone to talk to and get advice from in a controlled/organized, supportive, relaxed and safe environment with fellow-patients; PA gave a sense of trust in their own body and their own capacity, and a confirmation that their body and mind was strong enough to exercise despite illness and treatment; PA in itself made them stronger, mentally, which gave them a sense of pride and belief in themselves; contributed to structuring the day and filling their time with regular commitments every week; Having scheduled appointments, the continuity of meeting the same PA team, a comforting and safe exercise environment, easy access to making, rescheduling and canceling appointments on your own online via the internet, and that the gym was fairly close and easy to access; feelings of empowerment and independence/ giving strength and motivation to focus on health rather than illness; Increased knowledge about the impact of PA on the body in general and after a diagnosis of breast cancer and treatment was described; knowing that PA could positively influence symptoms such as fatigue and pain; Strengthening influence of being able to accomplish and control PA themselves.
Banerjee et al. (2019)[97]<br><br>UK	*Focus groups (n=3)*	N=14 patients with bladder cancer	(PA) during the exercise program + postoperative exercise	*Barriers:*<br>-Barriers to postoperative exercise:<br>Difficulties exercising outside because of feeling the cold more after surgery; Concerns about managing their stoma

		Mean age ±SD 72.3±6.0 years (range 64-82)<br><br>BMI 27.0 +/- 4.8<br><br>Female 1 of 14<br><br>Recruited within 6 months of participating in a high intensity aerobic interval preoperative exercise program before radical cystectomy.<br><br>None of the patients recruited to the preoperative exercise study were meeting current physical activity guidelines of a minimum of 150 min of moderate intensity physical activity per week		bag during exercise; A lack of knowledge on what is perceived to be safe and realistic.<br><br>*Facilitators:*<br>-Key motivational factors for engaging in exercise prehabiliation: social support, previous exercise experience (and the health benefits of exercise) and objective measures of progression.<br>-Motivational factors for maintaining participation: Receiving appropriate supervision, objective measures of fitness improvements and support from health care practitioners and family members; Prior exercise experience gave the confidence to push themselves during the exercise sessions; Monitoring exercise intensity, especially when improvements in fitness were noticed; Encouragement from family members (some accompanied participants and even took part, others encouraged participants to undertake exercise at home);<br>Supervised exercise provision, support received and relationships built with exercise instructors.<br>-Motivation for postoperative self-directed exercise and physical activity: receiving appropriate supervision.<br>*Perceived benefits of participation:*<br>-Physical benefits: physical fitness and strength improvements, recover faster, experiencing weight loss.<br>-Social benefits: the opportunity to spend time with other patients, share experiences and benefit from a support system.<br>-Psychological benefits: enhanced mental preparation prior to surgery.<br>Being involved in preparing for their operation gave a sense of control and ability to take an active role in their care.
Brunet et al. (2013)[119]<br><br>Canada	*Semi-structured, in-depth interviews*	N=9 female *breast cancer survivors*; post-treatment for a first diagnosis of breast cancer<br><br>Mean age: 55.3 years<br><br>78% had completed primary treatment within 5 years of the study (range 2–31 years)	Maintain participation in physical activity/ a self-directed physical activity program	*Barriers:*<br>Physical (cancer-related physical symptoms: mobility limitations, fatigue, lack of energy, and pain); environmental/ organizational: bad weather, costs, safety issues, lack of equipment/inadequate facilities, lack of knowledge/information, time constraints), and psychosocial: lack of motivation, low social support/ lack of companionship, low confidence/ skill<br><br>*Facilitators/motivators:*<br>Physical (weight management, health improvement or maintenance, improving fitness/strength feeling energized) and psychosocial: improve body image, experience enjoyment, social support, positive emotions, moral obligation, attitudes, feelings of fulfilment/ accomplishment/pride; habit, social norms.
Leak Bryant et al. (2017)[110]<br><br>USA	*Semi-structured interviews*	N= 6 adults with acute leukemia who recently completed an inpatient exercise intervention during induction therapy.<br><br>Mean age 55 years. Range (35–67) years<br><br>17% Female (n=1)<br><br>44% were considered overweight	During intervention, an individualized, mixed-modality exercise program supervised by exercise sport science specialists.	*Barriers:*<br>Anxiety, and physical symptoms, such as aches and pains.<br><br>*Facilitators:*<br>Social connectedness with the exercise trainers; the exercise routine (structure and consistency) motivated.<br>-Physical benefits: decreased aches and pains, lower fatigue.<br>-Psychological benefits: feeling better; less anxiety.
Coon et al. (2004)[111]	*Longitudinal in-depth interviews*	N=21 multiple myeloma patients	During intervention,	Complex interactions between participants' beliefs and social context and experience appraisal influenced their ability to adhere.

USA	The first interviews took place approximately 10–12 weeks after participants enrolled in the exercise program while undergoing two courses of induction chemotherapy.<br><br>Confirmatory telephone interviews took place after participants returned home following recovery from their first transplant and no longer were participating in the exercise study.	42.8% female (n=9)<br><br>Mean age 52 (Range 36–70)<br><br>All enrolled in the exercise arm of a randomized trial of prophylactic epoetin alfa with or without exercise.	an exercise program in the context of an aggressive tandem peripheral stem cell transplant protocol.	*Barriers:*<br>Chemotherapy side effects challenged beliefs about the difficulty of the exercise program and self-concept about the ability to exercise.<br><br>*Facilitators:*<br>Social support (walking with a group of friends); beliefs about the benefits and risks of exercise (e.g., the believe that exercise would be good for them while they were going through treatment); the belief that exercise can help mood, the confirmation of the belief through experience, and expression of the need for a personal feeling of normalcy as part of her self-concept; the social context of being in the exercise study, along with the belief that the exercises would be easy; an ethical philosophy of keeping her word, provided a congruent context for successful participation in the program.
Frensham et al. (2018)[129]<br><br>Australia	*Semi-structured face-to-face tinterviews*	N=10 cancer survivors living in rural areas of South Australia who participated in a 12-week computer-delivered walking-based intervention.<br><br>Mean age 74.0 (SD 5.5) Range 60-78<br><br>50% female (n=5)<br><br>Cancer type:<br>Beast n=5; Prostate n=4; Face n=1<br><br>Participants were eligible if they were insufficiently active.	During the 12-week intervention and up to three months after the three-month follow-up period.	Physical, psychological, social, environmental, and organizational motivators and barriers.<br><br>*Barriers:*<br>-Physical limitations: Fatigue, Pain, Chronic illness.<br>-Psychosocial barriers: Social isolation; Lack of motivation/ encouragement; difficulties in maintaining their motivation to walk after the program had finished; attitudinal and emotional factors: not feeling good enough/ down.<br>-Environmental barriers: poor weather conditions, poor walking environment (unenjoyable walking in rural areas due to lack of excitement and social opportunities), safety issues, geographical isolation (social isolation of living out of towns).<br>-Organizational barriers: time constraints, competing priorities, lack of external encouragement and organization post-program.<br><br>*Facilitators/Motivators:*<br>-Physical/psychological Benefits: Increased fitness; Weight loss; Feeling better; Improved body image.<br>-Social support: Obligation to research team; Appreciated support and encouragement from research team (and feelings of responsibility for their health due to the encouragement and support received); Enjoyed connecting with others, receiving support from their family and friends (e.g., partners joining them on their walks).<br>-Website factors: Tailored step goals (set by researchers); Graphic visualization of progress on website (the step log and graph on the STRIDE website were powerful self-monitoring and self-motivating tools to keep the routine).<br>-High barrier self-efficacy in the face of barriers
Götte et al. (2014)[147]<br><br>Germany	*Cross-sectional; semi-structured individual interviews* during in-patient treatment	N= 40 pediatric cancer patients during intensive treatment<br><br>Various cancer types; most leukemia (n=13) & bone tumor (n=18)<br><br>Mean age 13.2 (SD 4.1) range 4-20 years<br><br>47% (N=19) female	Children and adolescents with cancer were interviewed during the phase of acute medical treatment and received chemotherapy according to their disease specific study protocol.	The local exercise program was desired and valued as essential for engaging in exercise during in-patient stays.<br><br>*Barriers:*<br>-Physical aspects: side effects of oncological treatment (nausea, physical fatigue, gastrointestinal problems, pain), feeling too weak to exercise because of powerless legs and lack of cardiorespiratory fitness, limitations of loading the tumor-affected extremities; specific movement problems (e.g., dizziness, balance problems), aplasia.<br>-Psychological aspects: lack of energy; no motivation, bad mood, prefer to stay in bed (e.g., to retreat or to distract),

		Frequency of participation varied within the interviewed patients. Number of interventions was ranging between 0 and 50 (mean 14.3+/-9.2) times) in the patient collective. The majority of exercise interventions took place at the cancer ward (73%) while going to the hospital's gym was less frequent.	All patients had the possibility to participate in a supervised inpatient exercise program implemented for hospital stays that offered, for example, strength training, sensorimotor training, relaxation training, ball games, and active console games.	concerns about overexertion and injuries, grief about inability to engage in normal sport activities.<br>-Organizational restraints: Lack of time, no ideas about feasible activities; in-patient: little space, infusion stand; home stays: lack of sport equipment, no friends to play with, bad weather.<br><br>*Motivations/ facilitators:*<br>-Physical fitness: Increase/maintain muscle mass and cardiorespiratory fitness, maintain self-reliance, attain easier reintegration after treatment, coping with chemotherapy, positive short-time effects (get agile, improve sleep), lose weight/body styling.<br>-Mental well-being: Pastime, improve mood, mental distraction (from disease), feeling normal.<br>-Facilitating aspects: Getting asked to exercise, accessibility of playing fields and sport equipment, existence of exercise program.
Grimmett et al. (2020)[98]<br><br>UK	Cross- sectional qualitative study: *Semi- structured interviews*<br><br>*Aim:* to explore the experiences of participants in order to characterise those who have and have not successfully sustained increases in PA following participation in a PA intervention after a diagnosis of gastrointestinal (GI) cancer, and identify barriers and facilitators of this behaviour.	N=27<br><br>N=15 male (56%)<br><br>Mean age= 66.3 years (range 41–79)<br><br>Gastrointestinal cancer (colorectal, oesophageal or stomach cancer) survivors who had completed one of four interventions designed to encourage PA participation more than 6 months before participating in the current study.	From participation in a PA intervention to more than 6 months later	*Barriers:*<br>Disease limits activities (e.g., due to stoma, other comorbidities including atritic conditions, back problems, asthma), particularly in cold weather; avoiding activities due to issues of body esteem and concerns about colostomy bag leakage; avoiding certain activities where toilet facilities were unavailable (bowel urgency), as well as adapting activities due to concerns about leaks or accidents; low mood and apathy; low motivation; lack of enjoyable physical activities; competing demands: responsibilities such as caring for family members, family commitments and work schedules; Ageing perceived as inhibiting physical activity<br><br>*Motivators/facilitators:*<br>Physical activity as essential in maintaining good health and well- being, avoiding illness and physical deterioration, PA to combat consequences of ageing (preserve fitness and mobility and slow physical decline); Enjoying the physical activities; feeling a positive impact on psychological well-being; PA as a distraction from concerns about health and anxieties; feeling empowered by taking exercise, using it as a vehicle to take control over their health following their cancer diagnosis and treatment; opportunity for social interaction during PA; avoiding/reducing isolation; opportunity to develop and maintain meaningful and long-lasting friendships with those they exercised with; one-to-one specialised support provided during involvement in a PA promotion programme helped individuals find activities they enjoyed and were appropriate for them; use of self-monitoring techniques (e.g., pedometer) motivated; receiving feedback on behavior (number of steps on pedometer).
Hardcastle et al. (2019)[130]<br><br>Australia	*Semi-structured interviews* as part of a larger study that involved using five commercially available wearable activity trackers (Fitbit Alta, Garmin Vivofit 2, Garmin Vivosmart, Polar loop 2 and Polar A300) to assess the acceptability of and	N=16 individuals with breast (n = 8), endometrial (n =4) or colorectal cancer (n =4).<br><br>Mean age = 60 years (SD 12)<br><br>N= 87.5% (N=14) women<br><br>Patients completed active treatment for cancer within the	During study period of larger study. Participants trialled a minimum of two devices, each device for 2 weeks, followed by a 1-week washout period between devices.	*Barriers:*<br>Lack of motivation, older age, health status, and lack of facilities or exercise programs. Insufficient knowledge of guidelines and health benefits: Survivors' knowledge of physical activity guidelines was limited and they did not often recall their oncologists making specific recommendations concerning physical activity; Not prioritizing physical activity.<br><br>*Facilitators:*<br>Support from the treating oncology team. The oncologists' seal of approval was important for encouraging participation; The way in which lifestyle information is

	utility of wearable technology to increase physical activity in non-metropolitan cancer survivors.	preceding 5 years and were insufficiently physically active (i.e. not meeting the recommended 150-min of moderate to vigorous intensity physical activity per week.<br><br>Mean months since end of treatment 22.9 (SD = 15.7)		communicated by clinicians was deemed important: confident, optimistic communication style.<br>Survivors' referred to the desire for external accountability and monitoring in order to successfully change physical activity.
Hatlevoll et al. (2021)[143]<br><br>Norway	*Longitudinal individual, semi-structured interviews* at different time-points during adjuvant chemotherapy among colorectal cancer patients who participated in physical exercise interventions during treatment.<br><br>Interviews were performed at the beginning (n=13), during (n=10, after 12 weeks), and immediately after the intervention period (n=6, after 24 weeks).	N=15 colorectal cancer patients participating in two intervention studies with individually tailored and supervised combinations of endurance, resistance, and balance exercises during adjuvant chemotherapy.<br><br>Mean age = 65 (43-80)<br><br>46.7% female (n=7)	During intervention, which lasted throughout the treatment period (ie, 12– 24 weeks)	The exercise program provided structure to life with cancer, motivation to exercise, training experiences, and effects of exercise.<br><br>*Barriers:*<br>Motivation was threatened by exercises they did not like or found boring, and when they felt fatigued: inner struggle between going to the gym and the desire to rest, especially at times when even simple activities of daily living was a struggle; physical fitness could vary within each chemotherapy cycle. Generally, exercising felt harder toward the end of the treatment period; intercurrent illness with infection.<br><br>*Facilitators:*<br>-Scheduled appointments gave structure to daily life and served as an external motivational factor. It helped to structure their day and gave them something to look forward to. To become isolated was a concern, and signing up for the study represented a good opportunity to avoid this. Advantages from getting out instead of sitting indoors doing nothing was another benefit. Having appointments helped them to get up and out when feeling depressed or tired.<br><br>-Motivations: keeping both body strength and flexibility; good for both physical and mental health, including increasing the efficacy of chemotherapy; inner motivation as demonstrated through a strong desire to exercise and not skip out; crucial external motivation for exercising both regularly and efficiently came through the appointments with the physiotherapist; earlier experience of symptoms decreasing during and after exercise; Supervision by a physiotherapist provided motivation to perform a little extra, and to complete all the exercises, even the boring ones. Guidance from a physiotherapist to adjust the exercising according to variations in their physical function also gave a sense of security.<br>-Training experiences: use of a heart rate monitor as a motivational factor for exercise.<br>-Effects of exercise: Experienced positive effects from exercising, both mentally and physically, contributed to inner motivation; Positive effect on mental health; reducing symptoms of depression, feeling of joy or happiness; losing weight; being able to keep in shape and keeping their strength, despite receiving chemotherapy, led to feelings of satisfaction.
Hefferon et al. (2013)[99]<br><br>UK	*Semi-structured interviews*	83 female breast cancer survivors who originally participated in a group-based structured exercise intervention after diagnosis. The interview was carried	From end of intervention to follow-up at 5-years post-diagnosis.	*Barriers:*<br>-Psychological barriers: lack of motivation, lack of 'willpower' or laziness; fears (dangers with engaging with activity such as fear of being attacked as well as slipping in poor conditions and injury); dislike of gym and its environment; not being the 'sporty type'.

		out at 5-year follow-up to the original randomized control trial.		-Physical barriers: the ageing process; cancer treatment and other physical co-morbidities; fatigue and weight gain.<br>-Contextual and environmental barriers: employment; traditional female care-giving roles; proximity/access to facilities; seasonal weather.
Humphreys et al. (2020)[100]<br><br>UK	*Focus groups and semi-structured face-to-face or telephone interviews.*<br><br>Two focus groups (n=3 and n =6) and 17 interviews were held	People affected by cancer (n=26) and professional stakeholders (n=14) (Results not reported)<br><br>Various cancer types, mostly breast cancer (n=12) and leukaemia (n= 5)<br><br>69% female (n=18)<br><br>Mean age = 53 years<br><br>Mean time since diagnosis 7 years<br><br>Participants were at varied stages in the cancer pathway; currently in treatment (n=6); completed treatment with confirmed remission (n=12); secondary/advanced cancer (n=8).	Unspecified	*Barriers:*<br>-Fear of making their health worse (uncertainty of their exercise capacity and limitations).<br>-Low confidence in their ability to exercise: the impact cancer treatment had on their mood and confidence.<br>-Cancer-related fatigue<br>-Lack of advice on how and when to exercise: No support from clinical teams; Lack of encouragement from health professionals.<br>-information saturation around diagnosis and the start of treatment and a limit to their ability to absorb the overwhelming amount of information provided. An exercise discussion was not a priority for discussion at that time.<br>-Patients identified a lack of support<br>-Difficulty attaining information<br>-The financial implications of a cancer diagnosis must be considered (financial uncertainty); expressed a willingness to make a modest contribution to an exercise intervention.<br><br>*Facilitators:*<br>-Health benefits of exercise (improved mental wellbeing)<br>-Becoming more physically active was seen as a mechanism to regain personal control of their body and of their lives more generally enabling self-sufficiency and retaining or restoring independence<br>-Enhancing recovery from cancer: fitness for treatment and promoted recovery.<br>-Social interaction: social/peer support<br>-Accessibility & affordability: participants felt paying for a service will increase commitment.<br>-Tailored support: Exercise must be tailored to the individual: initial assessment to identify individual needs and goals, followed by a choice of exercise options.<br>-Learning the art of pacing of exercise.<br>-Trained exercise professionals
Husebø et al. (2015)[144]<br><br>Norway	*Focus group interviews (N=5)* following the completion of participation in a home-based exercise programme tested in a randomized, controlled trial for women with operable breast cancer	N=27 women with early-stage breast cancer<br><br>Mean age 52 years (SD +/- 9, range 34–69)	During a home-based exercise intervention that combined strength and aerobic training to be performed throughout chemotherapy.	*Barriers:*<br>-Side effects of breast cancer treatment, such as nausea and fatigue, mostly after multiple chemotherapy cycles; hospital admissions due to treatment complications<br>-Changed body image due to treatment (mastectomy), and inconvenience and worries due the use of a prosthesis.<br>-Other valued activities competing with exercise (balancing between time to exercise, work, and social events)<br>-Information on exercise as a rare topic during clinic appointments unless when the patients brought it up themselves or if the cancer nurse herself was in to exercise.<br>-Not knowing how to perform the exercise led to doubt over the exercises' effectiveness, and consequently reduced effort, which was perceived as a vicious cycle of demotivation.<br><br>*Facilitators:*<br>-Restoring and maintaining normality in daily life motivates exercise.<br>-Exercise organized daily life.<br>-Having the feeling of being in control with exercise<br>-Constructive, informative support from health care professionals to manage the side effects while exercising<br>-Emotional and practical social support (from family and friends, neighbours and colleagues).<br>-Motivational telephone calls during intervention

				-Positive beliefs about efficacy and outcomes: high expectations of positive effects of the exercise. They were certain that exercising would do them good, help them towards a speedy recovery and prevent the cancer from reoccurring.<br>-Viewing exercise as one's own effort for getting better<br>-Commitment and selfdiscipline<br>-Positive expectations on mastery and goal achievement<br>-Self-monitoring; keeping an exercise diary helped to establish an exercise routine and set exercise goals.<br>-Experienced benefits of exercise: exercise routine helps focus on positive perspectives of life; gave them a break from the problems of cancer; helps process negative thoughts and feelings.
Martin et al. (2021)[145]<br><br>France	*Longitudinal; 2 x 2 focus groups:* Two independent groups of patients were interviewed 2 consecutive times, before and after participation in a 2-week mHealth group experience.	9 breast cancer patients<br><br>100% female<br><br>Median age at diagnosis 47 (Range 29-60) years<br><br>Did not meet the World Health Organization recommendations for physical activity (ie, 150 or 75 minutes per week of moderate or vigorous activity or equivalent combinations<br><br>All completed breast cancer primary treatment between 3 and 18 months before the first group meeting.	During intervention (2 weeks of an mHealth group challenge) and after diagnosis in general	<u>Physical activity after breast cancer:</u><br>*Barriers:*<br>-Physical barriers: late effects of cancer treatment.<br>-Psychological barriers: Lack of motivation, lack of habit, counterintuitive approach, having stopped working out during treatment, fear of being pushed too much, or practicing alone.<br>-Organizational barriers: lack of time, resuming work and/or working full time, and family commitments<br>*Facilitators:*<br>-Physical factors such as perceptions of physical benefits and previous practice<br>-Psychological factors such as motivation increased by provider recommendations<br>-Social factors such as group practice<br>-Organizational factors including preplanning physical activity sessions.<br><br><u>Use of mHealth:</u><br>*Barriers:*<br>-Not being familiar with or interested in mHealth.<br>-Lack of information/explanations about mHealth.<br>*Facilitator:*<br>-Ability to know how many steps a day and track the activity they do<br>-Motivation driven by the ability to track activity<br>-use of mHealth promotes habit
Maxwell-Smith (2017)[131]<br><br>Australia	*Semi-structured interviews*	N = 24 Colorectal cancer survivors who completed active treatment in the past 2 years and had existing morbidities that increased their cardiovascular risk.<br><br>54,2% female (n=13)<br><br>Mean age 69.4 (SD 4.2) Range 63-77	Unspecified	*Barriers:*<br>-Psychological barriers: not the sporty type; too much effort/lack of willpower (lack of effort and discipline); and competing priorities/lack of time (work, family, or social commitments);<br>-Environmental barriers: weather; lack of social support;<br>-Insufficient knowledge of physical activity guidelines; felt that the guidelines were not applicable to them; inaccurate beliefs that they were sufficiently physically active/ overestimated their physical activity level;<br>-Lack of practitioner support: medical surveillance (recommendation of medication rather than lifestyle change); insufficient physical activity advice from medical professionals<br>-Energy/age barriers.<br><br>-Regular checkups to monitor cardiovascular risk replaced the need for healthy lifestyle changes.<br><br>*Facilitators:*<br>-practitioner support, including a reliance on practitioners to recommend lifestyle change.

				-Social support: being more physically active when exercising socially.
Mazzoni et al. (2019)[137]<br><br>Sweden	*Mixed-methods concurrent triangulation study,* using semi-structured interviews (n=18) and a questionnaire (n=229)<br><br>The present study was part of a multicentre randomized controlled trial	Patients with breast, colorectal or prostate cancer who completed or dropped out of a 6-month exercise programme during oncological treatment.<br><br>Questionnaire participants:<br>Mean age: 59 (SD 12)<br>78% female (n=178)<br>76% breast; 4% colorectal; 20% prostate cancer<br><br>Interview participants:<br>Mean age: 63 (SD 11)<br>Female 50% (n=9)<br>44% breast; 17% colorectal; 39% prostate cancer	During the intervention (low-to-moderate versus high-intensity exercise with additional behaviour change support)	*Barriers:*<br>-Treatment side effects: Cognitive impairments; Changes in appearance (e.g. hair loss); urinary symptoms or bowel disturbances, fear of infection and injury<br>-Fear of movement<br>-Lack of enjoyment in exercising<br><br>*Facilitators:*<br>-Increased motivation to exercise through the exercise programme<br>-Experienced physical and psychological health benefits from the exercise programme as incentives to perform exercise: feeling more alert, improved mood and well-being, increased vitality, improved muscular strength and fitness, better quality of sleep. Experienced benefits gave a feeling of self-control and competence, which were important incentives to exercise.<br>-Increased awareness and knowledge of exercise and its benefits.<br>-Learning new skills, e.g, use of selfregulatory tools such as fitness tests, goal-setting and exercise planning.<br>-The participants could see their progress and improvement, which increased their motivation and confidence in their ability to exercise.<br>-Exercising in a group with other persons diagnosed with cancer; sharing their experiences about how to deal with treatment side effects, their feelings and concerns about the disease, and tips and advice on how to exercise; group cohesiveness gave a feeling of security and a sense of improved mental strength and increased exercise motivation and enjoyment.<br>-Strong sense of commitment: loyalty to the coaches and the group.<br>-Structure and social support provided by the coaches and training sessions<br>-Scheduled training sessions provided structure into everyday lives and was seen as a distraction from the disease.<br>-Exercising in a public gym helped them to feel normal and healthy.<br>-Being supervised by experienced coaches (feeling of safety, guidance and positive feedback).<br>-Social support from family and friends (e.g. exercising together and being encouraged)<br>-Managing to exercise more than expected, increased motivation and confidence.<br>-For supervised resistance training, the most valued specific behavior change techniques were: social support from coaches, structuring the physical environment with scheduled sessions, self-monitoring with resistance training log and feedback based on heart rate monitor and fitness tests.<br>-For homebased endurance training, the most valued specific behavior change techniques were: social support from coaches, feedback with the use of heart rate monitor and cardiopulmonary exercise tests.
Missel et al. (2015)[142]<br><br>Denmark	*Qualitative longitudinal feasibility study*: Semi-structured interviews at three time points along the treatment and exercise intervention trajectory	N=19 non-small cell lung cancer patients who enrolled in an exercise intervention 2 weeks post-surgery participated in qualitative interviews at three time points: the day after surgery (n=19),	During intervention	*Barriers:*<br>-Side effects of chemotherapy.<br><br>*Facilitators:*<br>-Sense of security of having professionals (physiotherapists and nurses) present who were knowledgeable about their illness and exercise.

	This qualitative component formed part of a randomized controlled trial comparing the efficacy of early initiated postoperative exercise (initiated as early as 2 weeks after surgery) with the effect of exercise initiated 14 weeks after surgery (usual care).	7 weeks post-surgery(n=16), and 4 months post-surgery(n=14).<br><br>Mean age = 63 years (Range 48–75)<br><br>42% female		-Experienced physical and emotional benefits, including improved breathing, increased well-being, better mood, and regaining vitality and energy level, which increased faith in the future.<br>-Experienced social support and benefits from group training with others in a similar circumstance, including a sense of community/ belonging.<br>-Music<br>-Increased knowledge about exercise<br>-Using heart rate monitoring belts<br>-Self-challenge
Rabin (2017)[112]<br><br>USA	Descriptive analyses on physical activity barrier measures after completion of a 12-week home-based physical activity and meditation intervention immediately (Intervention, n=19) or after a 12-week delay (Control group, n=16).	35 young adult cancer survivors, participating in a randomized controlled pilot study.<br><br>Age 18–39, diagnosed in the past 10 years, completed treatment, and not engaging in regular physical activity or a structured relaxation activity.<br><br>82.9% female (n=29)	During intervention	*Barriers:*<br><br>Schedule-related barriers<br>-Competing family or work demands<br>-Lack of time<br>-Vacation schedule interfering<br>-Visitor in home<br>-Holiday plans interfering<br><br>Health-related barriers<br>-Illness or other health issue<br>-Pain<br>-Fatigue<br><br>Other type of barrier<br>-Weather<br>-Lack of motivation<br>-Emotional distress
Robinson et al (2016)[113]<br><br>USA	*Focus groups and individual follow-up interviews:*<br>One to two weeks after completing a triathlon, 11 breast cancer survivors who trained together participated in one of three focus groups. Five months post triathlon, six of the 11 women who participated in the focus groups participated in individual phone interviews.	11 female breast cancer survivors<br><br>Mean age 50.9 (SD 7.1) years.<br><br>The average years of survivorship were five years (3.58 SD).	During a 14-week individualized team triathlon training programme and exercise maintenance thereafter.	*Barriers:*<br>-Work<br>-Cold weather<br>-Difficulty with location of workouts<br><br>*Facilitators:*<br>-Team approach to exercise initiation/ being part of a team/ group support<br>-External motivation from coaches, health care providers, survivors from previous cohorts of the program, and familial encouragement<br>-Internal motivation for health behavior change since cancer diagnoses<br>-The trainers made training fun.<br>-Exercise as a way of taking control over the disease/ their own health<br>-Fear of recurrence<br>-Sharing the experience of being diagnosed with breast cancer and survivorship thereafter.<br>-Bonds and friendships developed during training.
Short et al. (2013)[132]<br><br>Australia	*Semi-structured telephone interviews*	N=8 post-treatment breast cancer survivors<br><br>Mean age 55 (Range 44-63)<br><br>Mean time since treatment 4 years (Range 3-6)	Not specified	*Barriers:*<br>-Lack of knowledge: being unaware of specific physical activity to optimise cancer recovery and/or prevent recurrence; not receiving specific physical activity advice about exercising post treatment by their treatment team.<br>-Lack of motivation<br>-Limitations in physical functioning, such as existing injuries<br>-Environmental factors, such as the location and timing.

				-Health-related issues, such as fatigue, arthritis, declines in physical functioning (relating to both age and cancer treatment), lymphoedema and pain.<br>-Not finding physical activity enjoyable<br>-Poor access to physical activity facilities.<br>-The cost of attending physical activity facilities.<br>-Lack of footpaths in local infrastructure and surrounding area<br><br>*Facilitators:*<br>-Negative reinforcers facilitating exercise, such as feeling guilty for not exercising.<br>-Self-efficacy: confidence to engage in some form of regular physical activity was generally high in terms of skills<br>-Positive outcome expectations: enjoyment, increased energy, feeling good mentally and maintaining a healthy weight, improvements in sleep, a sense of achievement, preventing cancer coming back, keeping bones strong, preventing chronic disease and fighting the ageing process.<br>-Immediate benefits of physical activity (e.g., being able to sleep better, having more energy)<br>-Goal setting and monitoring performance, increased enjoyment and resulted in a feeling of achievement.<br>-Intrinsic rewards, such as feeling good after meeting challenges<br>-Social support (having someone to exercise with, social rewards of participating in physical activity with others, encouragement or having someone to "answer to").<br>-Pleasant PA environment in local infrastructure and surrounding area
Sun et al. (2020)[114]<br><br>USA	Qualitative analyses of physical therapy/ occupational therapy baseline geriatric/ functional assessment and intervention sessions notes (N=34 dyads) during a perioperative physical activity intervention.	34 dyads of patients diagnosed with lung (n=18) or gastrointestinal (colorectal, gastric, pancreas, liver; n=16) cancers and their family caregivers.<br><br>Median age Lung:<br>Patients: 74<br>Family caregivers: 71<br><br>Median age gastrointestinal:<br>Patients: 68<br>Family caregivers: 67<br><br>41% of patients female	During intervention; perioperative physical activity.	*Barriers:*<br>-Comorbid health conditions (surgical procedures, injuries)<br>-Physical symptoms (pain, fatigue/general weakness, nausea, dizziness, sleep disturbance)<br>-Functional limitations (difficulties with daily living, gait and balance issues, history of falls)<br>-Emotional well-being: anxiety, feeling overwhelmed psychological distress, stress, worries about postoperative recovery and quality of life)<br>-Other roles and responsibilities (primary caregiver for their spouse, full-time employment, other caregiving responsibilities)<br>-Unexpected life events (multiple medical doctor appointments in the perioperative setting, car accidents, additional medical issues, unanticipated long distance travels)<br>-Lack of time: busy daily schedules<br>-Lack of motivation: preference for a sedentary lifestyle/ for leisure activities, disinterest, dislike of structured programs, laziness<br>-Lack of personal history of physical activity engagement<br>-Environment/weather (rain, hot days, hills, uneven walking surface)<br><br>*Facilitators:*<br>-Physical activity as part of daily routine (merging of leisurely physical activity and social interactions, such walking with friends)<br>-Knowing physical activity can reduce stress/ using coping strategies to counter anxiety and stress. Positive coping strategies: church/faith communities, prayers, social activities and interactions, distraction activities, use of mind-body approaches (i.e. meditation) as helpful with managing anxiety and stress, so that they could focus on physical activity engagement.<br>-Setting goals for motivation

				-Social/ family support: encouragement to walk and participate in the intervention together.<br>-Experiencing benefits from walking: beneficial for physical symptoms and anxiety, helped them to relax, reduce stress and anxiety.
Webb et al. (2019)[101]<br><br>UK	*Mixed-methods: 181 questionnaires and 17 semi-structured telephone interviews* conducted 13-14 weeks after receipt of the intervention.<br><br>Part of a waiting list randomized controlled trial assessing the effectiveness of a print-based intervention supported by Internet tools, to improve physical activity levels in cancer survivors.	Survivors of various cancer types	During intervention	Questionnaires:<br>-Use of printed intervention components was positively correlated with physical activity improvement.<br>-Neither use of the Internet tools nor the perceived usefulness of e-newsletters were correlated with physical activity improvement.<br><br>Interviews:<br>*Barriers:*<br>-Experiencing consequences of cancer treatment, focus on managing their energy to get through their day, with no energy to be physically active.<br>-Lack of motivation<br>-Low mood<br>-Dealing with cancer was enough with no room for anything else<br>-Being inactive before diagnosis<br><br>*Facilitators:*<br>-Motivation<br>-Confidence<br>-Use of a monitoring tools (Wall Chart) to see progress at a glance as a visible reminder and a source of encouragement.<br>-Information on simple ways to become more active<br>-Action planning<br>-Goal setting<br>-Helping physical and mental recovery<br>-Feeling the benefits of physical activity in improving side effects of cancer and treatment, such as fatigue, physical function, mental well-being and weight gain.<br>-Raising the importance of physical activity in relation to cancer<br>-Motivated by a desire to get back to normal<br>-'Fitness' being part of self-identity
Wurz et al (2015)[120]<br><br>Canada	*Semi-structured interviews*	Seven women who completed treatment for breast cancer were interviewed during the first and last week of the program.<br><br>Mean age 55.3 years	During intervention, an 8-week group-based physical activity program	*Barriers:*<br>Situational<br>-Institutional factors: (feeling overwhelmed by) Competing roles and responsibilities, such as scheduling conflicts (such as work and timing of classes) as well as having other commitments and responsibilities<br>-Community factors: Distance of center; Traffic<br>Internal<br>-Cancer-specific limitations related to cancer and treatment, such as side effects: being sick, physical limitations caused by surgery, negative effects of being sedentary during treatment (loss of muscle mass).<br>-Having flu- or cold-like symptoms made them avoid the class to protect other participants with weakened immune systems while undergoing treatment.<br>-Difficulty and embarrassment engaging in physical activity in the community due to changes to their bodies as a result of the treatments. (not being comfortable wearing a prosthetic)<br><br>*Facilitators/ Motives*<br>Situational<br>-Interpersonal: Receiving social support and encouragement from other program participants, camaraderie and friendships gained; social networking opportunities; Being around similar others affected by cancer

	-Internal: feeling a sense of personal fulfillment (as a result of realizing they were capable of doing more than they thought they could); Acquiring physical and psychological health benefits (improved fitness, muscle tone, muscle strength, mood, and energy levels); seeing participation in the program/ physical activity as playing a key role in recovering from cancer<br>-fostering feelings of normality<br>-allowing them to feel a sense of self-control over their life/ doing something actively to overcome complaints (fatigue, depression) or the medication.
