# Supplementary material for: Psychosocial Determinants of Lifestyle Change after a Cancer Diagnosis: A Systematic Review of the Literature
Source: Cancers (Basel). 2022 Apr 16;14(8):2026. doi: 10.3390/cancers14082026 (PMC9032592; doi:10.3390/cancers14082026)
Supplement: Supplementary file 1 [file cancers-14-02026-s001.zip › Table S8_MH.pdf]

Table S8. Overview of qualitative studies on psychosocial determinants of changes in diet only (n=9).

First author, (year), Country	Study design	Sample characteristics	Lifestyle change	Findings on psychosocial determinants
Adams & Glanville (2005)[121]<br><br>Canada	*Individual in-depth face to face semi-structured interviews*	N=6 women who were breast cancer survivors for 6 to 19 years.<br><br>Age Range: 43-62 years<br><br>Time since diagnosis: 6 - 19 years.	Dietary practices and changes since diagnosis	*Barriers:*<br>-Lack of information, at the time of diagnosis and later, about the role of diet in breast cancer, or about strategies they could use to deal with treatment and achieve health.<br><br>*Facilitators:*<br>-Women who believed that diet prevents a first diagnosis adopted change to prevent recurrence<br>- those who did not believe in the relationship between diet and breast cancer adopted change to improve health.<br>-During the treatment phase, women used food *to gain control* over the disease itself and/or to control negative treatment effects (Increasing physical strength and lessen negative treatment effects; Promoting healing; seeing food as a means of survival, needing good nutrition to regain health; using rituals and regimens to control the course of treatment).<br>-using food *to cope* with the stressful breast cancer treatment phase; food gave them energy and improved their frame of mind.<br>-Using food as a source of *comfort*: some foods made them feel better during treatment, including food a family member prepared, which was viewed as an act of kindness.<br>-Viewing food as a source of hope, to eliminate the cancer during treatment<br>-Post-treatment, women who believed that diet plays an integral role in preventing a first-time diagnosis and/or preventing recurrence derived a *sense of control* through diet.<br>-Post-treatment, women who did not believe in the relationship between diet and breast cancer (i.e., that behavior before diagnosis caused breast cancer) felt a lack of control over recurrence; they *adopted dietary change to improve health* rather than to prevent recurrence.
Avery et al. (2014)[102]<br><br>UK	*Multi-centre qualitative interview study;* semi-structured interviews with patients and their partners, and focus groups with stakeholders, including healthcare professionals.<br><br>Only results from patient and partner interviews described.<br><br>Partners were interviewed simultaneously or separately depending on the couples' preferences.	Men diagnosed with localized Prostate Cancer (n=37) and their (n=11) partners.<br><br>Interviews were conducted between 7–42 months post-diagnosis, in the participant's home or by telephone<br><br>Mean age patients 66.5 (54.4-75.4)	Dietary change in prostate cancer survivorship	*Barriers:*<br>*-Lack of dietary advice from their healthcare professionals* after prostate cancer diagnosis: *Information* needs: desire for more and better dietary information that may supporting survivorship, particularly among those on active surveillance programmes.<br>-Uncertainty about causes of prostate cancer and the potential role of diet in prostate cancer aetiology.<br>-Confusion and dissatisfaction with available dietary information and/or its contradictory nature.<br>-dietary change perceived as unnecessary due to 'curative' treatment<br><br>*Facilitators:*<br>-Psychological and general *health benefits*: making dietary changes to promote general or prostate health or facilitate coping, despite uncertainty about diet-prostate cancer links;<br>*-Preventing progression and/or recurrence* or to be 'fighting fit' for further treatment; curing prostate cancer; preventing other cancers.<br>-Positive psychological effects associated with *'doing something'* potentially beneficial to *manage/control* their disease.

				-*Partners* integral to implementing changes: Joint decision-making regarding dietary decisions and dietary change; partner-driven dietary changes.<br>-Receiving evidence-based *dietary advice/information* from *authoritative and reputable sources*, typically health professionals directly involved with their care (e.g. their primary care physician, hospital consultant or nurse), including information about diet-prostate cancer links<br>-*Prostate cancer diagnosis* often served as a *'wake-up call'* or incentive to consider dietary improvements to enhance general and prostate health after diagnosis<br>-Dietary change serving as a *coping* strategy: feeling they were *'doing something'* to help; focusing on dietary changes helped manage uncertainty and regain some control over disease and future survivorship; Positive psychological effects of 'doing something'<br>-Diet believed to cause Prostate Cancer<br>-Perceived relationship between prostate cancer treatment and dietary change: dietary change perceived as an adjunct therapy to active monitoring and radiotherapy.
Beagan & Chapman (2004)a[122]<br><br>Canada	*In-depth individual face to face semi-structured interviews*	Culturally diverse convenience sample of women aged 40 to 60 years, including breast cancer survivors (n = 29) who had completed treatment and women who had not had breast cancer (n = 32).<br><br>Only results from women with breast cancer described.<br><br>Time since diagnosis: 6 months - 15 years.	Changes since breast cancer diagnosis	*Barriers:*<br>-Resistance from family members to dietary changes (e.g., husbands dislike of the taste and smell of fish, husbands' preferences for high-fat foods, sauces, and gravies.)<br><br>*Facilitators:*<br>-Family members/ partners supporting healthful eating: (e.g., husband in charge of her vitamin regimen; daughter taught to eat a lower-fat diet, grown sons came over to help juice raw carrots)<br>-Believing that the diagnosis had shifted their priorities: *healthful eating takes on greater priority* than food preferences or maintaining social relations through catering to others' (family members) tastes.<br>-Breast cancer as "the big wake-up call," a major turning point that caused them to rethink many aspects of their lives, including prioritizing themselves first instead of putting everyone else first, and revising the place of family influences in prioritizing competing values for food choice.<br>-Being intrinsically motivated, regardless of family members
Beagan & Chapman (2004)b[123]<br><br>Canada	*In-depth face-to-face individual semi-structured interviews*	Culturally diverse convenience sample of women (N = 30) aged 40 to 60 years diagnosed with breast cancer 6 months to 15 years previously.	Changes since breast cancer diagnosis	*Barriers:*<br>-Believing that diet could cause or contribute to breast cancer, but that it was not a factor in their own case because they had already eaten "well" prior to diagnosis.<br>-Not believing that diet causes cancer nor that a change in diet could prevent occurrence or recurrence of cancer.<br>-perceiving dietary change as 'too late to bother changing' or already having adopted what they considered more healthful eating.<br>-Resistance from family members to diet changes, affecting what the whole family ate (e.g. Children being picky and preferring junk food).<br>-Living alone: no one to support or encourage changes, and little incentive to care much about how they ate.<br>-Financial issues: not being able to afford healthy products<br>-*Unfamiliarity with healthy eating traditions*: difficulties with breaking unhealthy cultural food patterns, even when they believed them to be detrimental to their overall health and/or their risk of cancer recurrence; distrusting vegetarianism, which was culturally unfamiliar.<br>-*Concurrent health concerns*: lack of energy to cook healthy because of fibromyalgia; eating more dietary fiber and more cruciferous vegetables were difficult with Crohn's disease._

				-Employment-related factors: difficult to maintain a healthful diet when work caused to eat out a lot; shift work interfered with their ability to eat the way they wished; working full time and living alone, and eating prepared, processed foods that could be made quickly for convenience<br><br>*Facilitators:*<br>-Believing that *diet is related to breast cancer* and that *dietary changes may reduce the probability of recurrence*.<br>-Family support: Encouragement by family members: husbands or partners, children, parents, and/or siblings.<br>-Having someone to share cooking responsibilities<br>-Living alone: no one to resist dietary changes; not having to negotiate changes with anyone.<br>-Employment-related factors: diet changes facilitated by retirement, shortly after her diagnosis<br>-Familiarity with different eating traditions: a healthy low-fat diet high in fiber, fruit, and vegetables is familiar to women of Asian heritage<br>-Concurrent health concerns: self and others, such as already having a diet prescribed to control diabetes and control her weight; already having a lower-fat, lower meat diet to reduce body weight and the risk of heart disease; already having changed diet to address her husband's diabetes.
Forslund et al. (2020)[138]<br><br>Sweden	*Qualitative descriptive interview study with semistructured face-to-face interviews*.<br><br>This study was conducted alongside a randomized controlled trial	N=15 men with prostate cancer who were or had been participating in the nutrition intervention group of a randomized controlled trial during radiotherapy<br><br>Median age 70 (62-78)	During a 26 month nutrition intervention during radiotherapy	*Barriers:*<br>-Feeling limited, both regarding full meals and specific food items.<br>-the timing of the intervention<br>-unmet expectations of dietary advice<br>-loss of motivation: motivation and adherence subsided over time, gradually returning to old food habits.<br>-Increased costs: Lactose-free and lactose-reduced dairy products were experienced as a bit more expensive than regular dairy products.<br>-shopping for food became more difficult, having to drive to more than one grocery store to find the food items recommended in the nutrition intervention.<br>-wanting to decide for themselves / the importance of *making their own choices* regarding what to eat. (sometimes chose to make exceptions, regardless of consequences such as bowel symptoms).<br>-Specific situations, like travel or social events<br>-The desire not to bother others with the preparation of special foods according to the nutrition intervention.<br><br>*Facilitators:*<br>-to avoid bowel symptoms<br>-prior knowledge of (lactose-free or lactosereduced dairy) products, and the fact that they had already been introduced into the household.<br>-a general interest in food<br>-dietary information/ advice were informative and supportive when shopping for food (recommended vs. recommended).<br>-anticipated benefits: reduced bowel symptoms from the radiotherapy treatment; belief that diet could improve well-being.<br>-receiving dietary advice gave a feeling that somebody cared<br>-they appreciated that they could do something to affect their own situation.<br>-New knowledge, acquiring new habits (Reading the ingredients lists on packages), discovery of new products was described as a positive experience.<br>-Recipes and pre-set meal suggestions

				-Social support: good help and support from their wives and other relatives (planning of meals and cooking at home by wife, who made sure their husbands followed the dietary advice).<br>-perceiving dietary adjustments as easy: no or only small need for change regarding food choices, daily routines, and meals, and they could continue with their usual diet to a large extent. (small need for behaviour change)
Kassianos et al. (2015)[103]<br><br>UK	*Semi-structured telephone interviews*	N=8 men diagnosed with prostate cancer within the preceding 5 years.<br><br>Mean age 64.9; Range 55-76 years	Dietary change since prostate cancer diagnosis.	*Barriers:*<br>-Health professionals' lack of knowledge and credible, evidence-based information provision about diet; Lack of availability of credible dietary information and advice (from health care providers)<br>-Feeling a need to make dietary changes from their own decision-making rather than from 'being told to'.<br><br>*Facilitators:*<br>-Perceiving dietary change as an 'obvious' initial context for agentic action (*doing something oneself,* taking *control* over ones dietary changes)<br>-Shift in participants' relationships with food /'re-approaching' food after diagnosis, re-evaluation of the function, role and meaning of food; post-diagnosis vigilance to health-enhancing and health-threatening foods; perceptions of food changed from a resource to fulfil physiological requirements pre-diagnosis to a source of justified pleasure postdiagnosis, from unthinking use to mindful, appreciative engagement<br>-Perceived nature and importance of dietary change: Considering lifestyle change important for the *management of their condition* and for *reducing the risk of recurrence.*<br>-the aspiration for a return to pre-diagnosis normality<br>-*subjective benefits*, such as feeling good, and an ongoing lack of possible recurrence indicators.<br><br>-Family influences on dietary action: social roles within family environments; participants' wives and partners played an important role in food choice and preparation, dependence on partners in relation to dietary change rather than shared decision-making.
Locher et al. (2009)[115]<br><br>USA	*In-depth, semi-structured face-to-face interviews*	N=30 diagnosed community-dwelling older adults with pancreatic, colon, breast, lymphoma, skin, and head and neck cancers, and their caregivers (interviewed separately)<br><br>Mean age 79 years, Range of 70–99 years.<br><br>56,7% Female; N=17	Post-diagnosis	*Barriers:*<br>-Cancer- and treatment-related alterations in self-identity due to changes in their bodies (physiologic and anatomic sequelae associated with the cancer and its treatment), in their sense of taste, and in the manner in which they must eat caused cancer patients to experience frustration and embarrassment (shame), which led to reduced nutritional intake. Some patients refused to eat in the presence of others as it took them a much longer time to eat food, which made them feel as a burden; feeling embarrassed by changes in food preferences and in the way they physically ate.<br>-Not discussing food and eating habits with their physicians. Food and Eating Problems Not Shared With Physicians, despite the fact that some patients and caregivers wanted such information. Both patients and caregivers believed that such matters were not the responsibility of the doctor; Not discussing beliefs and behaviors associated with weight loss with physicians or other healthcare providers<br>-no longer being able to consume the foods that once symbolically conveyed meaning to others regarding their social status (e.g. consumption of red meat in men)<br>-Not being able to consume foods that one typically consumed while eating out interferes with the commensal aspects of meals (normative expectations regarding what,

				when, and with whom particular foods and food combinations may be eaten).<br>-social factor contributing to undereating: the belief held by both patients and caregivers that weight loss is a good thing/ a positive health outcome of the cancer.<br><br>*Facilitators:*<br>-the perception of dietary intake as a way for patients to *maintain control* over their lives when all else seems out of control; control over the body, the mind, and therefore over identity, the self.<br>-to avoid getting diabetes<br>-Perceived Health-Promoting Qualities of Foods<br>-believing that weight loss is desirable, several patients reported going on diets to keep weight off they had lost, which was positively reinforced by family caregivers, who believed that patients ought to lose weight.
Mróz & Robertson (2015)[104]<br><br>UK	*Semi-structured, in-depth interviews*	N=28 men diagnosed with early to mid-stage prostate cancer at least 6 months before and within the last 5 years and their partners (n=14)	After prostate cancer diagnosis	*Barriers:*<br>-Shift in domestic food dynamics (from where she controlled food decision-making and provision to where he began to influence dietary decision-making) leads to tensions because it disrupts traditional gender food roles; balancing of his need/desire to be involved in global dietary decision-making with her need for control over practical aspects of daily food provision<br>-the pragmatic, practical, work-based daily task of food preparation remaining the responsibility of the female partner.<br>-complexity of food negotiations with partners (e.g. regarding meat consumption) reflecting a form of gender expression and/or control.<br>-finding an appropriate balance between new dietary regimens and living an enjoyable life requires major and on-going negotiation.<br>-Lack of knowledge about how/where to look for information<br>-Lack of interest in food<br>-Lack of skills<br>-the responsibility for maintenance of food changes often fell on partners (women): maintenance depended on the partner's on-going monitoring or 'control' of new food practices, ensuring men kept to the diet changes they had agreed upon. Women felt the burden of responsibility in this stereotypical 'mother/child' gender dynamic<br>-tension in differing views on moderation and her role in directing his diet (she was critical of his food choices and he was critical of her 'obsessive' approach to eating healthily).<br><br>*Facilitators:*<br>-Seeking diet information<br>-Partners agreeing to his suggested diet changes and changing her food provision and her own eating practices to reduce tension and "make life easier".<br>-man taking a leadership role in family food; a cultural field that previously was the realm of the female partner for the couple (Taking control or autonomy in decision-making)<br>-Interest and knowledge of food and cooking<br>-Defending dietary changes (e.g., eating vegetables and salads) on medical grounds, seen as crucial to his survival and therefore justifiable<br>-Having his partner filter information helped him to avoid worrying about having prostate cancer.<br>-Importance of relaxing diet 'rules' and having occasional 'treats' to preserve enjoyable aspects of life.
Wong et al. (2021)[139]	*Individual and group interviews* from a	N=55 adult colorectal cancer survivors who	During 12-month diet intervention	*Barriers:*

China	large, multicentre, randomised controlled trial that evaluated two 12- month behavioural interventions.<br><br>Participants were randomised into intervention groups (physical activity intervention, dietary intervention or both interventions) or control groups.	had received curative treatment and completed a 12- month diet intervention<br><br>Mean age was 64 years (SD 9.9)<br><br>47.3% Female (n=26)		-Traditional Chinese cultural beliefs and practices (e.g., the belief that rice and meat are the main sources of energy and nutrition); difficulties in adjusting their accustomed values and habits.<br>-Feelings of isolation while eating with others. Eating together with family members and friends is a social event and builds relationships and helps people feel more socially connected.<br>-dilemma between staying on a healthy diet (not eating what everyone else is eating) and maintaining harmony with others (participating in Traditional Chinese dinners focused on sharing and enjoying food together with friends and relatives, although not in line with diet).<br>-Not wanting to challenge the host with many specific dietary restrictions.<br>-being exposed to many traditionally meaningful but high-carbohydrate food during Chinese festivals and having difficulties resisting them.<br>-Perceiving it as impolite to reject the snacks and alcohol prepared by relatives and friends, for example during Chinese New Year.<br><br>*Facilitators:*<br>-individual commitment to dietary change<br>-awareness of the importance of healthy diet after treatment because of the severity of their disease and the possibility of recurrence.<br>-working with healthcare professionals during the journey<br>-adaptive strategies in interpersonal contexts: finding strategies to adhere to diet, while still eating with others: using innovative strategies to overcome the challenge of being exposed to high-carbohydrate food during Chinese festivals (For example, some made their own healthy version of rice dumplings in Dragon Boat Festival).<br>-gaining knowledge on a healthy diet through informative resources (including mailed pamphlets, e-mails and phone messages) given by healthcare professionals as part of the intervention.<br>-Recognizing the possible detrimental health impact of the traditional festival foods<br>-additional benefits of dietary changes: experienced improvement in general well- being<br>-Receiving evidence-based information from healthcare professionals<br>-Receiving emotional support from healthcare providers.<br>-Participants' confidence and determination
