# Supplementary material for: Psychosocial Determinants of Lifestyle Change after a Cancer Diagnosis: A Systematic Review of the Literature
Source: Cancers (Basel). 2022 Apr 16;14(8):2026. doi: 10.3390/cancers14082026 (PMC9032592; doi:10.3390/cancers14082026)
Supplement: Supplementary file 1 [file cancers-14-02026-s001.zip › Table S9_MH.pdf]

Table S9. Overview of qualitative papers on psychosocial determinants of changes in smoking only (n=4).

First author, (year), Country	Study design	Sample characteristics	Lifestyle change	Findings on psychosocial determinants
Li et al. (2014)[140]<br><br>China	*Face to face individual semi-structured interviews*	N=40 current (n=20) and ex-smoking (n=20) patients diagnosed with lung, liver, stomach, nasopharynx, or colorectal cancer at least 6 months ago<br><br>Mean age 57.2 (SD= 13.0)<br><br>2.5% female<br><br>Mean time since diagnosis 2.7 years; Range 5 months - 9 years	After cancer diagnosis	*Barriers:*<br>-Lack of knowledge of the association between smoking and cancer,<br>the risks of continued smoking, and the benefits of quitting in relation to treatment efficacy and cancer prognosis.<br>-Not perceiving any benefits of quitting.<br>-Nicotine dependency<br>-Experiencing a strong desire to smoke<br>-Finding it very hard to abstain from smoking, particularly when they were alone or felt bored.<br>-Perceiving smoking as a social norm and as a tool for communication and connecting with friends.<br>-Feeling impolite or embarrassed to reject a cigarette from a friend<br><br>*Facilitators:*<br>-Realizing a causal relationship between smoking and cancer, and the belief that continuing to smoke would be further detrimental to health.<br>-Perceived benefits of quitting: the belief that quitting could promote treatment efficacy and improve cancer prognosis.<br>-Quitting smoking saves money, and there was a need to save money after cancer<br>-Support to quit smoking from family (advice from worried family members to quit). They believed that quitting was something they could do for their families.
Smith et al. (2021)[133]<br><br>Australia	*Mixed methods, sequential explanatory design.*<br><br>Prior to commencement of treatment, baseline survey data were collected around the time of diagnosis. Participants identified as current smokers at baseline were followed up to determine smoking status after treatment and asked to participate in one-on-one, semi-structured interviews.<br><br>Interviews were conducted face-to-face (n = 11) or over the phone (n = 4).	N=64 head and neck (squamous cell) cancer patients, of which 15 participated in one-on-one interviews (5 ceased and 10 continued smokers)<br><br>Female 13.3% (n=2)	Unspecified	*Barriers:*<br>-Lack of willpower<br>-Being unemployed or not able to work after treatment<br>-Residing with other smokers<br>-Depression<br>-Isolation associated with returning to a rural location after the completion of treatment.<br>-Still consuming alcohol in large quantities or smoking marijuana.<br>-Using marijuana as a substitute for nicotine use.<br>-Being more concerned about harmful effects of their occupational exposure than the effects of cigarette smoking<br>-The stress of being away from home (in hospital).<br><br>*Facilitators:*<br>-Not wanting to compromise their treatment<br>-Being too unwell to smoke because of the side effects of radiotherapy -Fear of being discovered by the exhaled carbon monoxide readings<br>-The cancer diagnosis as initial motivating factor<br>-Treatment and its associated side effects<br>-Health practitioners advice<br>-Willpower<br>-Pharmacological and non-pharmacological cessation aids<br>-Helpful nature of the education and motivation given by healthcare professionals in their treating team<br>-Supportive family encouraging smoking cessation<br>-Living alone with no temptations to smoke<br>-Being away from home for treatment, as they were removed from the environment in which their bad habits had been ingrained.

				-Removing the association between alcohol and smoking.<br>-Patient's health knowledge and beliefs surrounding the role of smoking in cancer: able to identify that smoking plays a role in the formation of cancer and the harmful effects of continued smoking after diagnosis.<br>-Prior education regarding addictions and withdrawal through occupational interventions.
Weiss et al. (2020)[124]<br><br>Canada	*Individual interviews* (n=19) and focus groups (n=3) with health care professionals (n=16) ; Only results of patients described.	N=19 lung cancer patients; current or former smokers<br><br>Mean age 74.5 years<br>Range: 51– 87 years<br><br>Diagnosed from 1 to 5 years before the time of the interviews<br><br>Participants had smoked more than 20 years, with three participants still smoking at the time of the interview; All participants had tried at least one quit attempt.	Not specified	*Barriers:*<br>-difficulty in quitting<br>-uncertainty on how to approach quitting<br>-time left/stage of cancer<br>-health care providers' lack of inquiry<br><br>*Facilitators:*<br>-knowledge (eg, about the impact of smoking on illness and why they should not smoke)<br>-individual decision to quit (eg, motivators)<br>-social unacceptability of smoking<br>-family impact (influence, support, and enabler)<br>-harm recognition<br>-accomplishment in quitting<br>-the importance of health care providers addressing smoking<br>-health care providers' view of the relationship between smoking and lung cancer diagnosis
Wells et al. (2017)[105]<br><br>UK	*In-depth qualitative interviews with 29 patients, 14 family members, and 24 health professionals. Results from health professionals not described.*	Three samples were recruited: cancer patients, family members, and health professionals.<br><br>N=29 patients<br>44.8% female (n=13)<br><br>Patients were more than 2 weeks but less than 3 years from diagnosis of lung, head & neck, colorectal or cervical cancer, and currently smoking or smoked until diagnosis.<br><br>Fifteen out of 29 patient participants and 11 out of 14 family members were current smokers at the time of interview. The others all reported having stopped smoking at or around the time of diagnosis.	First 6 months after a cancer diagnosis	*Barriers:*<br>- Negative views about the appropriateness of UK National Health Service smoking cessation services<br>-Lack of meaningful discussions with health professionals about smoking.<br>-Stress experienced following a diagnosis; smoking as a way of helping to cope with stress.<br>-Desire to maintain personal control and choice over smoking behavior, a sense of 'normal' self<br>-Resentment of 'nagging' or pressure by relatives to stop smoking.<br>-Limited perceptions of and uncertainty about the risks and consequences of smoking and the health benefits of stopping (e.g., too late to stop smoking in the face of terminal illness and in light of their smoking history).<br>-Thoughts of stopping smoking aroused a stress response which they would have difficulty dealing with.<br>-Lack of time to attend smoking cessation services regularly due to the various demands on patients' time following a cancer diagnosis, particularly during treatment periods.<br>-Lack of privacy in current cessation services delivered in pharmacies and the repetitiveness of the generic 'stop smoking' message which, perhaps, was not effective for those already affected by cancer.<br><br>*Facilitators:*<br>-perceived reduction of the social acceptability of smoking<br>-caring responsibilities<br>-cost<br>-Using community-based pharmacy smoking cessation services: receiving good quality and person-centred relevant information and receiving support from smoking cessation facilitators with effective interpersonal skills.<br>-Willpower<br>-Specific, directly-targeted hospital-based cessation services, integrated with cancer treatment and care, enabling patients and families to combine participation with routine hospital attendance would encourage successful uptake.
